# Supplementary material for: Observational retrospective study calculating health service costs of patients receiving surgery for chronic rhinosinusitis in England, using linked patient-level primary and secondary care electronic data
Source: BMJ Open. 2022 Feb 8;12(2):e055603. doi: 10.1136/bmjopen-2021-055603 (PMC8830221; doi:10.1136/bmjopen-2021-055603)
Supplement: Supplementary data [file bmjopen-2021-055603supp001.pdf]

Title:

Observational retrospective study calculating health service costs of patients receiving surgery for chronic rhinosinusitis in England, using linked patient-level primary and secondary care electronic data

Authors:

Caroline S Clarke* (caroline.clarke@ucl.ac.uk), Elizabeth Williamson, Spiros Denaxas, James R Carpenter, Mike Thomas, Helen Blackshaw, Anne GM Schilder, Carl Philpott, Claire Hopkins and Stephen Morris, on behalf of the MACRO programme team

Supplementary Materials

A. Section A – Codelists

i. CRS diagnostic terms – definite CRS

The following codelist was developed to identify patients with chronic rhinosinusitis, according to "definite" diagnostic events. Patients with one or more of these diagnoses or procedures recorded during follow-up were considered to be definite CRS cases, with the date of diagnosis taken to be the date of the first such diagnosis or procedure.

Code type	ICD-10 Code		Description ("definite" CRS diagnosis)
ICD-10	J32		Chronic sinusitis
	J320		Chronic maxillary sinusitis
	J321		Chronic frontal sinusitis
	J322		Chronic ethmoidal sinusitis
	J323		Chronic sphenoidal sinusitis
	J324		Chronic pansinusitis
	J328		Other chronic sinusitis
	J329		Chronic sinusitis, unspecified
	J33		Nasal polyp
	J330		Polyp of nasal cavity
	**MEDCODE**	**Read code**	
MEDCODE with corresponding Read Code	811	7406000	Nasal polypectomy
	848	7411200	Intranasal antrostomy
	977	H110.00	Polyp of nasal cavity
	1673	7412100	Intranasal ethmoidectomy
	1674	H132.00	Chronic ethmoidal sinusitis
	2257	H13..00	Chronic sinusitis
	4433	H130.00	Chronic maxillary sinusitis
	4686	H11..00	Nasal polyps
	5437	H13z.00	Chronic sinusitis NOS
	6411	2D33.00	O/E - nasal polyp present

Clarke CS, *et al. BMJ Open* 2022; 12:e055603. doi: 10.1136/bmjopen-2021-055603

	6491	7416	FESS/Therapeutic endoscopy of nose and sinus
	7243	7.42E+03	FESS/Post operative division of adhesions
	8220	7416300	FESS/Uncinectomy
	10546	H13..11	Chronic rhinosinusitis
	11744	H11y100	Polyp of ethmoidal sinus
	14749	H110z00	Polyp of nasal cavity NOS
	14888	H11z.00	Nasal polyp NOS
	15163	H131.00	Chronic frontal sinusitis
	16626	7416D00	FESS - post operative removal of polyps (local anaesthetic)
	17173	H135.00	Recurrent sinusitis
	18083	7416C00	FESS - post operative suction clearance (local anaesthetic)
	18869	7416900	FESS/Antrostomy via middle meatus
	19742	H11y.11	Nasal sinus polyps
	20806	7415.11	FESS - diagnostic nasal antroscopy
	20832	7416500	FESS/Anterior ethmoidectomy
	21213	7412700	Radical frontal sinus antrostomy
	21923	7416.11	FESS/Therapeutic nasal antroscopy
	27869	7412800	Frontal sinusotomy NEC
	30990	S832300	Open wound of nasal sinus
	33709	7415z00	FESS/Diagnostic endoscopy of nose or sinus NOS
	33922	7416700	FESS/Anterior and posterior ethmoidectomy
	34165	7412300	Transantral ethmoidectomy
	34973	7416z00	FESS - therapeutic endoscopy of nose or sinus NOS
	35389	7416B00	FESS/Excisional surgery to middle turbinate
	35897	H11y200	Polyp of maxillary sinus
	37481	7416800	FESS/Sphenoidectomy
	39501	H13y000	Chronic pansinusitis
	42166	7416F00	Functional endoscopic sinus surg - polypectomy nasal sinus
	45995	7416y00	FESS - therapeutic endoscopy of nose or sinus OS
	46336	7416A00	FESS/Antrostomy via inferior meatus
	48703	H133.00	Chronic sphenoidal sinusitis
	49348	7416600	FESS/Anterior ethmoidectomy and frontal recess dissection
	49548	H13y.00	Other chronic sinusitis
	54375	H13yz00	Other chronic sinusitis NOS
	59019	7416400	FESS/Uncinectomy and excision of bulla
	59339	7415200	FESS - diagnostic antroscopy via middle meatus
	61281	H11y300	Polyp of sphenoidal sinus
	62936	7416200	FESS/Therapeutic antroscopy via middle meatus
	63733	Hyu2200	[X]Other chronic sinusitis
	64359	7412y00	Other specified operation on frontal sinus
	68003	7M1B000	Functional endoscopic sinus surgery
	69714	7406700	Nasal polypectomy using auto-debrider
	86064	7M1B100	Functional endoscopic nasal surgery
	**OPCS code**		
**OPCS**	E081		Polypectomy of internal nose
	E133		Intranasal antrostomy
	E142		Intranasal ethmoidectomy
	Y761		Functional endoscopic sinus surgery
	Y762		Functional endoscopic nasal surgery

Clarke CS, *et al. BMJ Open* 2022; 12:e055603. doi: 10.1136/bmjopen-2021-055603

ii. CRS surgery terms – definite or very likely CRS surgery

The subgroup of surgical patients were those who had also undergone surgery that was "definitely" or "very likely" to have been for CRS and the codelist for "very likely" CRS surgery is given in a second table below.

Code type	Code	Description ("very likely" CRS surgery)
OPCS	E13	Other operations on maxillary antrum
	E131	Drainage of maxillary antrum NEC
	E132	Excision of lesion of maxillary antrum
	E136	Puncture of maxillary antrum
	E138	Other specified other operations on maxillary antrum
	E139	Unspecified other operations on maxillary antrum
	E14	Operations on frontal sinus
	E144	Transantral ethmoidectomy
	E147	Median drainage of frontal sinus
	E148	Other specified operations on frontal sinus
	E149	Unspecified operations on frontal sinus
	E15	Operations on sphenoid sinus
	E151	Drainage of sphenoid sinus
	E152	Puncture of sphenoid sinus
	E158	Other specified operations on sphenoid sinus
	E159	Unspecified operations on sphenoid sinus
	E16	Other operations on frontal sinus
	E161	Frontal sinus osteoplasty
	E162	Drainage of frontal sinus NEC
	E168	Other specified other operations on frontal sinus
	E169	Unspecified other operations on frontal sinus

iii. Polyp diagnostic terms

The codelist for determining whether a patient was categorised as being in the positive-polyps subgroup or the unknown-polyps subgroup is below. Those patients exhibiting one or more of these codes were classified as "polyps positive" and those lacking these codes were "polyps unknown".

Code type	Code	Description ("positive polyps")
ICD-10	J33	Nasal polyp
	J330	Polyp of nasal cavity
	J331	Polypoid sinus degeneration
	J338	Other polyp of sinus
	J339	Nasal polyp, unspecified
MEDCODE	11744	Polyp of ethmoidal sinus
	14749	Polyp of nasal cavity NOS
	14888	Nasal polyp NOS
	16626	FESS - post operative removal of polyps (local anaesthetic)
	19742	Nasal sinus polyps
	24535	Removal of antrochoanal polyp
	35897	Polyp of maxillary sinus
	42166	Functional endoscopic sinus surg - polypectomy nasal sinus
	4341	Antral (maxillary) polyp
	44518	Other polyp of sinus NOS
	44934	Polypoid sinus degeneration
	4686	Nasal polyps
	50528	Polypoid sinus degeneration NOS
	61281	Polyp of sphenoidal sinus
	6411	O/E - nasal polyp present
	69714	Nasal polypectomy using auto-debrider
	811	Nasal polypectomy
	91482	Polyp of frontal sinus
	977	Polyp of nasal cavity
OPCS	E081	Polypectomy of internal nose

iv. Flowchart illustrating the application of the codelists in this section of the Supplementary Materials

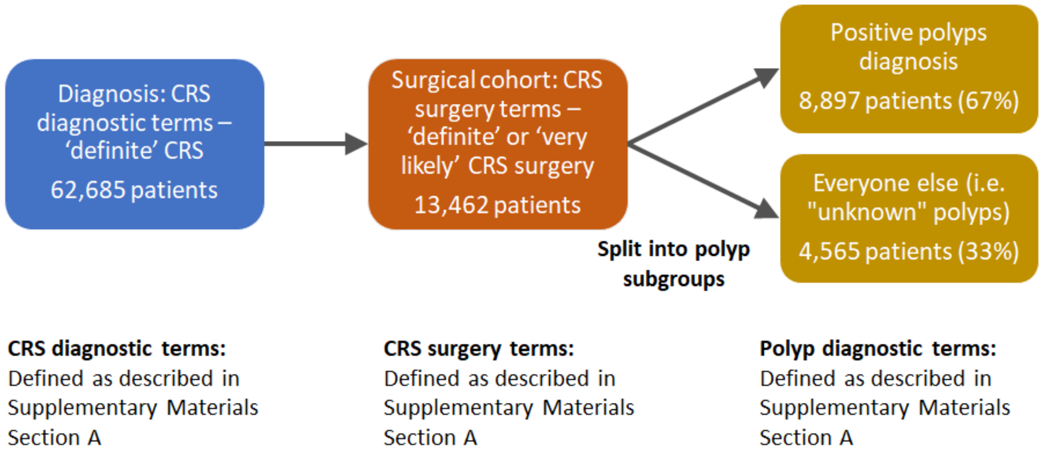

B. Section B – Unit costs and other input information

**NHS Reference Cost categories (2017-18 prices)**	**Inpatient (APC) events**		**Outpatient (OP) events**	
	**Elective Inpatient unit cost (£)**	**Day Case unit cost (£)**	**Outpatient unit cost (£)**	**OPCS/ICD-10 code(s)**
Complex sinus procedure	£3,972.25	£2,383.70	£114.23	E147, E152, E153
Major Sinus Procedures	£2,918.76	£2,298.06	£118.52	E131, E132, E141, E143, E144, E146, E148, E149, E151, E154, E158, E159, E161, E162, E168, E169, E171, E172
Intermediate Sinus Procedures	£2,536.77	£1,920.27	£145.73	E133, E138, E139, E142, E178, E179, Y403, Y761, Y762, Z238, Z239
Minor Sinus Procedures	£2,320.64	£1,654.96	£113.14	E136
Intermediate Nose Procedures	£2,241.99	£1,128.15	£164.98	E058, E059, E088, E089, E108, E109, Y408, Y409, Z228, Z229
Nasal Polypectomy	£2,193.28	£1,587.09	£130.83	E081
Minor Nose Procedures	£1,876.76	£1,325.83	£148.90	E061, E062, E063, E064, E068, E069
Excision or Biopsy, of Lesion of Internal Nose	£1,748.75	£1,054.22	£144.62	E082, E101, E134
Other Specified Diagnostic Imaging of Other Sites, 19 years and over	£214.29	£214.29	£214.29	U064, U068, U069, U217
Computerised Tomography Scan of One Area, without Contrast, 19 years and over	£88.21	£88.21	£88.21	U051, U061, U212

Table B1. Unit costs, inpatient and outpatient health care contacts, from published NHS Reference Costs *[23]*. **Prices in 2017-2018 £.**

**Mean duration of visit, minutes**	**GP**	**Nurse/Other**	**References**
Standard consultation visit	9.22	9.22	[24]
Specific clinic or home visit	17.20	17.20	[25]
Telephone call	7.10	7.10	[25]
**Unit cost per visit**			
Standard consultation visit	£37.34	£6.45	[24]
Specific clinic or home visit	£69.66	£12.04	[24]
Telephone call	£28.76	£4.97	[24]

Table B2. Unit costs, primary care consultations. Including information on the duration of a contact, and the unit cost calculated on that basis. Prices in 2017-2018 £.

**Antibiotics**	**Unit cost**
Cephalosporin	£4.97
Macrolide	£6.64
Metronidazole	£2.13
Penicillin	£1.42
Quinolone	£2.45
Tetracycline	£8.57
Other antibiotic	£4.36
**Non-antibiotics**	**Unit cost**
Corticosteroids	£8.08
All other drugs	£4.69

Table B3. Unit costs of primary care medications, using the British National Formulary (BNF) online *[26]***. Prices in 2017-2018 £.**

C. Section C – Numbers of patients "at risk" of a primary care, outpatient, or inpatient care event, centred on surgery date at midpoint of Q0

Years from surgery	-10	-8	-6	-4	**-2**	**0**	**2**	4	6	8	10
Quarters from surgery	-40	-32	-24	-16	**-8**	**0**	**8**	16	24	32	40
**Numbers "at risk" of primary care consultation/prescription event**											
**Positive polyps**	1551	2489	3606	4995	**6697**	**9056**	**7028**	5443	4109	2896	1897
**Unknown polyps**	812	1250	1773	2451	**3289**	**4406**	**3199**	2365	1689	1182	783
**All patients**	2363	3739	5379	7446	**9986**	**13462**	**10227**	7808	5798	4078	2680
**Numbers "at risk" of inpatient care event**											
**Positive polyps**	1451	2362	3434	4804	**6460**	**8689**	**6713**	5242	4048	2930	2007
**Unknown polyps**	768	1198	1717	2360	**3163**	**4205**	**3239**	2408	1744	1247	833
**All patients**	2219	3560	5151	7164	**9623**	**12894**	**9952**	7650	5792	4177	2840
**Numbers "at risk" of outpatient care event**											
**Positive polyps**	0	0	0	0	**1135**	**8755**	**2962**	9	0	0	0
**Unknown polyps**	0	0	0	0	**633**	**4242**	**1234**	5	0	0	0
**All patients**	0	0	0	0	**1768**	**12997**	**4196**	14	0	0	0

Table C1. Numbers of patients "at risk" of a primary care, outpatient, or inpatient care event, centred on surgery date.

Clarke CS, *et al. BMJ Open* 2022; 12:e055603. doi: 10.1136/bmjopen-2021-055603

D. Section D – Mean inpatient costs per person-quarter, centred on surgery date at midpoint of Q0

Polypectomy costs were not split into DC and EL categories according to the length of stay as that regression did not converge, so instead a weighted cost was used according to the proportion of DC and EL polypectomies (32.8% DC and 67.2% EL).

Quarters after surgery (surgery date is at centre of Q0)	CT/other imaging, DC	Minor nose incl. biopsy, DC	Int nose and Minor sinus, DC	Int/Major/Complex sinus, DC	CT/other imaging, EL	Minor nose incl. biopsy, EL	Int nose and Minor sinus, EL	Int/Major/Complex sinus, EL	Polypectomy	TOTAL
**-8**	0.00	0.35	0.00	0.61	0.08	0.57	0.00	0.00	0.00	**1.62**
**-7**	0.03	0.00	0.00	0.00	0.08	0.00	0.00	1.56	0.00	**1.66**
**-6**	0.08	0.00	0.00	0.00	0.08	0.00	0.67	0.00	0.00	**0.82**
**-5**	0.07	0.32	0.00	1.10	0.27	2.07	0.65	1.46	0.00	**5.93**
**-4**	0.14	0.00	0.42	0.53	0.21	0.50	0.00	0.70	0.00	**2.52**
**-3**	0.05	0.00	0.41	0.00	0.23	0.96	0.00	0.68	0.00	**2.33**
**-2**	0.04	0.28	0.00	0.99	0.13	0.46	0.00	3.28	0.00	**5.20**
**-1**	0.02	0.82	0.38	0.48	0.34	0.89	0.00	3.79	0.00	**6.73**
**0**	**0.16**	**8.62**	**43.86**	**243.32**	**0.86**	**29.10**	**110.97**	**680.49**	**0.00**	**1117.37**
**1**	0.00	0.00	0.38	3.36	0.08	0.89	1.11	5.74	0.00	**11.56**
**2**	0.00	0.28	1.17	1.49	0.02	0.00	0.00	3.96	0.00	**6.92**
**3**	0.00	0.29	1.22	1.54	0.02	0.00	0.60	4.10	0.00	**7.77**
**4**	0.00	0.30	0.42	1.59	0.02	0.00	1.84	9.86	0.00	**14.04**
**5**	0.00	0.63	0.87	1.65	0.02	0.00	0.64	1.46	0.00	**5.28**
**6**	0.00	0.00	0.45	1.71	0.03	0.53	0.66	3.78	0.00	**7.15**
**7**	0.00	0.00	1.87	2.36	0.03	0.55	0.69	5.49	0.00	**10.98**
**8**	0.00	0.00	0.48	1.22	0.03	0.57	0.71	2.44	0.00	**5.45**

Table D1. Mean inpatient costs (Admittee Patient Care dataset from Hospital Episode Statistics) per person-quarter (2017-2018 £) - polyps unknown. DC = Day Case; EL = Elective Inpatient; CT = computed tomography; Int = intermediate

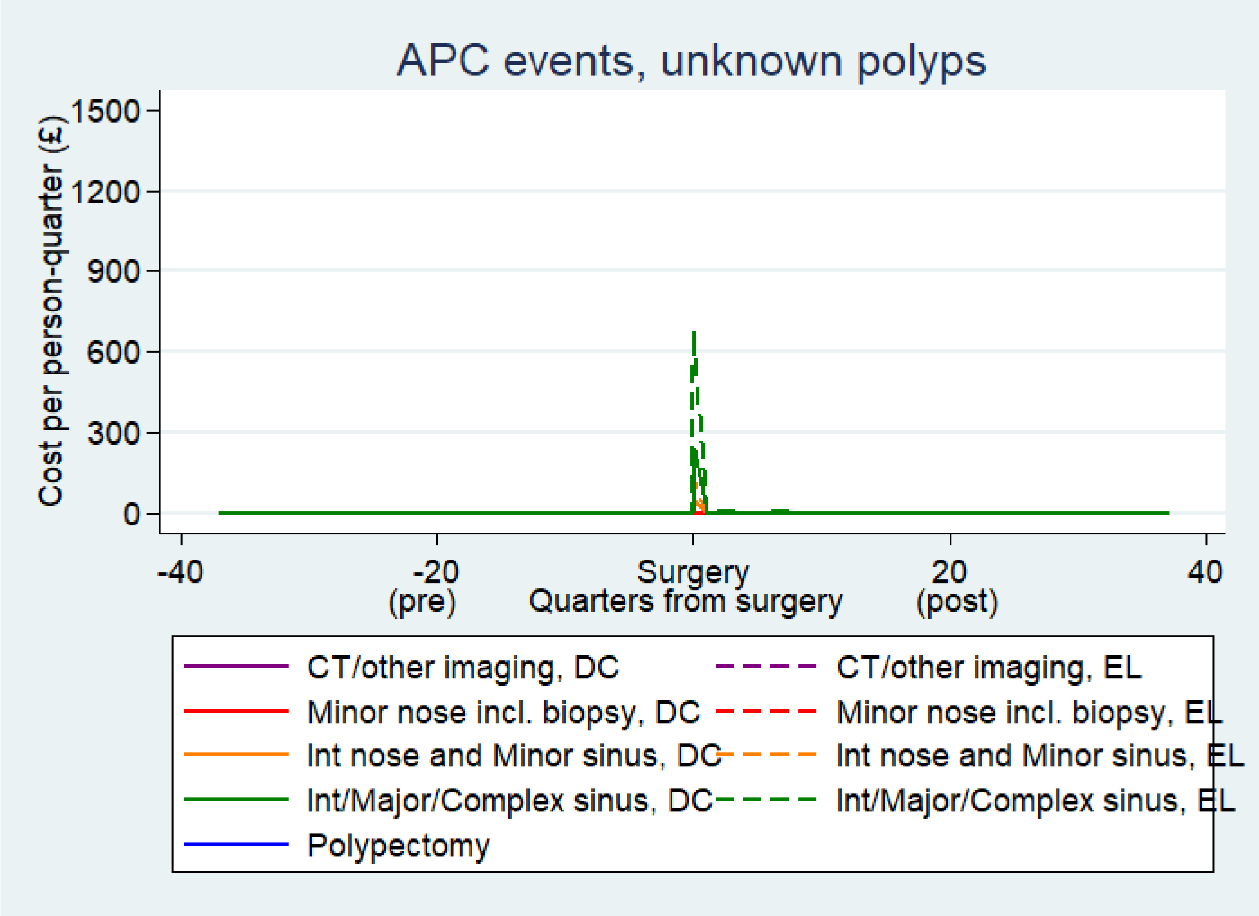
APC events, unknown polyps
Cost per person-quarter (£)
1500
1200
900
600
300
0
-40
-20
(pre)
Surgery
Quarters from surgery
20
(post)
40
CT/other imaging, DC
CT/other imaging, EL
Minor nose incl. biopsy, DC
Minor nose incl. biopsy, EL
Int nose and Minor sinus, DC
Int nose and Minor sinus, EL
Int/Major/Complex sinus, DC
Int/Major/Complex sinus, EL
Polypectomy

Quarters after surgery (surgery date is at centre of Q0)	CT/other imaging, DC	Minor nose incl. biopsy, DC	Int nose and Minor sinus, DC	Int/Major/Complex sinus, DC	CT/other imaging, EL	Minor nose incl. biopsy, EL	Int nose and Minor sinus, EL	Int/Major/Complex sinus, EL	Polypectomy	TOTAL
**-8**	0.00	0.00	0.00	0.30	0.09	0.00	0.00	0.00	0.00	**0.39**
**-7**	0.05	0.00	0.00	0.29	0.05	0.27	0.00	1.15	0.00	**1.81**
**-6**	0.05	0.00	0.00	0.00	0.11	0.26	0.33	0.00	0.00	**0.75**
**-5**	0.02	0.15	0.21	0.00	0.08	0.00	0.00	0.36	0.00	**0.83**
**-4**	0.05	0.45	0.00	0.26	0.15	0.49	0.00	0.34	0.00	**1.73**
**-3**	0.06	0.00	0.00	0.00	0.11	0.47	0.00	0.33	0.25	**1.21**
**-2**	0.05	0.00	0.00	0.00	0.13	0.90	0.56	0.32	0.00	**1.96**
**-1**	0.07	0.13	0.37	0.23	0.11	1.30	0.27	0.61	0.47	**3.57**
**0**	**0.02**	**7.85**	**40.09**	**152.60**	**0.14**	**28.44**	**166.57**	**474.22**	**1414.69**	**2284.63**
**1**	0.00	0.39	0.72	1.16	0.02	0.64	1.06	4.37	4.13	**12.49**
**2**	0.00	0.13	0.56	3.34	0.01	0.22	0.55	3.85	7.54	**16.20**
**3**	0.01	0.69	0.96	2.45	0.02	1.13	1.97	7.26	11.16	**25.63**
**4**	0.01	0.28	0.98	2.76	0.02	0.46	0.58	6.09	9.96	**21.15**
**5**	0.00	0.29	0.61	2.07	0.01	0.48	2.97	6.26	13.58	**26.26**
**6**	0.00	0.15	0.00	2.39	0.00	0.49	1.22	5.01	8.18	**17.45**
**7**	0.00	0.15	0.85	2.19	0.01	0.25	1.26	6.61	12.20	**23.53**
**8**	0.00	0.16	1.10	2.53	0.02	0.26	2.27	4.54	12.02	**22.91**

Table D2. Mean inpatient costs (Admittee Patient Care dataset from Hospital Episode Statistics) per person-quarter (2017-2018 £) - polyps positive. DC = Day Case; EL = Elective Inpatient; CT = computed tomography; Int = intermediate

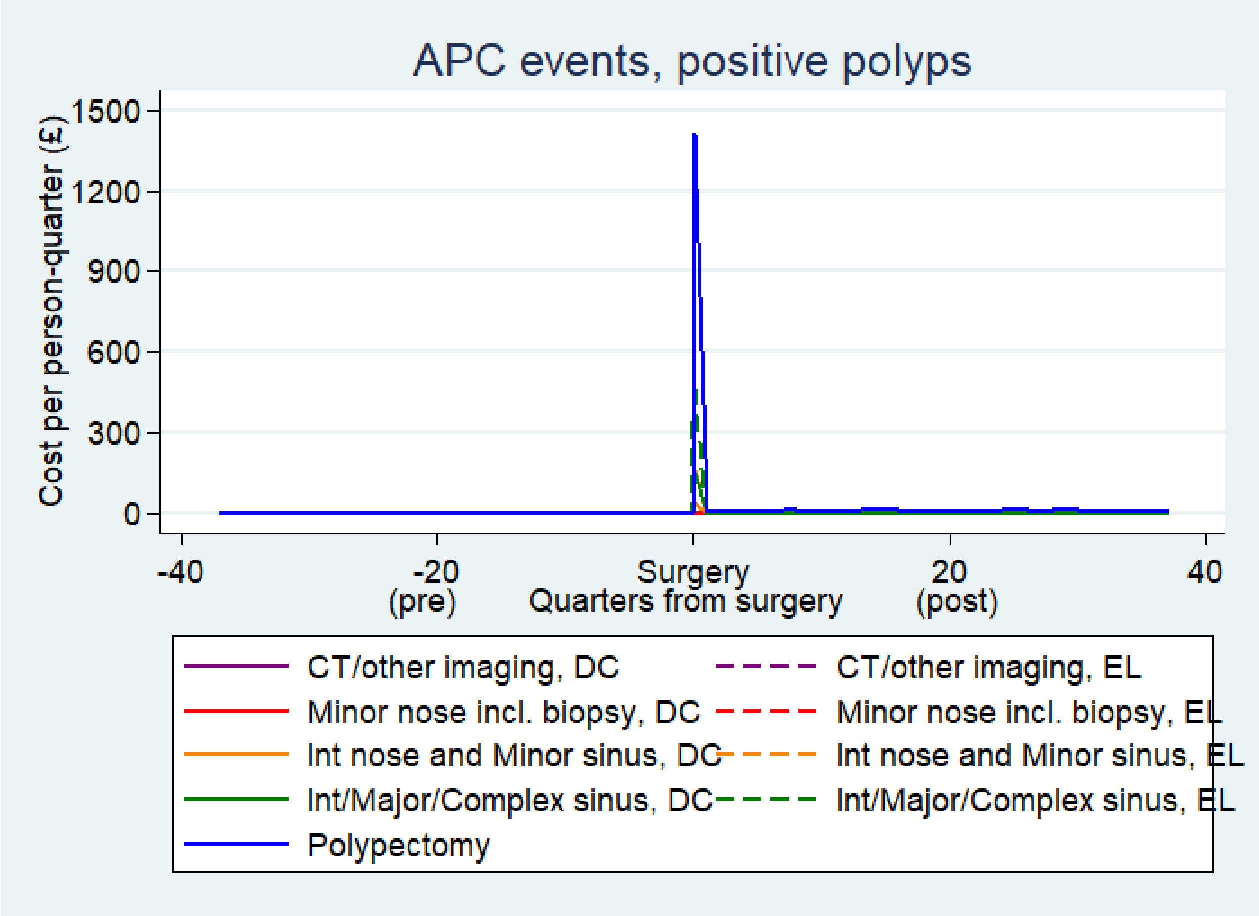
APC events, positive polyps
Cost per person-quarter (£)
1500
1200
900
600
300
0
-40
-20
(pre)
Surgery
Quarters from surgery
20
(post)
40
CT/other imaging, DC
CT/other imaging, EL
Minor nose incl. biopsy, DC
Minor nose incl. biopsy, EL
Int nose and Minor sinus, DC
Int nose and Minor sinus, EL
Int/Major/Complex sinus, DC
Int/Major/Complex sinus, EL
Polypectomy

Quarters after surgery (surgery date is at centre of Q0)	CT/other imaging, DC	Minor nose incl. biopsy, DC	Int nose and Minor sinus, DC	Int/Major/Complex sinus, DC	CT/other imaging, EL	Minor nose incl. biopsy, EL	Int nose and Minor sinus, EL	Int/Major/Complex sinus, EL	Polypectomy	TOTAL
**-8**	0.00	0.12	0.00	0.40	0.09	0.19	0.00	0.00	0.00	**0.80**
**-7**	0.04	0.00	0.00	0.19	0.06	0.18	0.00	1.28	0.00	**1.76**
**-6**	0.06	0.00	0.00	0.00	0.10	0.18	0.44	0.00	0.00	**0.77**
**-5**	0.04	0.21	0.14	0.36	0.15	0.68	0.21	0.72	0.00	**2.51**
**-4**	0.08	0.30	0.14	0.35	0.17	0.49	0.00	0.46	0.00	**1.99**
**-3**	0.05	0.00	0.13	0.00	0.15	0.63	0.00	0.44	0.17	**1.58**
**-2**	0.05	0.09	0.00	0.32	0.13	0.76	0.38	1.28	0.00	**3.02**
**-1**	0.06	0.36	0.37	0.31	0.19	1.17	0.18	1.65	0.31	**4.60**
**0**	**0.07**	**8.10**	**41.32**	**181.87**	**0.37**	**28.66**	**148.42**	**540.56**	**952.62**	**1902.00**
**1**	0.00	0.26	0.61	1.88	0.04	0.72	1.08	4.82	2.79	**12.20**
**2**	0.00	0.18	0.76	2.74	0.01	0.15	0.37	3.88	5.11	**13.20**
**3**	0.01	0.56	1.04	2.16	0.02	0.76	1.53	6.23	7.58	**19.89**
**4**	0.01	0.29	0.80	2.39	0.02	0.31	0.98	7.31	6.78	**18.89**
**5**	0.00	0.40	0.69	1.94	0.02	0.32	2.23	4.71	9.27	**19.58**
**6**	0.00	0.10	0.14	2.18	0.01	0.50	1.05	4.61	5.59	**14.18**
**7**	0.00	0.11	1.17	2.24	0.02	0.35	1.08	6.25	8.37	**19.58**
**8**	0.00	0.11	0.91	2.12	0.03	0.36	1.78	3.87	8.26	**17.43**

Table D3. Mean inpatient costs (Admittee Patient Care dataset from Hospital Episode Statistics) per person-quarter (2017-2018 £) – all polyp statuses. DC = Day Case; EL = Elective Inpatient; CT = computed tomography; Int = intermediate

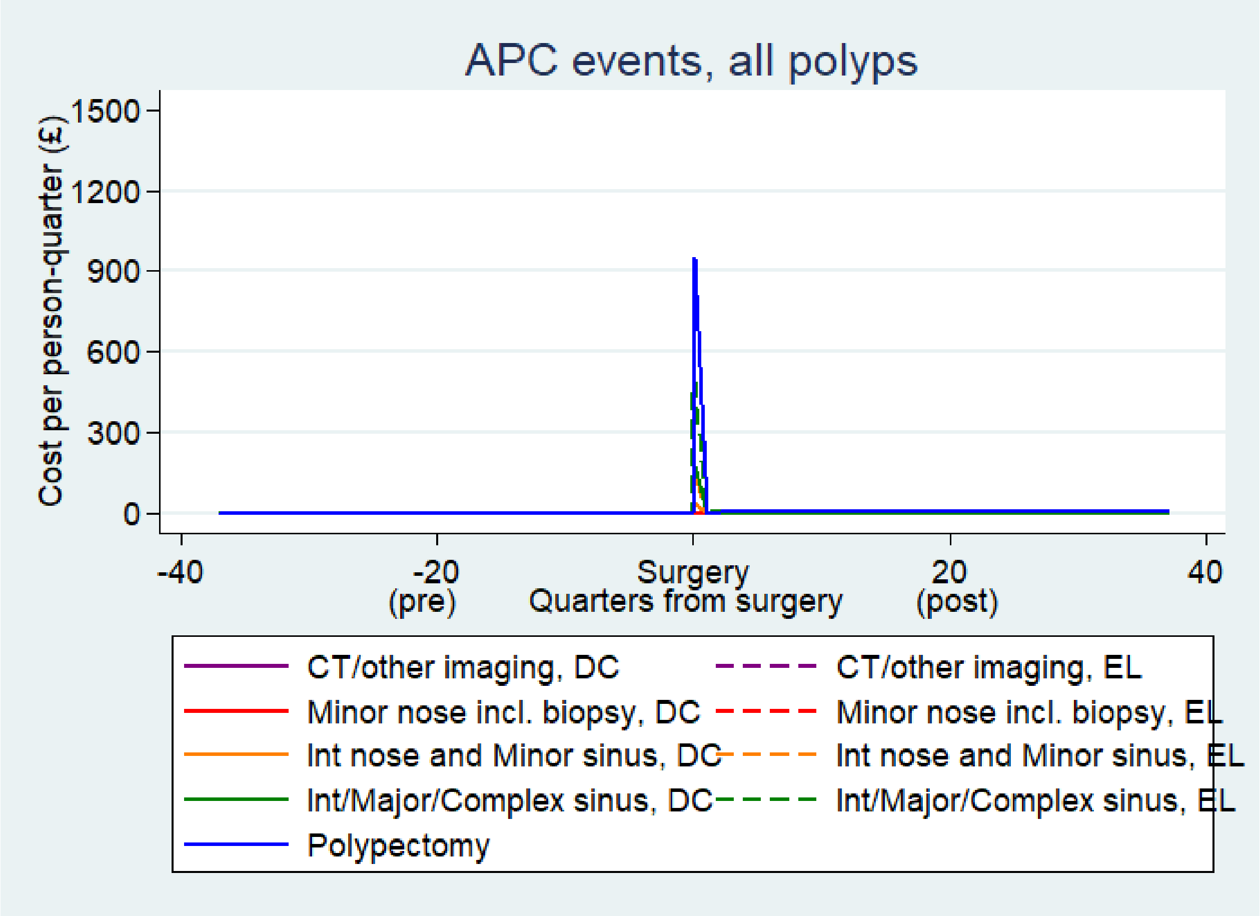
APC events, all polyps
Cost per person-quarter (£)
1500
1200
900
600
300
0
-40
-20
(pre)
Surgery
Quarters from surgery
20
(post)
40
CT/other imaging, DC
CT/other imaging, EL
Minor nose incl. biopsy, DC
Minor nose incl. biopsy, EL
Int nose and Minor sinus, DC
Int nose and Minor sinus, EL
Int/Major/Complex sinus, DC
Int/Major/Complex sinus, EL
Polypectomy

E. Section E – Mean outpatient costs per person-quarter, centred on surgery date at midpoint of Q0

**Quarters after surgery (surgery date is at centre of Q0)**	CT/other imaging	Minor nose incl. biopsy	Polypectomy	Int nose and Minor sinus	Int sinus	Major/ complex sinus	**TOTAL**
**-8**	8.52	0.80	0.08	3.53	3.79	2.98	**19.69**
**-7**	10.60	1.00	0.03	3.84	5.26	3.43	**24.15**
**-6**	8.69	1.14	0.10	4.01	5.15	1.65	**20.74**
**-5**	9.70	1.03	0.05	4.66	5.60	3.16	**24.21**
**-4**	10.23	0.99	0.16	5.16	6.85	1.51	**24.90**
**-3**	13.62	1.22	0.18	6.06	6.81	3.37	**31.25**
**-2**	16.24	1.64	0.08	7.58	8.27	1.84	**35.67**
**-1**	25.04	1.84	0.44	11.36	12.02	6.17	**56.87**
**0**	**23.03**	**2.93**	**0.85**	**12.47**	**14.46**	**8.66**	**62.41**
**1**	14.88	1.63	0.32	7.57	8.64	2.97	**36.00**
**2**	12.05	1.86	0.12	6.30	6.76	2.04	**29.12**
**3**	9.76	1.53	0.17	6.18	5.75	2.09	**25.48**
**4**	9.36	1.18	0.18	5.40	5.83	2.51	**24.46**
**5**	9.66	0.97	0.15	4.94	5.13	2.58	**23.43**
**6**	9.66	1.18	0.12	4.56	5.95	3.04	**24.50**
**7**	8.91	1.23	0.27	3.90	5.41	1.56	**21.28**
**8**	8.76	1.12	0.13	3.86	5.01	1.61	**20.49**

Table E1. Mean outpatient costs (Outpatient Care dataset from Hospital Episode Statistics) per person-quarter (£) - polyps positive.

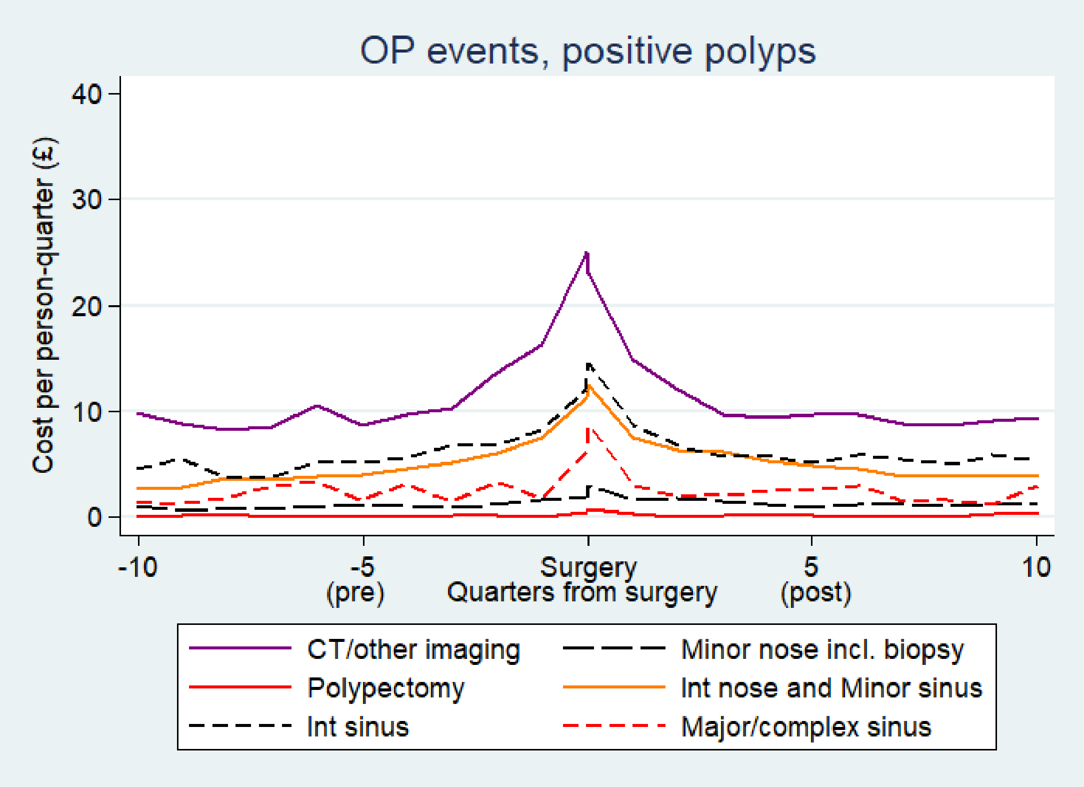

Clarke CS, et al. BMJ Open 2022; 12:e055603. doi: 10.1136/bmjopen-2021-055603

Quarters after surgery (surgery date is at centre of Q0)	CT/other imaging	Minor nose incl. biopsy	Polypectomy	Int nose and Minor sinus	Int sinus	Major/ complex sinus	TOTAL
**-8**	13.55	2.39	-	5.19	7.41	1.13	**29.67**
**-7**	15.59	3.31	-	4.24	7.94	0.00	**31.07**
**-6**	16.30	2.64	-	5.10	7.48	1.04	**32.57**
**-5**	16.35	2.34	-	5.21	7.07	0.00	**30.97**
**-4**	19.16	2.26	-	5.80	9.08	5.83	**42.14**
**-3**	20.40	2.40	-	7.48	9.41	3.72	**43.42**
**-2**	25.25	2.51	-	9.04	11.10	0.90	**48.79**
**-1**	32.30	4.55	-	11.26	14.70	5.18	**67.99**
**0**	**29.11**	**4.37**	**-**	**13.95**	**17.65**	**10.59**	**75.68**
**1**	25.51	3.16	-	12.17	12.35	5.51	**58.70**
**2**	17.85	2.54	-	9.62	9.16	1.43	**40.59**
**3**	15.27	2.41	-	7.26	8.76	2.22	**35.91**
**4**	15.19	1.93	-	6.57	7.83	2.29	**33.81**
**5**	15.00	2.01	-	6.24	7.46	1.59	**32.30**
**6**	13.02	1.57	-	6.59	6.54	4.92	**32.65**
**7**	14.07	1.32	-	5.68	5.89	9.38	**36.33**
**8**	15.98	1.28	-	5.63	5.87	2.65	**31.40**

Table E2. Mean outpatient costs (Outpatient Care dataset from Hospital Episode Statistics) per person-quarter (£) - polyps unknown.

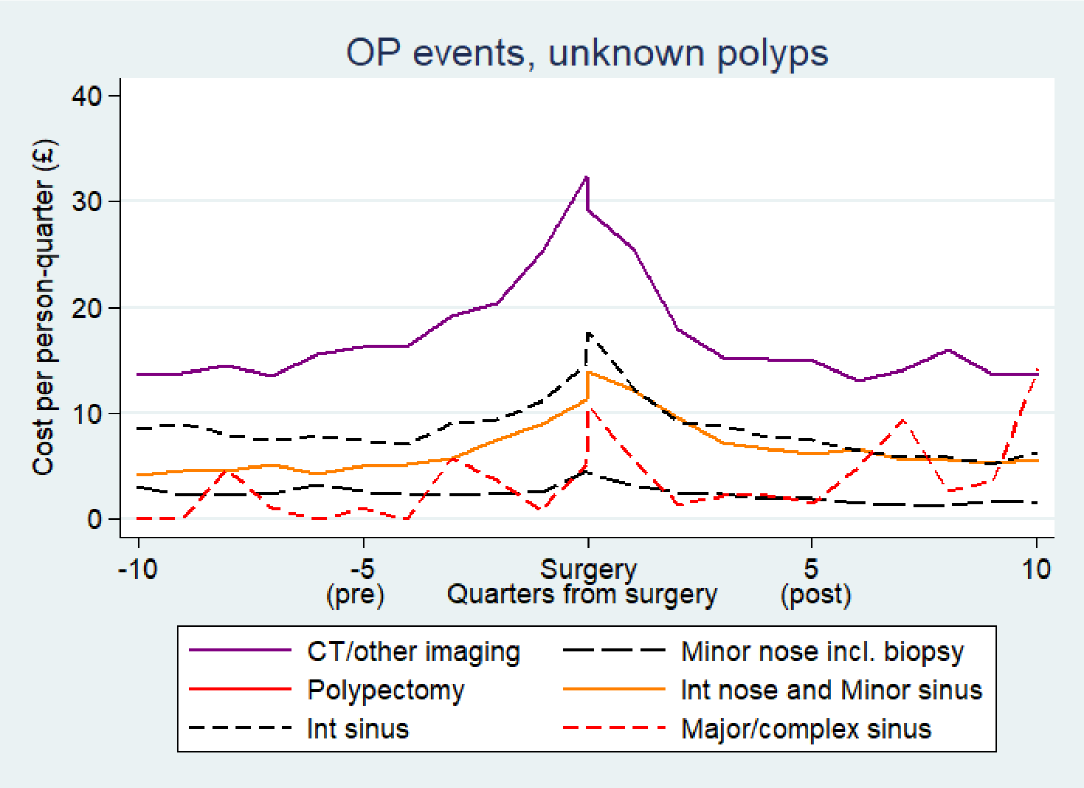

16

**Quarters after surgery (surgery date is at centre of Q0)**	CT/other imaging	Minor nose incl. biopsy	Polypectomy	Int nose and Minor sinus	Int sinus	Major/ complex sinus	**TOTAL**
**-8**	10.25	1.35	0.05	4.10	5.04	2.34	**23.14**
**-7**	12.32	1.80	0.02	3.98	6.18	2.25	**26.53**
**-6**	11.32	1.66	0.07	4.39	5.95	1.44	**24.82**
**-5**	11.98	1.48	0.03	4.85	6.10	2.08	**26.53**
**-4**	13.28	1.42	0.11	5.38	7.61	2.99	**30.78**
**-3**	15.93	1.62	0.12	6.54	7.70	3.49	**35.40**
**-2**	19.29	1.94	0.06	8.07	9.23	1.52	**40.11**
**-1**	27.49	2.76	0.29	11.33	12.93	5.83	**60.62**
**0**	**25.01**	**3.40**	**0.57**	**12.96**	**15.51**	**9.29**	**66.75**
**1**	18.32	2.13	0.21	9.06	9.84	3.79	**43.35**
**2**	13.92	2.08	0.08	7.37	7.53	1.84	**32.82**
**3**	11.52	1.81	0.12	6.52	6.71	2.13	**28.82**
**4**	11.22	1.42	0.12	5.77	6.47	2.44	**27.45**
**5**	11.35	1.30	0.10	5.35	5.87	2.27	**26.24**
**6**	10.72	1.30	0.08	5.21	6.14	3.63	**27.08**
**7**	10.53	1.26	0.18	4.46	5.56	4.01	**26.00**
**8**	11.02	1.17	0.09	4.41	5.28	1.93	**23.91**

Table E3. Mean outpatient costs (Outpatient Care dataset from Hospital Episode Statistics) per person-quarter (£) – all patients.

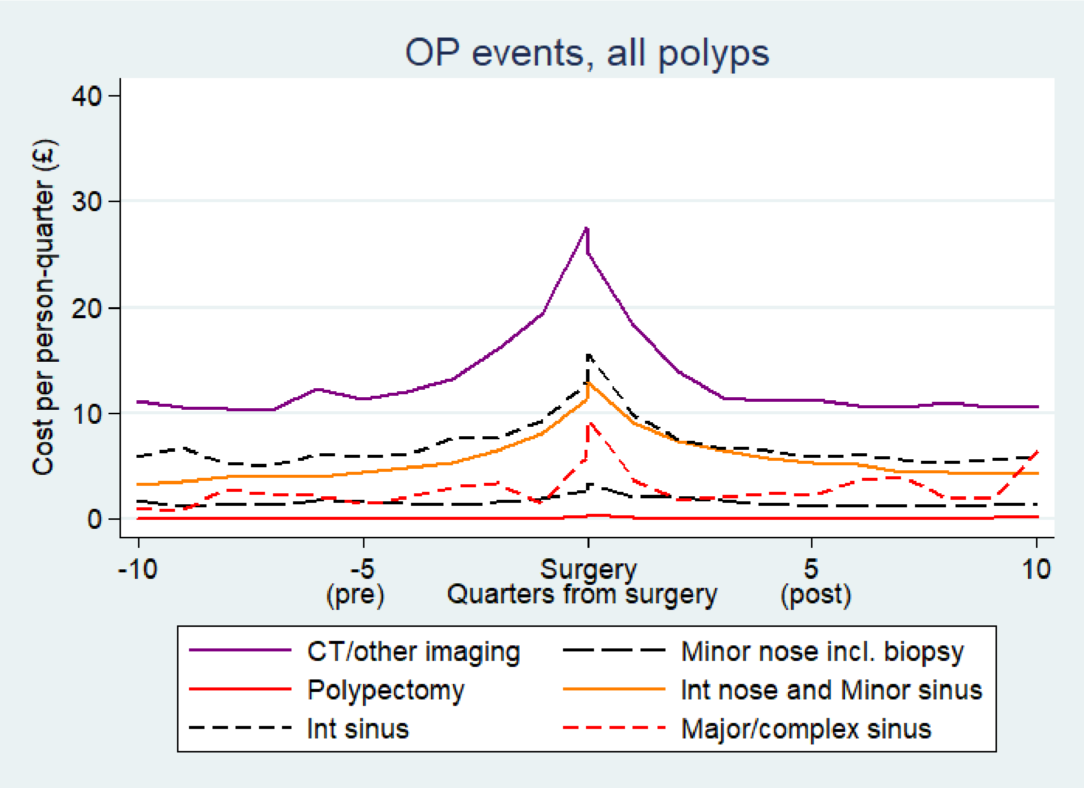

Clarke CS, *et al. BMJ Open* 2022; 12:e055603. doi: 10.1136/bmjopen-2021-055603

F. Section F – Mean primary care consultation costs per person-quarter, centred on surgery date at midpoint of Q0

**Quarters after surgery (surgery date is at centre of Q0)**	GP consultati on	GP home visit	GP telephone	Nurse consultati on	Nurse home visit	Nurse telephone	**TOTAL**
**-8**	7.98	0.03	0.09	0.09	0.01	0.00	**8.20**
**-7**	8.88	0.04	0.10	0.09	0.01	0.00	**9.12**
**-6**	10.45	0.06	0.17	0.13	0.00	0.00	**10.82**
**-5**	12.93	0.04	0.19	0.15	0.01	0.01	**13.31**
**-4**	16.95	0.06	0.21	0.17	0.01	0.01	**17.41**
**-3**	22.30	0.11	0.27	0.26	0.01	0.01	**22.96**
**-2**	27.36	0.11	0.39	0.33	0.02	0.01	**28.23**
**-1**	22.53	0.10	0.44	0.33	0.03	0.01	**23.43**
**0**	**5.36**	**0.01**	**0.12**	**0.09**	**0.01**	**0.00**	**5.59**
**1**	4.63	0.02	0.11	0.07	0.00	0.01	**4.85**
**2**	4.83	0.05	0.13	0.06	0.01	0.00	**5.09**
**3**	4.47	0.01	0.09	0.07	0.01	0.00	**4.65**
**4**	4.43	0.05	0.11	0.07	0.01	0.00	**4.67**
**5**	4.45	0.02	0.13	0.11	0.01	0.00	**4.72**
**6**	4.36	0.04	0.14	0.07	0.01	0.01	**4.62**
**7**	4.32	0.03	0.13	0.06	0.00	0.00	**4.55**
**8**	4.51	0.01	0.14	0.06	0.01	0.00	**4.72**

Table F1. Mean primary care consultation costs (CPRD dataset) per person-quarter (£) - polyps positive.

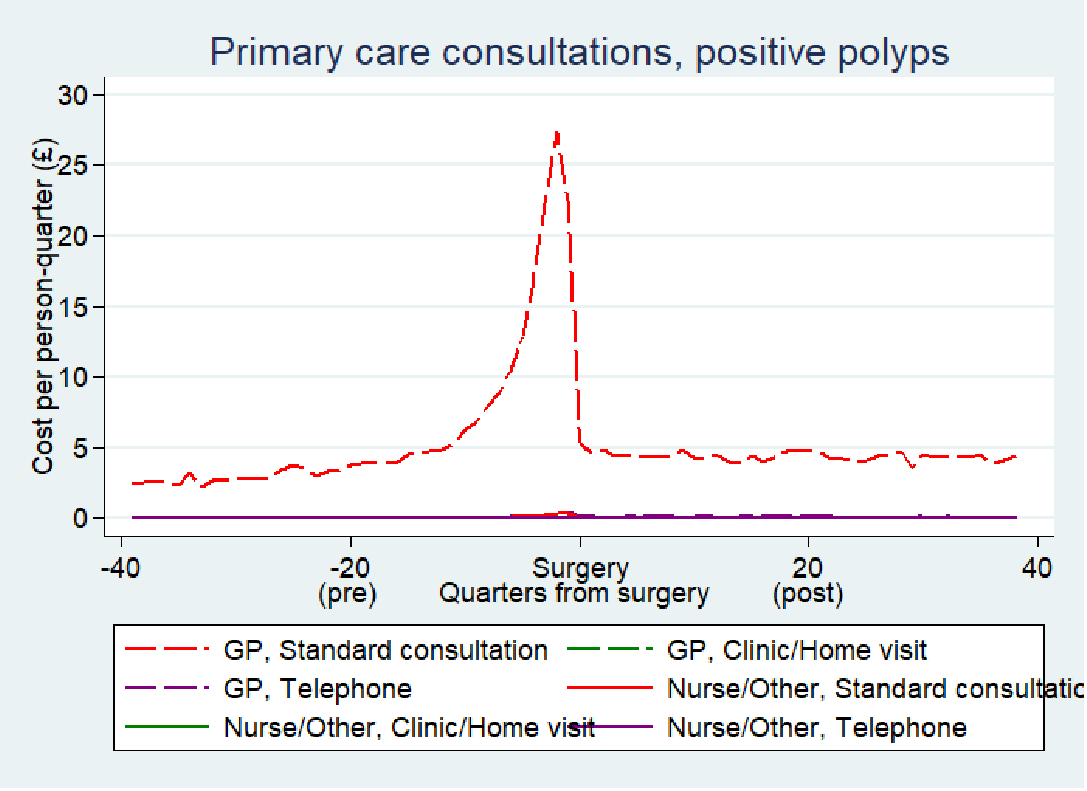

Quarters after surgery (surgery date is at centre of Q0)	GP consultation	GP home visit	GP telephone	Nurse consultation	Nurse home visit	Nurse telephone	TOTAL
**-8**	8.43	0.02	0.11	0.10	0.01	0.00	**8.68**
**-7**	9.64	0.02	0.14	0.13	0.01	0.00	**9.95**
**-6**	12.32	0.03	0.22	0.13	0.01	0.01	**12.72**
**-5**	14.46	0.03	0.24	0.14	0.01	0.00	**14.89**
**-4**	19.41	0.06	0.31	0.22	0.02	0.01	**20.04**
**-3**	21.60	0.08	0.51	0.28	0.02	0.01	**22.50**
**-2**	21.82	0.07	0.50	0.33	0.02	0.01	**22.76**
**-1**	16.23	0.08	0.52	0.25	0.01	0.01	**17.11**
**0**	**6.69**	**0.01**	**0.22**	**0.11**	**0.01**	**0.00**	**7.04**
**1**	6.77	0.00	0.22	0.12	0.01	0.01	**7.13**
**2**	6.74	0.02	0.09	0.07	0.01	0.01	**6.94**
**3**	5.47	0.02	0.08	0.08	0.00	0.00	**5.66**
**4**	6.57	0.02	0.15	0.07	0.00	0.00	**6.82**
**5**	6.38	0.05	0.12	0.07	0.01	0.00	**6.63**
**6**	5.03	0.03	0.16	0.07	0.01	0.00	**5.31**
**7**	5.85	0.02	0.17	0.05	0.00	0.00	**6.10**
**8**	6.56	0.02	0.17	0.12	0.00	0.00	**6.88**

Table F2. Mean primary care consultation costs (CPRD dataset) per person-quarter (£) - polyps unknown.

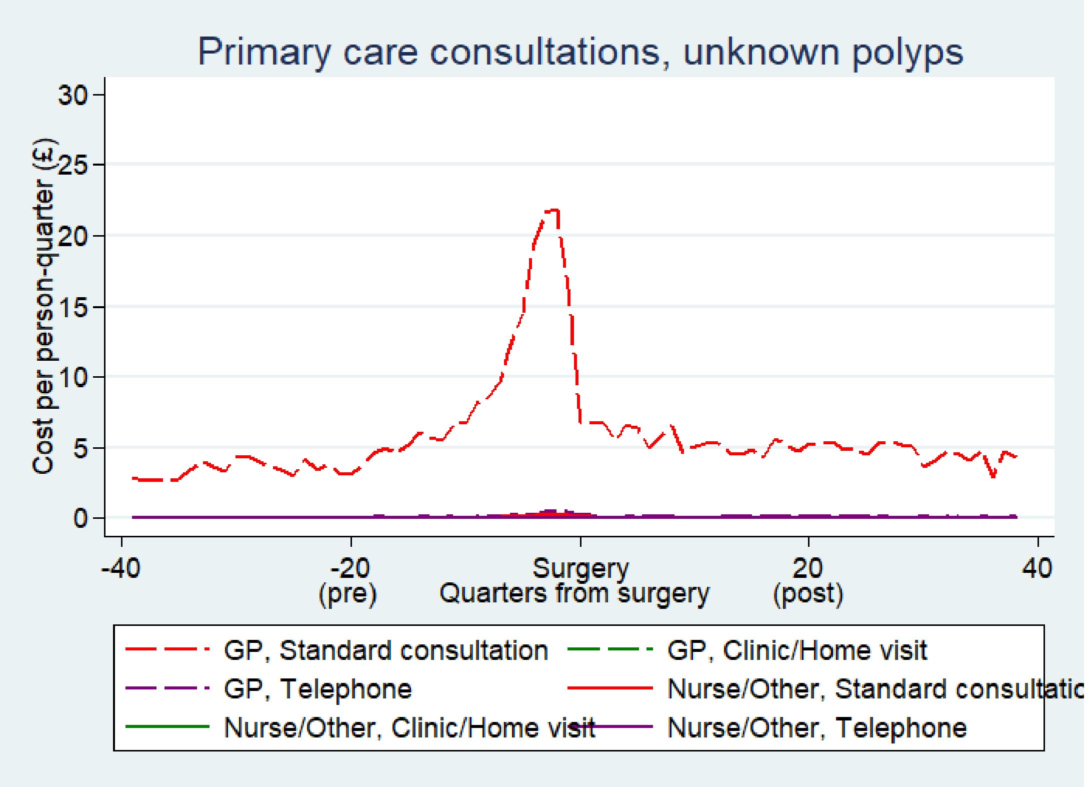

Clarke CS, et al. BMJ Open 2022; 12:e055603. doi: 10.1136/bmjopen-2021-055603

**Quarters after surgery (surgery date is at centre of Q0)**	GP consultation	GP home visit	GP telephone	Nurse consultation	Nurse home visit	Nurse telephone	**TOTAL**
**-8**	8.13	0.03	0.10	0.09	0.01	0.00	**8.36**
**-7**	9.13	0.03	0.11	0.10	0.01	0.00	**9.39**
**-6**	11.06	0.05	0.19	0.13	0.01	0.00	**11.45**
**-5**	13.43	0.03	0.20	0.15	0.01	0.01	**13.83**
**-4**	17.75	0.06	0.25	0.19	0.01	0.01	**18.27**
**-3**	22.07	0.10	0.35	0.27	0.02	0.01	**22.81**
**-2**	25.56	0.10	0.42	0.33	0.02	0.01	**26.44**
**-1**	20.47	0.09	0.46	0.31	0.02	0.01	**21.37**
**0**	**5.79**	**0.01**	**0.15**	**0.10**	**0.01**	**0.00**	**6.06**
**1**	5.32	0.01	0.15	0.09	0.01	0.01	**5.58**
**2**	5.44	0.04	0.12	0.07	0.01	0.00	**5.68**
**3**	4.79	0.02	0.09	0.07	0.01	0.00	**4.97**
**4**	5.11	0.04	0.12	0.07	0.00	0.00	**5.35**
**5**	5.06	0.03	0.13	0.10	0.01	0.00	**5.32**
**6**	4.57	0.03	0.14	0.07	0.01	0.01	**4.84**
**7**	4.80	0.03	0.14	0.06	0.00	0.00	**5.04**
**8**	5.15	0.01	0.15	0.07	0.01	0.00	**5.40**

Table F3. Mean primary care consultation costs (CPRD dataset) per person-quarter (£) – all patients.

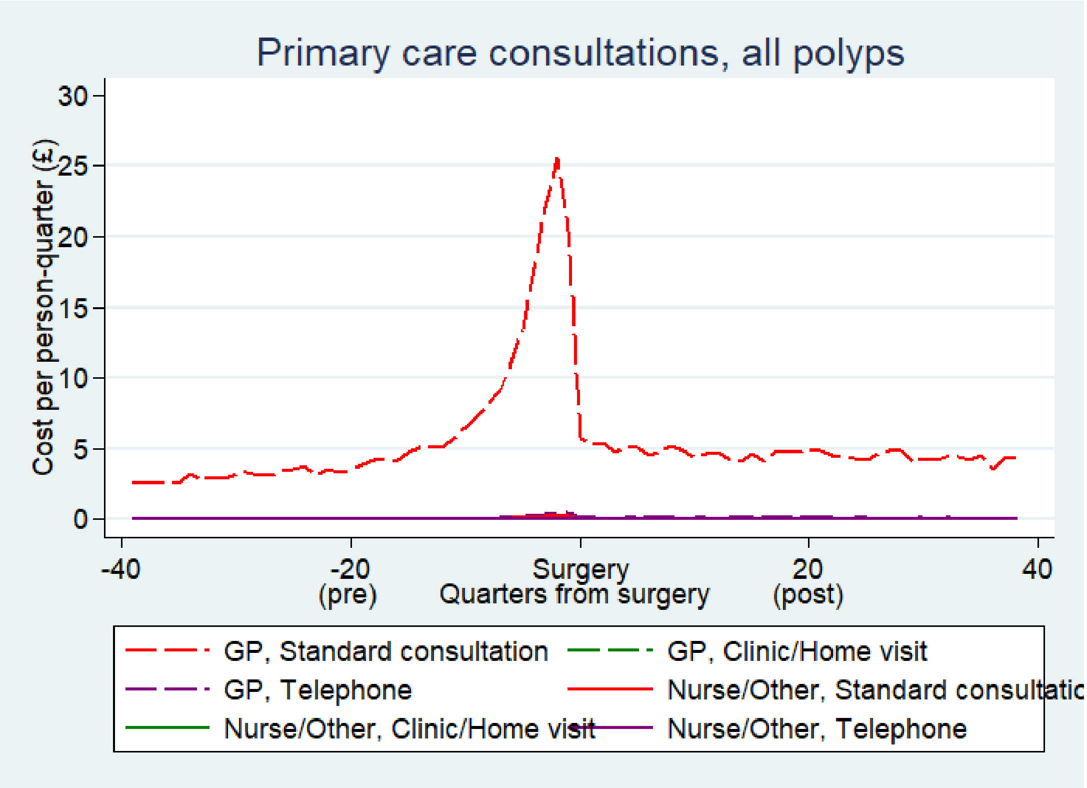

Clarke CS, et al. BMJ Open 2022; 12:e055603. doi: 10.1136/bmjopen-2021-055603

G. Section G – Mean primary care antibiotic prescription costs per-person-quarter, centred on surgery date at midpoint of Q0

Quarters after surgery (surgery date at centre of Q0)	Cephalo-sporin	Macrolides	Metro-nidazole	Penicillin	Quinolone	Tetra-cycline	Other	TOTAL
**-8**	0.07	0.26	0.01	0.18	0.02	0.30	0.03	**0.87**
**-7**	0.08	0.28	0.01	0.18	0.03	0.34	0.04	**0.97**
**-6**	0.08	0.27	0.01	0.20	0.02	0.36	0.03	**0.98**
**-5**	0.09	0.29	0.01	0.21	0.02	0.42	0.04	**1.08**
**-4**	0.09	0.33	0.01	0.22	0.03	0.44	0.04	**1.15**
**-3**	0.09	0.39	0.01	0.24	0.03	0.57	0.05	**1.37**
**-2**	0.10	0.41	0.01	0.25	0.03	0.63	0.04	**1.46**
**-1**	0.07	0.41	0.01	0.24	0.03	0.51	0.04	**1.32**
**0**	**0.09**	**0.31**	**0.01**	**0.21**	**0.03**	**0.31**	**0.04**	**0.99**
**1**	0.07	0.29	0.01	0.19	0.03	0.28	0.04	**0.92**
**2**	0.07	0.29	0.01	0.20	0.03	0.30	0.04	**0.93**
**3**	0.08	0.26	0.01	0.20	0.03	0.32	0.04	**0.95**
**4**	0.08	0.30	0.01	0.19	0.03	0.32	0.05	**0.97**
**5**	0.07	0.25	0.01	0.20	0.03	0.33	0.04	**0.93**
**6**	0.09	0.29	0.01	0.20	0.03	0.31	0.05	**0.97**
**7**	0.08	0.30	0.01	0.19	0.03	0.30	0.04	**0.94**
**8**	0.08	0.28	0.01	0.20	0.03	0.35	0.04	**0.99**

Table G1. Mean primary care antibiotic prescription costs (CPRD dataset) per person-quarter (£) - polyps positive.

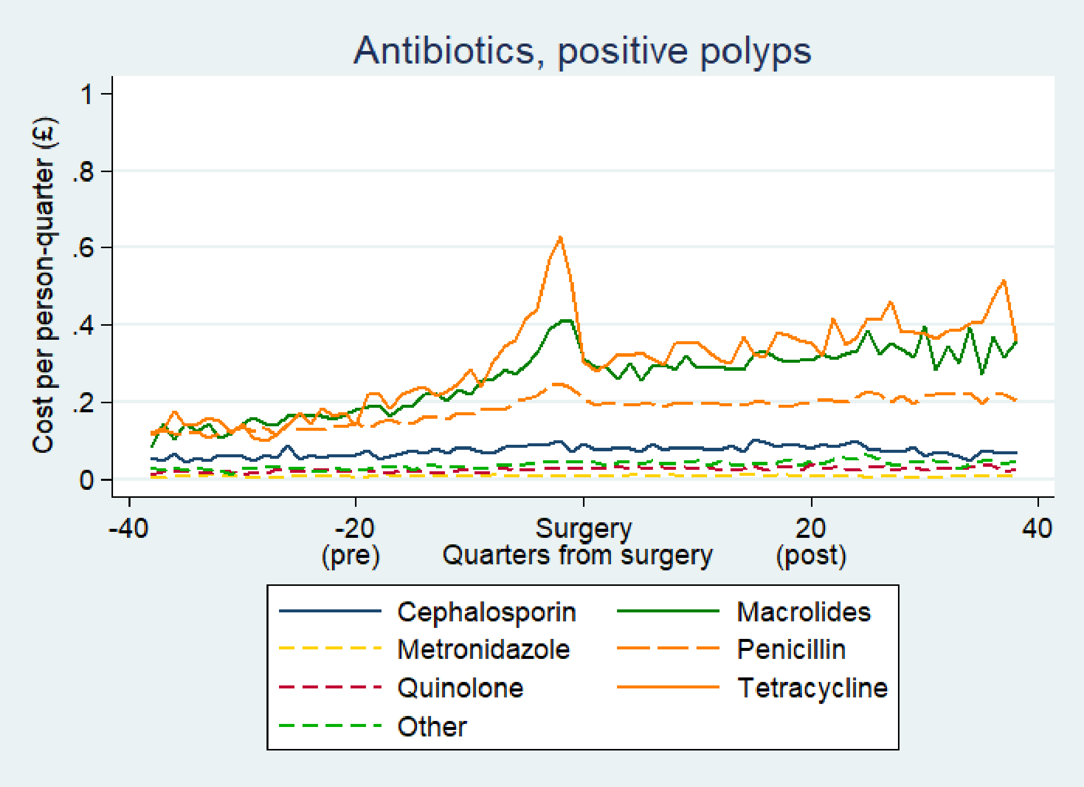

Clarke CS, *et al. BMJ Open* 2022; 12:e055603. doi: 10.1136/bmjopen-2021-055603

Quarters after surgery (surgery date at centre of Q0)	Cephalo-sporin	Macrolides	Metro-nidazole	Penicillin	Quinolone	Tetra-cycline	Other	TOTAL
**-8**	0.11	0.29	0.02	0.19	0.02	0.52	0.06	**1.21**
**-7**	0.11	0.32	0.01	0.22	0.02	0.52	0.04	**1.24**
**-6**	0.11	0.43	0.01	0.23	0.03	0.64	0.03	**1.49**
**-5**	0.15	0.42	0.01	0.28	0.03	0.65	0.05	**1.59**
**-4**	0.14	0.52	0.01	0.29	0.04	0.90	0.05	**1.96**
**-3**	0.13	0.62	0.02	0.30	0.04	0.98	0.04	**2.15**
**-2**	0.13	0.60	0.02	0.29	0.04	0.98	0.05	**2.11**
**-1**	0.13	0.54	0.01	0.27	0.03	0.77	0.05	**1.82**
**0**	**0.13**	**0.37**	**0.01**	**0.21**	**0.04**	**0.45**	**0.05**	**1.27**
**1**	0.11	0.35	0.01	0.22	0.04	0.50	0.05	**1.29**
**2**	0.10	0.36	0.02	0.20	0.04	0.50	0.05	**1.26**
**3**	0.10	0.32	0.01	0.19	0.03	0.48	0.04	**1.17**
**4**	0.10	0.35	0.01	0.21	0.03	0.54	0.06	**1.30**
**5**	0.10	0.38	0.01	0.20	0.03	0.47	0.05	**1.24**
**6**	0.08	0.39	0.01	0.18	0.03	0.48	0.05	**1.23**
**7**	0.09	0.36	0.01	0.20	0.03	0.51	0.06	**1.26**
**8**	0.10	0.40	0.02	0.19	0.03	0.52	0.05	**1.30**

Table G2. Mean primary care antibiotic prescription costs (CPRD dataset) per person-quarter (£) - polyps unknown.

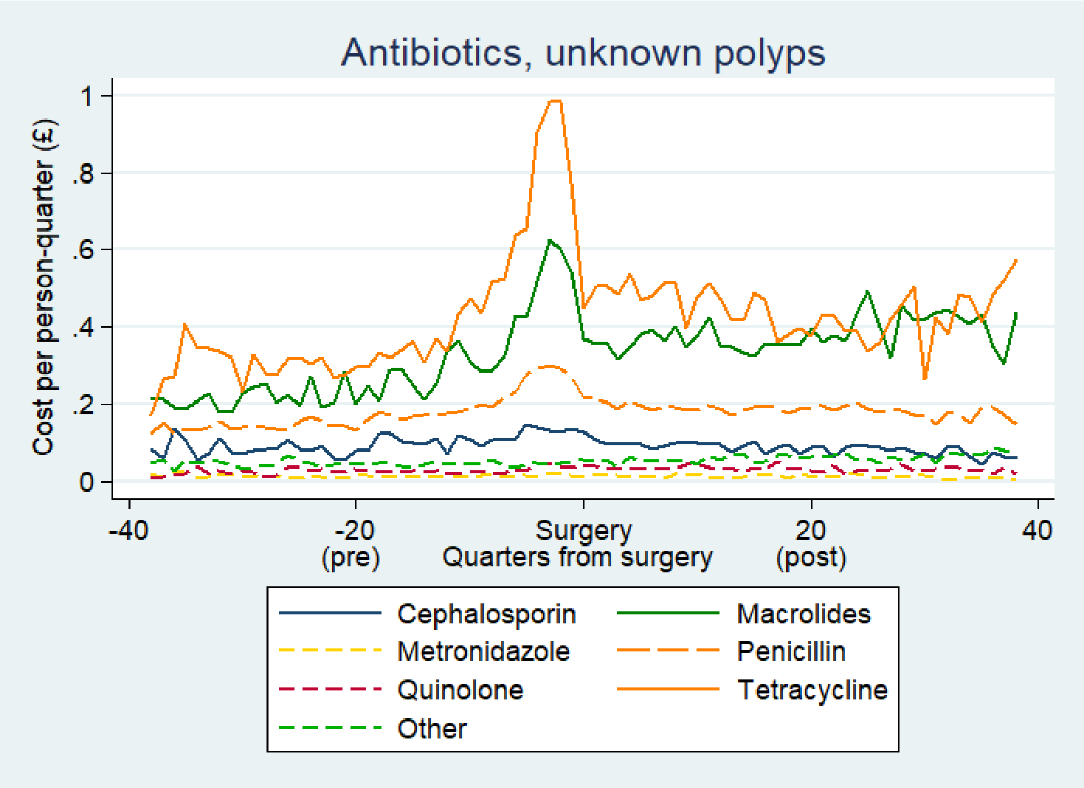

Clarke CS, et al. BMJ Open 2022; 12:e055603. doi: 10.1136/bmjopen-2021-055603

**Quarters after surgery (surgery date at centre of Q0)**	Cephalo-sporin	Macrolides	Metro-nidazole	Penicillin	Quinolone	Tetra-cycline	Other	**TOTAL**
**-8**	0.08	0.27	0.01	0.19	0.02	0.37	0.04	**0.98**
**-7**	0.09	0.30	0.01	0.19	0.03	0.40	0.04	**1.06**
**-6**	0.09	0.32	0.01	0.21	0.03	0.45	0.03	**1.15**
**-5**	0.11	0.34	0.01	0.23	0.02	0.50	0.04	**1.25**
**-4**	0.11	0.39	0.01	0.24	0.03	0.59	0.04	**1.41**
**-3**	0.10	0.47	0.01	0.26	0.03	0.71	0.04	**1.62**
**-2**	0.11	0.47	0.01	0.26	0.03	0.74	0.05	**1.68**
**-1**	0.09	0.46	0.01	0.25	0.03	0.60	0.05	**1.48**
**0**	**0.10**	**0.33**	**0.01**	**0.21**	**0.03**	**0.35**	**0.05**	**1.08**
**1**	0.08	0.31	0.01	0.20	0.03	0.35	0.05	**1.04**
**2**	0.08	0.31	0.01	0.20	0.03	0.36	0.04	**1.04**
**3**	0.09	0.28	0.01	0.19	0.03	0.37	0.04	**1.02**
**4**	0.08	0.32	0.01	0.20	0.03	0.39	0.05	**1.08**
**5**	0.08	0.29	0.01	0.20	0.03	0.37	0.04	**1.03**
**6**	0.09	0.32	0.01	0.19	0.03	0.37	0.05	**1.05**
**7**	0.08	0.32	0.01	0.19	0.03	0.37	0.04	**1.04**
**8**	0.09	0.32	0.01	0.20	0.03	0.40	0.04	**1.09**

Table G3. Mean primary care antibiotic prescription costs (CPRD dataset) per person-quarter (£) – all patients.

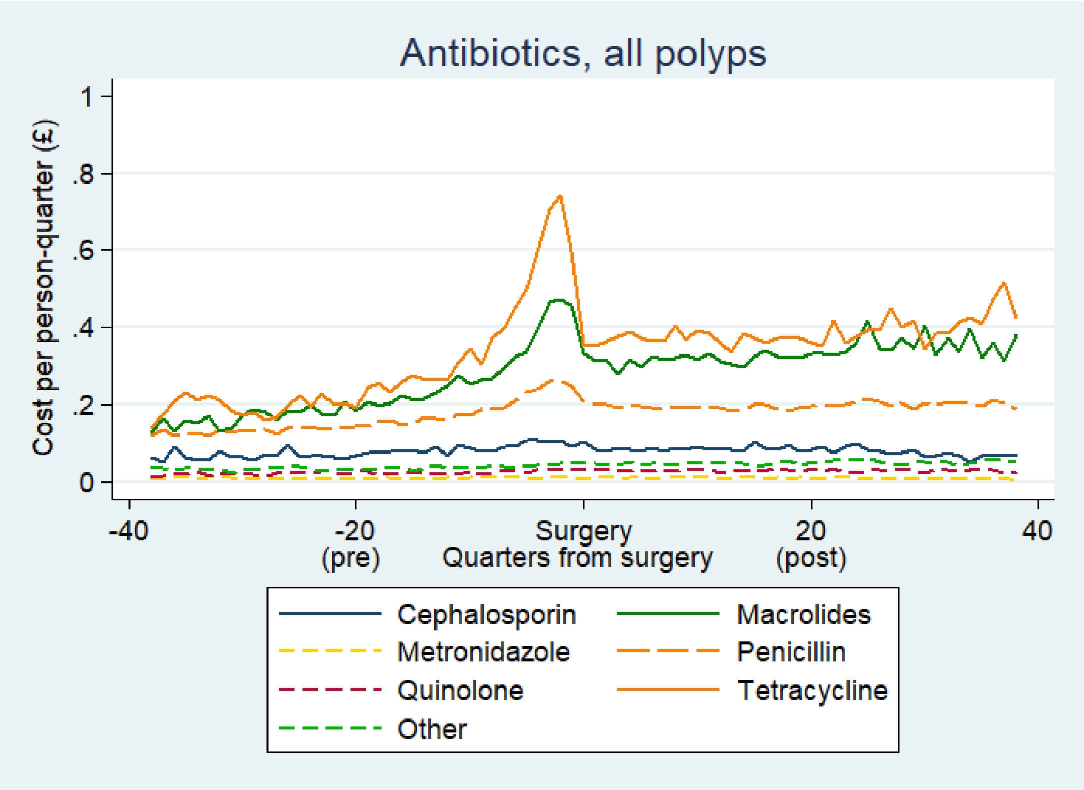

H. Section H – Mean primary care non-antibiotic prescription costs per-person-quarter, centred on surgery date at midpoint of Q0

Quarters after surgery (surgery date at centre of Q0)	Steroids	All other non-abx meds	TOTAL
**-8**	3.80	2.17	**5.97**
**-7**	3.96	2.24	**6.20**
**-6**	4.35	2.25	**6.60**
**-5**	4.89	2.37	**7.26**
**-4**	5.45	2.43	**7.88**
**-3**	6.12	2.56	**8.68**
**-2**	6.84	2.57	**9.41**
**-1**	6.48	2.64	**9.13**
**0**	**5.33**	**2.46**	**7.79**
**1**	5.15	2.51	**7.66**
**2**	5.12	2.60	**7.72**
**3**	4.90	2.69	**7.59**
**4**	4.81	2.67	**7.49**
**5**	4.86	2.76	**7.62**
**6**	4.90	2.82	**7.73**
**7**	4.83	2.82	**7.65**
**8**	4.83	2.79	**7.62**

Table H1. Mean primary care non-antibiotic prescription costs (CPRD dataset) per person-quarter (£) - polyps positive.

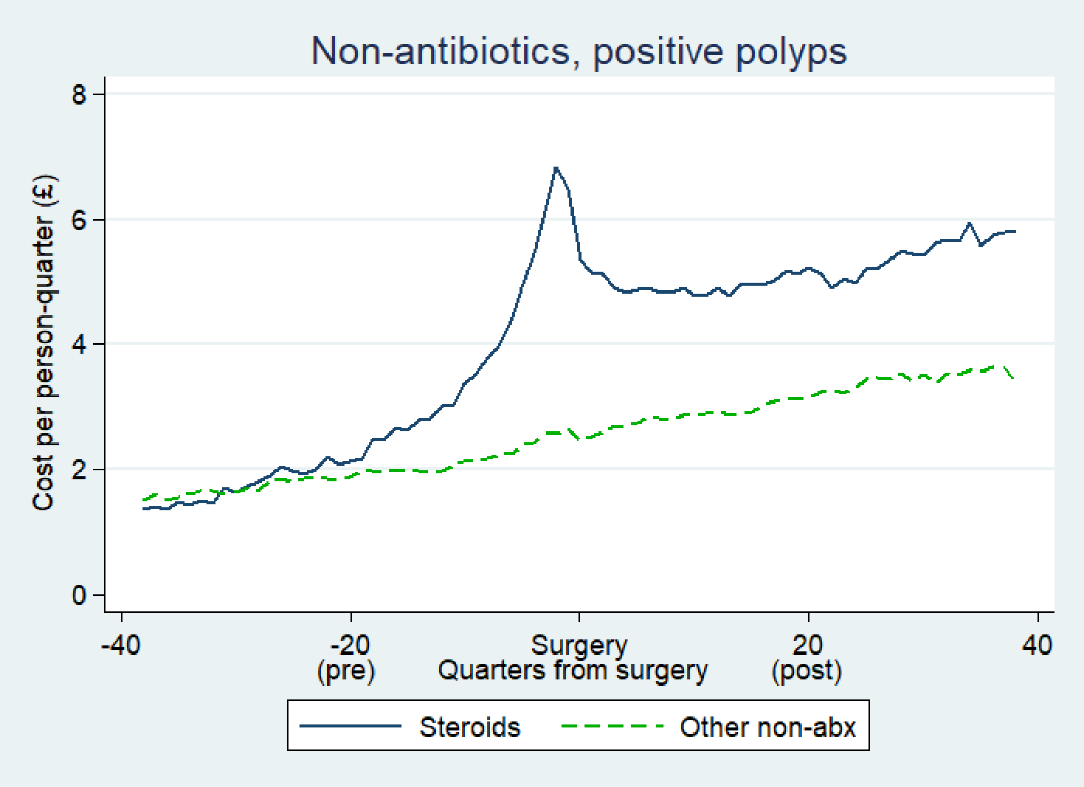

Quarters after surgery (surgery date at centre of Q0)	Steroids	All other non-abx meds	TOTAL
**-8**	1.96	2.75	**4.70**
**-7**	2.09	2.81	**4.89**
**-6**	2.43	2.82	**5.25**
**-5**	2.76	2.92	**5.68**
**-4**	3.22	3.03	**6.25**
**-3**	3.61	3.03	**6.64**
**-2**	3.76	3.19	**6.96**
**-1**	3.43	3.13	**6.56**
**0**	**2.62**	**2.93**	**5.54**
**1**	2.63	3.00	**5.63**
**2**	2.47	3.02	**5.49**
**3**	2.34	3.04	**5.39**
**4**	2.50	3.04	**5.54**
**5**	2.43	3.12	**5.55**
**6**	2.40	3.05	**5.45**
**7**	2.31	3.05	**5.36**
**8**	2.38	3.18	**5.56**

Table H2. Mean primary care non-antibiotic prescription costs (CPRD dataset) per person-quarter (£) - polyps unknown.

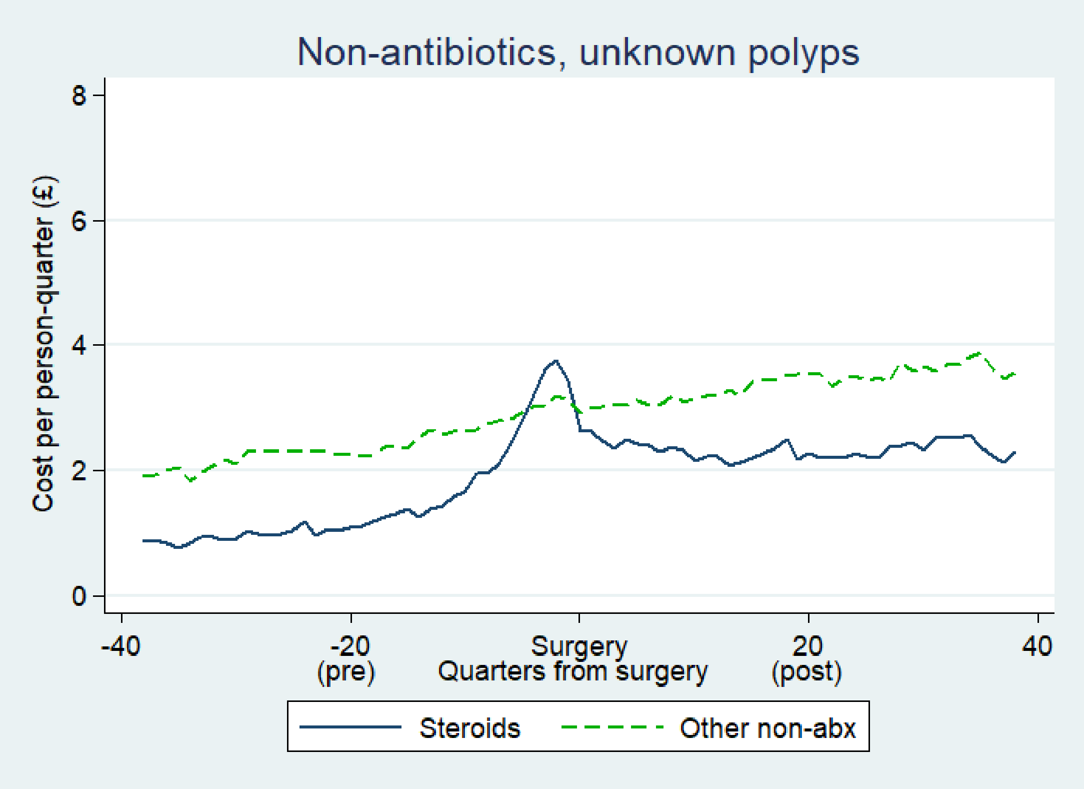

**Quarters after surgery (surgery date at centre of Q0)**	Steroids	All other non-abx meds	**TOTAL**
**-8**	3.19	2.36	**5.55**
**-7**	3.34	2.42	**5.77**
**-6**	3.72	2.44	**6.15**
**-5**	4.19	2.55	**6.74**
**-4**	4.72	2.63	**7.35**
**-3**	5.30	2.71	**8.01**
**-2**	5.84	2.77	**8.61**
**-1**	5.49	2.80	**8.29**
**0**	**4.45**	**2.61**	**7.06**
**1**	4.34	2.67	**7.00**
**2**	4.27	2.73	**7.00**
**3**	4.09	2.80	**6.89**
**4**	4.08	2.79	**6.87**
**5**	4.09	2.87	**6.96**
**6**	4.12	2.89	**7.01**
**7**	4.04	2.89	**6.93**
**8**	4.07	2.91	**6.98**

Table H3. Mean primary care non-antibiotic prescription costs (CPRD dataset) per person-quarter (£) – all patients.

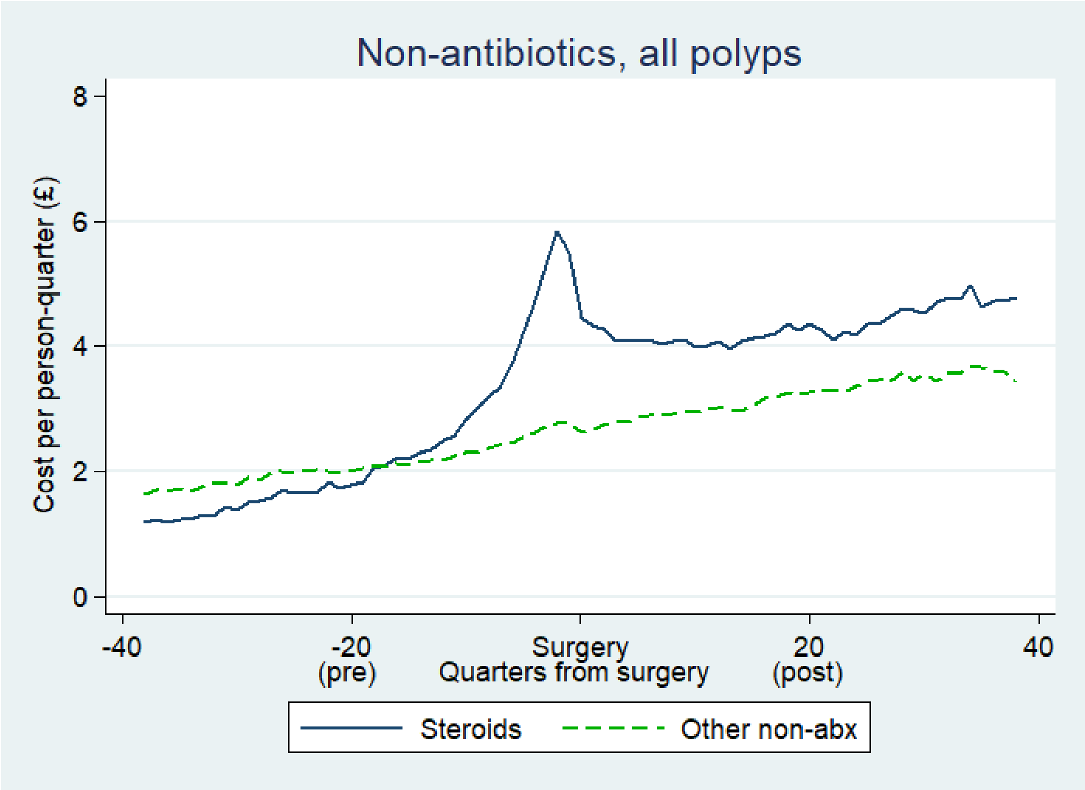

Clarke CS, et al. BMJ Open 2022; 12:e055603. doi: 10.1136/bmjopen-2021-055603
