# Supplementary material for: Observational retrospective study calculating health service costs of patients receiving surgery for chronic rhinosinusitis in England, using linked patient-level primary and secondary care electronic data
Source: BMJ Open. 2022 Feb 8;12(2):e055603. doi: 10.1136/bmjopen-2021-055603 (PMC8830221; doi:10.1136/bmjopen-2021-055603)
Supplement: Author's manuscript [file bmjopen-2021-055603.draft_revisions.pdf]

BMJ Open

BMJ Open is committed to open peer review. As part of this commitment we make the peer review history of every article we publish publicly available.

When an article is published we post the peer reviewers' comments and the authors' responses online. We also post the versions of the paper that were used during peer review. These are the versions that the peer review comments apply to.

The versions of the paper that follow are the versions that were submitted during the peer review process. They are not the versions of record or the final published versions. They should not be cited or distributed as the published version of this manuscript.

BMJ Open is an open access journal and the full, final, typeset and author-corrected version of record of the manuscript is available on our site with no access controls, subscription charges or pay-per-view fees (http://bmjopen.bmj.com).

If you have any questions on BMJ Open's open peer review process please email info.bmjopen@bmj.com

BMJ Open

Healthcare costs for patients receiving surgery for chronic rhinosinusitis in England, using linked primary and secondary care electronic patient-level data

Journal:	*BMJ Open*
Manuscript ID	bmjopen-2021-055603
Article Type:	Original research
Date Submitted by the Author:	26-Jul-2021
Complete List of Authors:	Clarke, Caroline; University College London, Research Department of Primary Care and Population Health<br>Williamson, Elizabeth; London School of Hygiene & Tropical Medicine, Department of Medical Statistics; Health Data Research UK<br>Denaxas, S; Health Data Research UK; University College London, Institute of Health Informatics<br>Carpenter, James; London School of Hygiene & Tropical Medicine, Department of Medical Statistics; University College London, MRC Clinical Trials Unit at UCL<br>Thomas, Mike; University of Southampton, Primary Care and Population Science<br>Blackshaw, Helen; ENT Clinical<br>Schilder, Anne; NIHR UCLH Biomedical Research Centre; University College London, Ear Institute<br>Philpott, Carl; University of East Anglia, Norwich Medical School; James Paget University Hospitals NHS Foundation Trust,<br>Hopkins, Claire; Guy's and St Thomas' NHS Foundation Trust, ENT<br>Morris, Stephen; University of Cambridge, Primary Care Unit, Department of Public Health and Primary Care
Keywords:	HEALTH ECONOMICS, OTOLARYNGOLOGY, Otolaryngology < SURGERY, Clinical trials < THERAPEUTICS

SCHOLARONE™ Manuscripts

I, the Submitting Author has the right to grant and does grant on behalf of all authors of the Work (as defined in the below author licence), an exclusive licence and/or a non-exclusive licence for contributions from authors who are: i) UK Crown employees; ii) where BMJ has agreed a CC-BY licence shall apply, and/or iii) in accordance with the terms applicable for US Federal Government officers or employees acting as part of their official duties; on a worldwide, perpetual, irrevocable, royalty-free basis to BMJ Publishing Group Ltd ("BMJ") its licensees and where the relevant Journal is co-owned by BMJ to the co-owners of the Journal, to publish the Work in this journal and any other BMJ products and to exploit all rights, as set out in our licence.

The Submitting Author accepts and understands that any supply made under these terms is made by BMJ to the Submitting Author unless you are acting as an employee on behalf of your employer or a postgraduate student of an affiliated institution which is paying any applicable article publishing charge ("APC") for Open Access articles. Where the Submitting Author wishes to make the Work available on an Open Access basis (and intends to pay the relevant APC), the terms of reuse of such Open Access shall be governed by a Creative Commons licence – details of these licences and which Creative Commons licence will apply to this Work are set out in our licence referred to above.

Other than as permitted in any relevant BMJ Author's Self Archiving Policies, I confirm this Work has not been accepted for publication elsewhere, is not being considered for publication elsewhere and does not duplicate material already published. I confirm all authors consent to publication of this Work and authorise the granting of this licence.

For peer review only

Title:

Healthcare costs for patients receiving surgery for chronic rhinosinusitis in England, using linked primary and secondary care electronic patient-level data

Running head: MACRO health care cost CRS surgery

Authors:

Caroline S Clarke[1]* (caroline.clarke@ucl.ac.uk), Elizabeth Williamson[2,3], Spiros Denaxas[3,4], James R Carpenter[2,3,5], Mike Thomas[6], Helen Blackshaw[7], Anne GM Schilder[8,9], Carl Philpott[10,11], Claire Hopkins[12] and Stephen Morris[13], on behalf of the MACRO programme team

Corresponding author: Caroline S Clarke (caroline.clarke@ucl.ac.uk)

Name	Affiliation
Caroline S Clarke[1]	1. Research Department of Primary Care and Population Health, University College London, London, UK.
Elizabeth Williamson[2,3],	2. Department of Medical Statistics, London School of Hygiene & Tropical Medicine, London, UK;<br>3. Health Data Research UK, London, UK
Spiros Denaxas[3,4],	3. Health Data Research UK, London, UK;<br>4. Institute of Health Informatics, University College London, London, UK
James R Carpenter[2,3,5],	2. Department of Medical Statistics, London School of Hygiene & Tropical Medicine, London, UK;<br>3. Health Data Research UK, London, UK;<br>5. MRC Clinical Trials Unit at UCL, London, UK
Mike Thomas[6],	6. Primary Care and Population Science, University of Southampton, UK
Helen Blackshaw[7]	7. ENT Clinical, Zeist, Netherlands
Anne GM Schilder[8,9]	8. NIHR University College London Hospitals Biomedical Research Centre,<br>9. Ear Institute, UCL, London, UK
Carl Philpott[10,11]	10. Norwich Medical School, University of East Anglia, Norwich, UK;<br>11. ENT Department, James Paget University Hospital NHS Foundation Trust, Great Yarmouth, UK
Claire Hopkins[12]	12. ENT Department, Guy's and St Thomas' NHS Foundation Trust, London, UK
Stephen Morris[13]	13. Primary Care Unit, Department of Public Health and Primary Care, University of Cambridge

For peer review only

For peer review only - http://bmjopen.bmj.com/site/about/guidelines.xhtml

Abstract:

Objectives

Chronic rhinosinusitis (CRS) symptoms are experienced by an estimated 11% of UK adults, and symptoms have major impacts on quality of life. Data from UK and elsewhere suggest high economic burden of CRS, but detailed cost information and economic analyses regarding surgical pathway are lacking. This paper estimates healthcare costs for patients receiving surgery for CRS in England.

Design

Observational retrospective study examining cost of healthcare of patients receiving CRS surgery.

Setting

Linked electronic health records from the Clinical Practice Research Datalink, Hospital Episode Statistics and Office for National Statistics databases in England.

Participants

A phenotyping algorithm utilising medical ontology terms identified "definite" CRS cases. Patients were registered with a general practice in England. Data covered the period 1997-2016. A cohort of 13,462 patients had received surgery for CRS, with 9,056 (67%) having confirmed nasal polyps.

Outcome measures

Resource use data were extracted on numbers and types of primary care prescriptions and consultations, and inpatient and outpatient hospital investigations and procedures. Resource use was costed using published sources.

Results

Total National Health Service costs in CRS surgery patients were £2,173 over one year including surgery. Total costs per person-quarter were £1,983 in the quarter containing surgery, mostly comprising inpatient care costs (£1,902), and around £60 per person-quarter in the 2 years before and after surgery, of which half were outpatient costs. Outpatient and primary care costs were low compared to the peak in inpatient costs at surgery. The highest outpatient expenditure was on computed tomography scans, peaking in the quarter preceding surgery.

Conclusions

We present the first English study of costs to the healthcare system in primary and secondary care for patients receiving surgery for CRS. The total aggregate costs provide a further impetus for trials to evaluate the relative benefit of surgical intervention.

For peer review only

For peer review only - http://bmjopen.bmj.com/site/about/guidelines.xhtml

Key words:

Cost of health care, chronic rhinosinusitis, observational data, surgery, electronic health records

HEALTH ECONOMICS, OTOLARYNGOLOGY, Otolaryngology < SURGERY, Clinical trials < THERAPEUTICS

Word count

3869/4000 not including tables, figures, legends, references.

Article summary

Strengths and Limitations

- Using linked patient-level primary and secondary care data covering 8% of the England population, we provide a comprehensive picture of the healthcare resources used for patients undergoing chronic rhinosinusitis surgery (CRS) as well as their costs
- Our work addresses a paucity of evidence regarding the direct costs of the surgical treatment pathway for CRS in England, and provides a valuable resource to aid commissioning decisions and future research for both surgery and alternative treatments for CRS in the UK
- The major limitation is the lack of a non-surgical comparison arm due to coding limitations within the dataset

For peer review only

For peer review only - http://bmjopen.bmj.com/site/about/guidelines.xhtml

1. Introduction

Chronic rhinosinusitis (CRS) represents a common source of ill health, affecting 5-12% of the general population [1]. In the UK, 11% of adults reported having CRS symptoms [2]. Symptoms, including nasal obstruction, nasal discharge, facial pain, anosmia and sleep disturbance, have major impacts on quality of life (QOL), which can be greater than the QOL impacts of chronic respiratory disease or angina [3]. In addition, expenditure on rhinosinusitis treatments has been estimated in the US as higher than for diseases such as ulcer disease, acute asthma and hay fever [4].

A worldwide study demonstrated that one-third of CRS patients in primary care have poorly controlled symptoms [5]; acute exacerbations, inadequate symptom control and respiratory disease exacerbation are common. The socio-economic cost of CRS is significant with 57% of patients reporting absenteeism in Sweden in 2008-09 [6], 28% experiencing associated anxiety and depression (UK, data collected 2007-2013) [7], and an estimated 19 missed work days per CRS patient per year (England, recruitment 2013-2015) [8]. In 2011, CRS cost the US healthcare system $8.6 billion with significant direct and indirect costs [9] [10]. Our recent systematic review of literature regarding the cost-effectiveness of surgical intervention confirms the lack of UK perspective economic evaluations, particularly relating to the UK healthcare system [11].

This study forms part of the MACRO Programme, "Defining best Management for Adults with Chronic RhinOsinusitis", and information from this cost analysis will supplement the analysis of the MACRO randomised controlled trial (RCT), which began recruitment in 2019 [12] [13]. The overarching aims of MACRO are to address major deficiencies in the evidence base for CRS management, establish best practice for management of adults with CRS, and design the ideal patient pathway across primary and secondary care. This observational cohort analysis of CRS surgery patients established the costs to the National Health Service (NHS) of treatments received by these patients from general practices/general practitioners (GPs) and in NHS hospitals in England as inpatients (including day cases) and outpatients, and estimated how much they cost, by polyp-defined subgroup as described below, using linked patient-level primary and secondary care electronic health record data and mortality data from the ONS. The total aggregated costs to the NHS provide a further impetus for trials to evaluate the benefit of surgical intervention.

2. Methods

2.1. Study design and population

Linked electronic health records (EHR) from the Clinical Practice Research Datalink (CPRD, primary care, covers ~8% of England population) [14], Hospital Episode Statistics (HES, covering inpatient and outpatient care provided in NHS hospitals in England) and Office for National Statistics (ONS, mortality data) databases were used. Scientific and ethical approval for the use of and data linkages within the CPRD primary care data was obtained following application to the Independent Scientific Advisory Committee (ISAC), a non-statutory expert advisory body (Protocol number 16_200). Data and phenotyping algorithms were accessed as part of the CALIBER resource [15] [16].

The population used in this analysis was a subset of the cohort used in previous work by this group that considered the risk of mortality and cardiovascular events following macrolide prescription in CRS patients [17]. An EHR phenotyping algorithm, comprising primary care and secondary care diagnoses and secondary care procedures deemed to indicate a 'definite' diagnosis of CRS, was developed in collaboration with

For peer review only - http://bmjopen.bmj.com/site/about/guidelines.xhtml

clinicians (see Supplementary Materials, Section A). Patients with one or more of these diagnoses or procedures recorded during follow-up were classified as 'definite' CRS cases, with the date of diagnosis taken to be the date of the first such specified diagnosis or procedure. A further list of 'definite' and 'very likely' surgery OPCS Classification of Interventions and Procedures version 4 (OPCS-4) codes was developed, and the cohort used in this cost analysis was the group of 'definite' CRS patients who had surgery defined as either 'definitely' or 'very likely' to have been for CRS (see Supplementary Materials, Section A).

Eligible patients entered the analysis cohort on the latest of: current general practice registration date of the patient, date on which research quality data began to be provided by the general practice (based on an internal CPRD algorithm [14]), their 16th birthday, or study start date (1 April 1997). Cases were required to have a minimum of one year's research-quality information prior to their CRS diagnosis, and a minimum of one day of research-quality data at an individual level following diagnosis. Patients left the cohort on the earliest of: transfer-out date from the general practice, last data collection from general practice, 80th birthday, death (recorded in either CPRD or ONS), or the study end date (29 February 2016).

A patient's follow-up period began on their CRS diagnosis date and ended when they left the cohort. The index date around which patients' treatment information was centred was the date on which the first CRS-specific surgery took place during the analysis period. Costs were calculated per patient-quarter, with the surgery date placed at the midpoint of quarter zero (Q0), so Q0 contained costs incurred during the 45.7 days before and after surgery as well as on the surgery date itself.

CRS has traditionally been divided into two main phenotypes, CRS with and without nasal polyps (CRSwNP and CRSsNP, respectively), with differences in underlying pathophysiology and association with other conditions such as asthma [18]. CRSwNP patients are more likely to have a higher disease burden and more likely to receive surgery [19]. Accordingly, participants were split into two sub-groups as in our previous work [17] [20], according to the patient's polyp status: positive polyp status, where polyps were specifically recorded or implied in the EHR at some point during the patient's follow-up (see Supplementary Materials, Section A); or unknown polyp status, meaning either that polyps were absent or that they were perhaps present but were not recorded.

2.2. Resource use and unit costs

Costs were calculated from an NHS perspective [21], and prices were in 2017-18 UK pounds sterling. Resource use data were extracted on numbers and types of consultations, investigations, procedures and prescriptions, and classified according to categories available in the relevant published unit costs.

Cost information was categorised for analysis according to the following five groups: (i) admitted patient care (APC) from HES APC events (costed as Day Case or Elective Inpatient); (ii) outpatient (OP) attendances from HES OP events; and (iii) primary care visits (GP contacts, practice nurse contacts, other primary care contacts), (iv) primary care antibiotics prescriptions, and (v) other relevant primary care prescriptions, with the latter three groups all from CPRD events data.

Inpatient and outpatient care codes were grouped into cost categories as detailed in Supplementary Materials, Table B1, and NHS Reference Costs [22] were applied. Inpatient care lasting less than 1 day according to the duration captured in CALIBER was costed as a Day Case, and stays longer than 1 day were costed as Elective Inpatient admissions. NHS Reference Costs from 2017-18 were used where available for that particular category, or earlier NHS Reference Costs were used where required, with uplift to 2017-18 prices using HCHS inflation indices [23]. This was required for outpatient complex sinus procedures (2016-17

For peer review only - http://bmjopen.bmj.com/site/about/guidelines.xhtml

prices were used and uplifted), and outpatient major sinus procedures (2015-16 prices were used and uplifted).

Unit costs and related information for primary care consultations were obtained from the Personal Social Services Research Unit (PSSRU) [23, 24] (see Supplementary Materials, Table B2). Longitudinal data from the CPRD which looked at GP contacts in England in 2010-2011 for respiratory tract infections suggested that 1% of adults received treatment for rhinosinusitis from their GP each year, with a median of 4 GP visits, and with 91% of these patients receiving an antibiotic prescription [25], so antibiotic prescriptions from primary care were analysed as a separate cost category. There were six commonly used antibiotics that were costed separately, and 38 less common antibiotics that were grouped together and a mean cost applied. The non-antibiotic medications comprised corticosteroids (including combinations with antihistamine) and all other drugs (i.e. painkillers, antihistamines, decongestants, and combinations thereof). Unit costs were obtained from the British National Formulary [26] (see Supplementary Materials, Table B3).

2.3. Statistical analysis

Poisson regression was used to calculate incidence rates per quarter (91.3 days), split by polyp status, and unit costs described above were applied to event rates to calculate costs.

Events were censored at 10 years before or after the surgery date for inpatient and primary care, and at 2 years before and after for outpatient care, as including events at dates further away led to small event numbers and therefore large uncertainties (see Supplementary Materials, Table C1, for the denominators at each timepoint, i.e. numbers of patients at risk of having a healthcare event at that moment according to their presence within the follow-up period). The total costs given here were therefore calculated in the period covering 2 years before and after surgery.

Discounting was not included as this analysis did not project future costs. Information from the electronic records was considered complete, so no imputation was performed. Stata v16 was used to run the analyses [27]. Mean per-person-quarter costs split according to the five categories listed above were calculated for the quarter containing the surgery date at its mid-point (Q0), and the mean per quarter for the 8 quarters before and 8 quarters after the surgery quarter, to provide estimates of costs for surgical patients both in the lead up to their surgery and in subsequent months, as well as around the surgery date itself. Total one-year surgery costs were also calculated per person by summing the 4 quarters from surgery, i.e. summing costs from Q0 (which contained surgery date at its mid-point), Q1, Q2, and Q3.

2.4. Patient and Public Involvement statement

Patient and public involvement collaborators are involved in the MACRO programme including its design, conduct, reporting and dissemination, but were not directly involved in the production of this cost analysis publication.

3. Results

3.1. Patient cohort and demographics

Of the 62,685 patients identified as definitely having CRS in 1997-2016 and registered in the GP practices covered by the CPRD in England, 13,462 received CRS-related surgery and were included in the cohort. Two-

For peer review only - http://bmjopen.bmj.com/site/about/guidelines.xhtml

thirds (9,056, 67%) were in the polyp-positive subgroup, with the rest (4,406, 33%) in the polyp-unknown subgroup. In the wider group including CRS-definite patients both with and without surgery (n=62,685), these proportions were reversed, namely one-third (23,036, 37%) were polyp-positive and two-thirds (39,649, 63%) were not. These proportions agree with other published work regarding the incidence of nasal polyps in CRS patients [19] [28] [29] [30]. Patient demographic information is in Table 1.

Table 1. Patient demographic information at surgery date.

	Unknown polyp status		Positive polyp status		All patients	
**Total patients, n**	4,406		9,056		13,462	
**Age in years, mean (SD)**	42.4 (14.6)		47.9 (14.7)		46.1 (14.9)	
	n	%	n	%	n	%
**Sex**						
Male	2,029	46.1	6,073	67.1	8,102	60.2
Female	2,377	53.9	2,983	32.9	5,360	39.8
**Ethnicity**						
White	4,038	91.6	8,264	91.3	12,302	91.4
India/South Asia	88	2.0	209	2.3	297	2.2
Black	45	1.0	68	0.8	113	0.8
China/East Asia	42	1.0	81	0.9	123	0.9
Mixed	51	1.2	120	1.3	171	1.3
Unknown	142	3.2	314	3.5	456	3.4
**Region of England**						
North East	51	1.2	179	2.0	230	1.7
North West	809	18.4	1,585	17.5	2,394	17.8
Yorkshire	208	4.7	444	4.9	652	4.8
East Midlands	126	2.9	287	3.2	413	3.1
West Midlands	399	9.1	1,044	11.5	1,443	10.7
East	516	11.7	1,109	12.2	1,625	12.1
South West	627	14.2	1,192	13.2	1,819	13.5
South Central	523	11.9	953	10.5	1,476	11.0
London	543	12.3	1,072	11.8	1,615	12.0
South East	604	13.7	1,191	13.2	1,795	13.3

3.2. Total costs

The total per-person costs to the NHS for one year in patients receiving surgery for CRS was £1,408 in those with unknown polyp status, £2,547 in those with known positive polyp status, and £2,173 overall for all patients. The majority of this expenditure took place in the quarter containing surgery (Table 2) and the highest single cost category was polypectomy in the polyps-positive group (Table 3). Table 2 shows the mean per-patient-quarter costs, total and by cost component, over the two-year period before the surgery date, during Q0 when surgery took place, and over the two-year period after surgery. Inpatient care costs peaked during Q0 and comprised the majority of Q0 costs. Outpatient costs during Q0 were approximately twice those in the before or after periods but small in comparison to Q0 inpatient costs. The cost of primary care consultations appeared to be lower during Q0 compared to the time preceding surgery and did not rebound in the following two years, and the two categories of primary care prescription costs were low at all times, with little apparent change around the surgery date. The standard errors for the mean per-patient-quarter costs in the 2 years before and after surgery are given in Table 4 but are omitted from Table 2 for readability purposes.

For peer review only - http://bmjopen.bmj.com/site/about/guidelines.xhtml

Table 2. Costs per patient-quarter, broken down by healthcare/prescription category, by time period, and by polyp status. Prices in 2017-2018 £; DC = Day Case; EL = Elective Inpatient; Abx = antibiotics.

	Inpatient care (DC and EL)	Outpatient	Primary care consultations	Primary care Abx	Primary care Non-abx	TOTAL
**Mean per-patient-quarter costs over 2 years before surgery (-Q1 to -Q8)**						
Unknown polyps (£)	3.35	40.83	16.08	1.70	5.87	**67.82**
Positive polyps (£)	1.53	29.69	16.68	1.15	7.64	**56.69**
All patients (£)	2.13	33.49	16.49	1.33	7.06	**60.50**
**Per person-quarter (in Q0, containing index surgery)**						
Unknown polyps (£)	1117.37	75.68	7.04	1.27	5.54	**1206.90**
Positive polyps (£)	2284.63	62.41	5.59	0.99	7.79	**2361.42**
All patients (£)	1902.00	66.75	6.06	1.08	7.06	**1982.95**
**Mean per-patient per-quarter costs over 2 years after surgery (Q1 to Q8)**						
Unknown polyps (£)	8.64	37.71	6.43	1.26	5.50	**59.54**
Positive polyps (£)	20.70	25.60	4.73	0.95	7.63	**59.62**
All patients (£)	16.87	29.46	5.27	1.05	6.96	**59.61**

3.3. *Admitted patient care – Day Case (<1day) and Elective Inpatient (>1 day)*

The cost of hospital admissions was £2.13 (SE £1.18) per patient-quarter in the 8 quarters leading up to the surgery quarter (£1.53 (SE £0.93) in polyps-positive patients and £3.35 (SE £2.11) in polyps-unknown patients) (see Table 4). The majority of hospital admission costs were accrued around surgery during Q0 (£1,902 overall; £1,117 in polyps-unknown patients and £2,285 in polyps-positive patients), and costs per patient-quarter were lower than this peak in the subsequent 8 quarters, at around £17 (SE £3) per patient-quarter (see Table 4).

Table 3 shows the cost breakdown during Q0. The highest expenditure in polyp-positive patients was on Polypectomy (E081), covering around a third of all events in this group, and a further 40% corresponded to one of functional endoscopic sinus surgery (FESS), intranasal antrostomy, or intranasal ethmoidectomy, which together formed the major part of the Intermediate/Major/Complex sinus procedures group. In polyp-unknown patients, the highest expenditure was on FESS, intranasal antrostomy, or intranasal ethmoidectomy, which again formed the major part of the Intermediate/Major/Complex sinus procedures group. Types of procedures were grouped together as seen in Table 3 as some codes had small event numbers, thus regressions did not converge unless some groupings were made beyond the categories listed in Supplementary Materials Table B1. Groupings were made based on consecutive unit costs in Elective Inpatient data and the same groupings were used in Day Case data for consistency of reporting. Tables showing costs split by category and polyp subgroup are given in the Supplementary Materials, Section D.

For peer review only - http://bmjopen.bmj.com/site/about/guidelines.xhtml

Table 3. Mean inpatient costs per patient-quarter in Q0 by procedure category, split by polyp status. CT = computed tomography; DC = Day Case; EL = Elective Inpatient; Q0 = quarter containing surgery date at centre. Prices in 2017-2018 £.

Q0	CT/other imaging, DC	Minor nose incl. biopsy, DC	Int nose and Minor sinus, DC	Int/Major/ Complex sinus, DC	CT/other imaging, EL	Minor nose incl. biopsy, EL	Int nose and Minor sinus, EL	Int/Major/ Complex sinus, EL	Polyp-ectomy	TOTAL (Q0)
**Unknown polyps**	0.16	8.62	43.86	243.32	0.86	29.10	110.97	680.49	0.00	**1,117.37**
**Positive polyps**	0.02	7.85	40.09	152.60	0.14	28.44	166.57	474.22	1,414.69	**2,284.63**
**All patients**	0.07	8.10	41.32	181.87	0.37	28.66	148.42	540.56	952.62	**1,902.00**

Table 4. Costs during the surgery quarter (Q0) and 2 years before and after. Prices in 2017-2018 £. SE = standard error, DC = Day Case, EL= Elective Inpatient.

	Per-patient costs over 2 years preceding surgery	Mean (SE) per person-quarter over 2 years preceding surgery	Per-patient costs in the quarter containing surgery (Q0)	Mean (SE) per person-quarter over 2 years following surgery	Per-patient costs over 2 years following surgery
**Inpatient costs (DC and EL)**					
Unknown polyps	26.81	3.35 (2.11)	**1,117.37**	8.64 (2.97)	69.15
Positive polyps	12.26	1.53 (0.93)	**2,284.63**	20.70 (4.56)	165.61
All patients	17.02	2.13 (1.18)	**1,902.00**	16.87 (2.97)	134.96
**Outpatient costs**					
Unknown polyps	326.61	40.83 (12.22)	**75.68**	37.71 (8.40)	301.69
Positive polyps	237.49	29.69 (11.41)	**62.41**	25.60 (4.64)	204.77
All patients	267.93	33.49 (11.57)	**66.75**	29.46 (5.78)	235.67
**Primary care consultations**					
Unknown polyps	128.64	16.08 (5.09)	**7.04**	6.43 (0.62)	51.47
Positive polyps	133.48	16.68 (7.02)	**5.59**	4.73 (0.16)	37.87
All patients	131.91	16.49 (6.28)	**6.06**	5.27 (0.28)	42.18
**Primary Care Antibiotics Prescriptions**					
Unknown polyps	13.57	1.70 (0.35)	**1.27**	1.26 (0.04)	10.05
Positive polyps	9.20	1.15 (0.20)	**0.99**	0.95 (0.03)	7.60
All patients	10.63	1.33 (0.24)	**1.08**	1.05 (0.02)	8.38
**Primary Care Non-Antibiotics Prescriptions**					
Unknown polyps	46.93	5.87 (0.80)	**5.54**	5.50 (0.09)	43.96
Positive polyps	61.12	7.64 (1.25)	**7.79**	7.63 (0.07)	61.08
All patients	56.48	7.06 (1.10)	**7.06**	6.96 (0.05)	55.65

For peer review only - http://bmjopen.bmj.com/site/about/guidelines.xhtml

3.4. *Outpatient attendances*

The cost of outpatient care was £33.49 (SE £11.57) per patient-quarter in the 8 quarters preceding surgery; (£29.69 (SE £11.41) in polyps-positive patients and £40.83 (SE £12.22) in polyps-unknown patients) (see Table 4), then £66.75 during the surgery quarter (£62.41 in polyps-positive patients and £75.68 in polyps-unknown patients). Costs per patient-quarter were reduced from this peak in the subsequent 8 quarters, at around £30 per patient-quarter (see Table 4).

Table 5 shows the breakdown of costs during Q0 and the quarters immediately preceding and succeeding Q0. The highest expenditure in both subgroups was on CT (computed tomography)/other scans, which comprised around two-thirds CT scans and one-third X-rays. All categories showed a peak in costs in Q0 except for CT/other scans, which instead had a slightly higher peak in the quarter immediately preceding surgery (see Table 5). This tallies with the advice in EPOS 2020 stating that CT scans should always be given before surgery [1]. Tables showing the values split by category and by polyp subgroup, and graphs illustrating this information (i.e. expanding on the information presented in Table 5), are given in Supplementary Materials, Section E.

Table 5. Mean outpatient costs per person-quarter in Q0 and the immediately preceding and succeeding quarters, by procedure category, split by polyp status. CT = computed tomography; Q0 = quarter containing surgery date at centre. Prices in 2017-2018 £.

	CT/other imaging	Minor nose incl. biopsy	Int nose and Minor sinus	Int sinus	Major/ complex sinus	Polyp-ectomy	TOTAL (by person-quarter)
**Polyps unknown**							
**-Q1**	32.30	4.55	11.26	14.70	5.18	-	**67.99**
**Q0**	**29.11**	**4.37**	**13.95**	**17.65**	**10.59**	**-**	**75.68**
**Q1**	25.51	3.16	12.17	12.35	5.51	-	**58.70**
**Polyps positive**							
**-Q1**	25.04	1.84	11.36	12.02	6.17	0.44	**56.87**
**Q0**	**23.03**	**2.93**	**12.47**	**14.46**	**8.66**	**0.85**	**62.41**
**Q1**	14.88	1.63	7.57	8.64	2.97	0.32	**36.00**
**All patients**							
**-Q1**	27.49	2.76	11.33	12.93	5.83	0.29	**60.62**
**Q0**	**25.01**	**3.40**	**12.96**	**15.51**	**9.29**	**0.57**	**66.75**
**Q1**	18.32	2.13	9.06	9.84	3.79	0.21	**43.35**

3.5. *Primary care consultations*

The cost of primary care consultations was £16.49 (SE £6.28) per patient-quarter in the 8 quarters preceding surgery (£16.68 (SE £7.02) in polyps-positive patients and £16.08 (SE £5.09) in polyps-unknown patients) (see Table 4), then £6.06 during the surgery quarter (£7.04 in polyps-unknown patients, £5.59 in polyps-positive patients), and costs per patient-quarter were similarly reduced in the subsequent 8 quarters, at around £5-6 per patient-quarter (see Table 4). The highest expenditure in both subgroups was GP face-to-face consultations at the GP practice. Tables showing the values split by category and by polyp subgroup, and graphs illustrating this information, are given in Supplementary Materials, Section F.

3.6. *Primary care prescriptions – antibiotics*

The cost of primary care antibiotics prescriptions was £1.33 (SE £0.24) per patient-quarter in the 8 quarters before surgery (£1.15 (SE £0.20) in polyps-positive patients and £1.70 (SE £0.35) in polyps-unknown patients), then £1.08 during Q0 (£1.27 in polyps-unknown patients, £0.99 in polyps-positive patients), and similar in

For peer review only - http://bmjopen.bmj.com/site/about/guidelines.xhtml

the subsequent 8 quarters, at around £1 per patient-quarter (see Table 4). The highest expenditure was on tetracyclines, followed by macrolides, and tables showing the values split by category and by polyp subgroup, and graphs illustrating this information, are shown in the Supplementary Materials, Section G.

3.7. Primary care prescriptions – steroids and other non-antibiotics

The cost of primary care non-antibiotics prescriptions was based primarily on corticosteroids, plus general sinusitis drugs like painkillers and decongestants, and was £7.06 per patient-quarter in the 8 quarters before surgery (£7.64 in polyps-positive patients and £5.87 in polyps-unknown patients), then £7.06 during the surgery quarter (£7.79 in polyps-unknown patients, £5.54 in polyps-positive patients), and similar in the subsequent 8 quarters, at around £7 per patient-quarter (see Table 4). Tables showing the values split by category and by polyp subgroup, and graphs illustrating this information, are given in Supplementary Materials, Section H. This information includes only prescriptions made by the GP, and does not include other medications bought over the counter by the patient.

For peer review only

4. Discussion

In this paper, we have shown that inpatient surgical sinus procedures and nasal polypectomies are the largest healthcare cost in patients receiving surgery for CRS when considering the costs of primary and secondary care to the NHS, at around £1000-2000 per person-quarter in the quarter containing the surgery date (Q0). Other secondary and primary healthcare costs in the 8 quarters before and after Q0 are considerably smaller, at around £60 per person-quarter across polyp subgroups.

Average total costs across secondary and primary care settings were £1,983 per patient overall during Q0, or £2,361 per polyp-positive patient and £1,207 per polyp-unknown patient, in 2017-2018 prices. Hospital overnight admission and day case inpatient costs incurred during the surgery quarter were the costliest category across the 4.25-year analysis period, dwarfing other cost components. Primary care prescription costs were low across both groups, with antibiotics costing around £1 per person-quarter and non-antibiotics around £7 per person-quarter. Outpatient care costs appeared higher than primary care costs at around £30 per person-quarter before and after surgery, and around £67 per person-quarter during Q0. Primary care consultation costs appeared higher before surgery than after (£16 vs. £6 per person-quarter), and inpatient care costs appeared higher after surgery than before (£17 vs. £2 per person-quarter). These findings suggest that the costs to the NHS associated with CRS, especially the non-surgical costs, are currently low. They also suggest that CRS surgery does not appreciably impact overall management costs, either upwards or downwards, although these costs are low so it would be difficult to see a meaningful change. These values are presented as descriptive statistics and formal significance testing among the various categories and timepoints described above has not been performed.

There were certain limitations in this analysis. Only costs for those patients for whom CRS surgery codes were recorded during the time period were included, and the analysis was based around the date of their first CRS surgery as captured during the analysis time period. Regarding second surgeries, 0.4% of patients in this analysis had a second surgery during the second half of Q0 after their index surgery, and 4.9% of patients received a second surgery during the 8 quarters following Q0.

For peer review only - http://bmjopen.bmj.com/site/about/guidelines.xhtml

This analysis used CPRD for the information on primary care, thus restricting the analysis to a dataset covering around 8% of the population of England, which is considered to be broadly representative although with acknowledged gaps including people who are universally underrepresented in UK healthcare systems, for example homeless people and those with non-standard residency or migration status [31]. Other limitations relate to other aspects of coding and identification of patients, as the information in the dataset used was collected by hospitals and GP practices for reimbursement and clinical management purposes, and not specifically for research purposes, and patients were not prospectively recruited into the dataset so there was no prospectively defined baseline. For example, identification of CRS patients and their diagnosis dates was performed using phenotyping codelists of treatments and diagnostic markers, using methodology common to observational analyses using routine data and expert clinical opinion to determine the codelists. Thus the identification of patients was reliant on patients' practitioners or coding staff having entered certain codes or combinations of codes. Furthermore, the coding regarding polyp status is limited, as there is no code to confirm that a patient does not have polyps, there is only the absence of a positive report of polyps being observed. This is based on treatments recorded, including the reporting of a polypectomy, leading to a certain circularity when reporting the treatments received by subgroup.

We used the standard English NHS cost perspective regarding treatment in primary and secondary care, although we did not have information on Personal Social Services, the costs of which would normally be included in analyses for the National Institute for Health and Care Excellence (NICE) [21], or on other community-based health care such as Improving Access to Psychological Therapies (IAPT), which might be relevant to this population. We had no information on wider societal costs, for example relating to productivity (time off work) or any out-of-pocket costs for patients. It is possible therefore that information regarding factors that are important to patients and their families was not captured in this analysis. Other work published in this area has focused mostly on US costs and used different unit costs and included different cost categories. Bhattacharyya et al. [32] investigated costs of CRSwNP patients in a US claims database using information gathered in 2013-2014, beginning at diagnosis of CRS. When the subgroup of CRSwNP undergoing FESS was compared to the subgroup of CRSwNP patients not undergoing surgery, they found that the extra cost of surgery during that first year was $13,532. This was an observational, retrospective case-control study, meaning that treatment decisions were not randomly assigned within the CRSwNP group, and therefore any differences in costs according to treatment decisions were susceptible to selection bias. Studies have also been published that examine cost breakdowns of CRS patients in the US regarding the distribution of expenditure across different categories of care. For example, Caulley et al. [33] considered all CRS patients in the US Medical Expenditure Panel Survey, taking a cross section in 2011, and found that ambulatory office-based consultations and prescriptions each accounted for a greater proportion of expenditure than inpatient hospital visits, although this was for all CRS patients, not just those receiving surgery, and the US system is both structured and financed quite differently from the UK system. Bhattacharyya et al. [32] however reported that prescription costs were not a major part of CRS costs for CRS patients undergoing surgery or not undergoing surgery in their observational study using the Truven Health MarketScan US claims database.

We have not attempted to compare treatments received by surgical and non-surgical patients, as this is difficult to do in observational datasets and can lead to misleading results. The limitation is that as patients are not randomly allocated to receive their treatment, there are unobserved and unmeasured confounders that instead can govern what treatment people have received. RCTs aim to identify and capture these confounders, using a large enough sample size that they are balanced between the arms, and control for them in the analysis. There are methods such as instrumental variable analysis that attempt to mimic randomisation using statistical methods, but it is typically hard to find a suitable instrument [34] [35]. Using

For peer review only - http://bmjopen.bmj.com/site/about/guidelines.xhtml

random allocation to assign treatments is therefore a powerful tool in eliminating selection bias, and is not directly available in analysis using routine observational data, hence the importance of the MACRO RCT [12], which began recruiting patients in 2019. MACRO is randomising patients 1:1:1 to receive appropriate medical therapy (AMT), surgery plus AMT, or long-term low-dose macrolides plus AMT, and collecting all relevant information required to make a randomised comparison between surgery and non-surgical treatments in a full cost-utility analysis [36] [37] [38]. The MACRO RCT will provide key information regarding changes in quality of life on receiving surgery for CRS and allow us to provide information regarding the relative cost-effectiveness of surgery and other treatments in the UK context.

5. Conclusion

This is the first study that we are aware of that analysed the costs of primary and secondary healthcare received by patients undergoing surgery for chronic rhinosinusitis using English NHS costs. It included a large sample size that was representative of care given in England and showed that the inpatient costs including CRS surgery itself were around £2000 during the quarter containing surgery, and that the cost of management before and after surgery in primary and secondary care settings was low in comparison at around £60 per person-quarter in the two preceding and subsequent years.

This study reports important new evidence regarding the cost of English NHS healthcare costs for patients receiving CRS surgery, and provides further justification for the use of randomised clinical trials to investigate the relative cost-effectiveness of surgical treatments for CRS, as well as providing useful information that can be applied in future work in the UK and similar contexts, including our own future analysis of the MACRO trial data.

Acknowledgements

The authors wish to acknowledge the valuable input from the MACRO WS1 study team and the wider MACRO programme team, including our Patient and Public Involvement Collaborators.

Contributorship statement

CSC was lead author, and wrote the first draft of the article. CSC and EW cleaned and analysed the data regarding rates of events. CSC and SM planned and conducted the cost analyses. EW, CSC, SM, MT, CH and CP formulated the phenotyping codelists for identifying the patient cohort and relevant treatments and diagnoses. All authors were involved in formulating the overall research question, and designing and conducting the study. All authors contributed to and approved the final manuscript.

Competing interests:

In addition to the NIHR funding for this work acknowledged above, the following interests outside the submitted work have been declared by the authors. EW: personal fees from AstraZeneca for provision of training on propensity score methodology unrelated to the current manuscript; JC: grants from UK Medical Research Council (grant numbers MC_UU_12023/21 and MC_UU_12023/29), consultancy from AstraZeneca and Novartis on statistical methodology for the analysis of partially observed data, and book royalties from “Multiple Imputation and its Application” (Wiley) and “Meta-analysis using R” (Springer); CP: personal fees for advisory work from GSK and Sanofi, and work as a Trustee of Fifth Sense; CH: Advisory

For peer review only - http://bmjopen.bmj.com/site/about/guidelines.xhtml

board for GSK, Sanofi and AstraZeneca and speakers fees for Mylan and Intersect. The remaining authors have nothing to declare.

Funding

This work was supported by the National Institute for Health Research (NIHR) under its Programme Grants for Applied Research (PGfAR) Programme (Grant Reference Number RP-PG-0614-20011). The views expressed are those of the author(s) and not necessarily those of the National Health Service, the National Institute for Health Research, or the Department of Health and Social Care.

Data sharing statement

The data were pseudonymised patient-level data from the CALIBER resource and are not publicly available. Analysis code can be made available on reasonable request and in accordance with relevant guidance.

Ethics statement

Not applicable as human participants were not directly included in this work. This study used routinely collected data. Scientific and ethical approval for the use of and data linkages within the Clinical Practice Research Datalink (CPRD) primary care data was obtained following application to the Independent Scientific Advisory Committee (ISAC), a non-statutory expert advisory body (Protocol number 16_200).

6. References

[1] W. J. Fokkens, V. J. Lund, C. Hopkins, P. W. Hellings, R. Kern, S. Reitsma, M. Bernal-Sprekelsen and J. Mullol, "European Position Paper on Rhinosinusitis and Nasal Polyps 2020," *Rhinology,* vol. 58, no. Suppl. 29, pp. 1-464, 2020.

[2] D. Hastan, W. J. Fokkens, C. Bachert, R. B. Newson, J. Bislimovska, A. Bockelbrink, P. J. Bousquet, G. Brozek, A. Bruno, S. E. Dahlen, B. Forsberg, M. Gunnbjörnsdóttir, L. Kasper, U. Krämer, M. L. Kowalski, B. Lange, B. Lundbäck, E. Salagean, A. Todo-Bom, P. Tomassen, E. Toskala, C. M. van Drunen, J. Bousquet, T. Zuberbier, D. Jarvis and P. Burney, "Chronic rhinosinusitis in Europe - an underestimated disease. A GA²LEN study.," *Allergy,* vol. 66, no. 9, pp. 1216-1223, 2011.

[3] R. E. Gliklich and R. Metson, "The health impact of chronic sinusitis in patients seeking otolaryngologic care," *Otolaryngology - Head and Neck Surgery,* vol. 113, no. 1, pp. 104-109, 1995.

[4] N. Bhattacharyya, "Contemporary Assessment of the Disease Burden of Sinusitis," *Allergy & Rhinology,* vol. 1, no. 1, 2010.

[5] B. Lange, R. Holst, T. Thilsing, J. Baelum and A. Kjeldsen, "Quality of life and associated factors in persons with chronic rhinosinusitis in the general population: A prospective questionnaire and clinical cross-sectional study," *Clinical Otolaryngology,* vol. 38, no. 6, pp. 474-480, 2013.

[6] P. Sahlstrand-Johnson, B. Ohlsson, C. von Buchwald, M. Jannert and M. Ahlner-Elmqvist, "A multi-centre study on quality of life and absenteeism in patients with CRS referred for endoscopic surgery," *Rhinology ,* vol. 49, no. 4, pp. 420-428, 2011.

For peer review only - http://bmjopen.bmj.com/site/about/guidelines.xhtml

For peer review only

[7] S. E. Erskine, C. Hopkins, A. Clark, S. Anari, A. Robertson, S. Sunkaraneni, J. A. Wilson, J. Beezhold and C. M. Philpott, "Chronic rhinosinusitis and mood disturbance," *Rhinology,* vol. 55, no. 2, pp. 113-119, 2017.

[8] N. W. Wahid, R. Smith, A. Clark, M. Salam and C. M. Philpott, "The socioeconomic cost of chronic rhinosinusitis study," *Rhinology,* vol. 58, no. 2, pp. 112-125, 2020.

[9] V. K. Anand, "Epidemiology and economic impact of rhinosinusitis," *Annals of otology, rhinology & laryngology, Suppl.,* vol. 193, pp. 3-5, 2004.

[10] N. Bhattacharyya , "The economic burden and symptom manifestations of chronic rhinosinusitis," *American Journal of Rhinology & Allergy,* vol. 17, no. 1, pp. 27-32, 2003.

[11] A. Bernic, O. Dessouky, C. Philpott, S. Morris and C. Hopkins, "Cost-effective surgical intervention in chronic rhinosinusitis," *Current Otorhinolaryngology Reports,* vol. 3, pp. 117-123, 2015.

[12] C. Philpott, S. le Conte, D. Beard, J. Cook, W. Sones, S. Morris, C. S. Clarke, M. Thomas, P. Little, J. Vennik, V. Lund, H. Blackshaw, A. Schilder, S. Durham, S. Denaxas, J. Carpenter, J. Boardman and C. Hopkins, "Clarithromycin and endoscopic sinus surgery for adults with chronic rhinosinusitis with and without nasal polyps: study protocol for the MACRO randomised controlled trial," *Trials ,* vol. 20, p. 246, 2019.

[13] H. Blackshaw, J. Vennik, C. Philpott, M. Thomas, C. Eyles, J. Carpenter, C. S. Clarke, S. Morris, A. Schilder, V. Lund, P. Little, S. Durham, S. Denaxas, E. Williamson, D. Beard, J. Cook, S. le Conte, K. Airey, J. Boardman and C. Hopkins, "Expert panel process to optimise the design of a randomised controlled trial in chronic rhinosinusitis (the MACRO programme)," *Trials,* vol. 20, p. 230, 2019.

[14] E. Herrett, A. M. Gallagher, K. Bhaskaran, H. Forbes, R. Mathur, T. van Staa and L. Smeeth, "Data Resource Profile: Clinical Practice Research Datalink (CPRD)," *International Journal of Epidemiology,* vol. 44, no. 3, pp. 827-836, 2015.

[15] S. Denaxas, A. Gonzalez-Izquierdo, K. Direk, N. K. Fitzpatrick, G. Fatemifar, A. Banerjee, R. J. Dobson, L. J. Howe, V. Kuan, R. T. Lumbers, L. Pasea, R. S. Patel, A. D. Shah, A. D. Hingorani, C. Sudlow and H. Hemingway, "UK phenomics platform for developing and validating electronic health record phenotypes: CALIBER," *Journal of the American Medical Informatics Association,* vol. 26, no. 12, p. 1545–1559, 2019.

[16] V. Kuan, S. Denaxas, A. Gonzalez-Izquierdo, K. Direk, O. Bhatti, S. Husain, S. Sutaria, M. Hingorani, D. Nitsch, C. A. Parisinos, R. T. Lumbers, R. Mathur, R. Sofat, J. P. Casas, I. C. Wong, H. Hemingway and A. D. Hingorani, "A chronological map of 308 physical and mental health conditions from 4 million individuals in the English National Health Service," *Lancet Digital Health,* vol. 1, pp. e63-77, 2019.

[17] E. Williamson, S. Denaxas, S. Morris, C. S. Clarke, M. D. Thomas, H. Evans, K. Direk, A. Gonzalez-Izquierdo, P. Little, V. Lund, H. Blackshaw, A. Schilder, C. Philpott, C. Hopkins and J. Carpenter, "Risk of mortality and cardiovascular events following macrolide prescription in chronic rhinosinusitis patients: a cohort study using linked primary care electronic health records," *Rhinology,* vol. 57, no. 4, pp. 252-260, 2019.

[18] C. Hopkins, C. Philpott, S. Carrie, J. Blythe, M. Thomas, P. Little, G. Scadding, V. J. Lund, S. Wilkes, A. Swift and H. Saleh, "Commissioning guide: Chronic Rhinosinusitis," ENT UK, 2016.

For peer review only - http://bmjopen.bmj.com/site/about/guidelines.xhtml

[19] A. Banerji, J. F. Piccirillo, S. E. Thawley, R. G. Levitt, K. B. Schechtman, M. A. Kramper and D. L. Hamilos, "Chronic rhinosinusitis patients with polyps or polypoid mucosa have a greater burden of illness," *American Journal of Rhinology,* vol. 21, pp. 19-26, 2007.

[20] C. Hopkins, E. Williamson, S. Morris, C. S. Clarke, M. Thomas, H. Evans, P. Little, V. J. Lund, H. Blackshaw, A. Schilder, C. Philpott, J. Carpenter and S. Denaxas, "Antibiotic usage in chronic rhinosinusitis: analysis of national primary care electronic health records," *Rhinology,* vol. 57, no. 6, pp. 420-442, 2019.

[21] NICE, "Guide to the methods of technology appraisal," 2013. [Online]. Available: https://www.nice.org.uk/process/pmg9.

[22] NHS Improvement, "NHS Reference Costs," [Online]. Available: https://improvement.nhs.uk/resources/national-cost-collection/.

[23] L. Curtis and A. Burns, "Unit Costs of Health and Social Care 2018," Personal Social Services Research Unit (PSSRU), University of Kent, Canterbury, 2018.

[24] L. Curtis and A. Burns, "Unit Costs of Health and Social Care 2015," Personal Social Services Research Unit (PSSRU), University of Kent, Canterbury, 2015.

[25] M. C. Gulliford, A. Dregan, M. V. Moore, M. Ashworth, T. van Staa, G. McCann, J. Charlton, L. Yardley, P. Little and L. McDermott, "Continued high rates of antibiotic prescribing to adults with respiratory tract infection: survey of 568 UK general practices," *BMJ Open,* vol. 4, no. 10, p. e006245, 2014.

[26] Joint Formulary Committee, "British National Formulary (online)," London.

[27] StataCorp, *Stata Statistical Software: Release 16,* College Station, TX, USA: StataCorp LP, 2019.

[28] C. Hopkins, J. P. Browne, R. Slack, V. Lund, J. Topham, B. Reeves, L. Copley, P. Brown and J. van der Meulen, "The national comparative audit of surgery for nasal polyposis and chronic rhinosinusitis," *Clinical Otolaryngology,* vol. 31, no. 5, pp. 390-398, 2006.

[29] C. Hopkins, P. Andrews and C. E. Holy, "Does time to endoscopic sinus surgery impact outcomes in chronic rhinosinusitis? Retrospective analysis using the UK clinical practice research data," *Rhinology,* vol. 53, pp. 18-24, 2015.

[30] E. S. Lourijsen, C. A. de Borgie, M. Vleming and W. J. Fokkens, "Endoscopic sinus surgery in adult patients with chronic rhinosinusitis with nasal polyps (PolypESS): study protocol for a randomised controlled trial," *Trials,* vol. 18, p. 39, 2017.

[31] T. Elwell-Sutton, J. Fok, F. Albanese, H. Mathie and R. Holland, "Factors associated with access to care and healthcare utilization in the homeless population of England," *Journal of Public Health,* vol. 39, no. 1, pp. 26-33, 2017.

[32] N. Bhattacharyya, S. Villeneuve, V. N. Joish, C. Amand, L. Mannent, N. Amin, P. Rowe, J. Maroni, L. Eckert, T. Yang and A. Khan, "Cost Burden and Resource Utilization in Patients With Chronic Rhinosinusitis and Nasal Polyps," *Laryngoscope,* vol. 129, pp. 1969-1975, 2019.

[33] L. Caulley, K. Thavorn, L. Rudmik, C. Cameron and S. J. Kilty, "Direct costs of adult chronic rhinosinusitis by using," *Journal of Allergy and Clinical Immunology,* vol. 136, no. 6, pp. 1517-1522, 2015.

For peer review only

For peer review only - http://bmjopen.bmj.com/site/about/guidelines.xhtml

[34] A. Basu, J. J. Heckman, S. Navarro-Lozano and S. Urzua, “Use of instrumental variables in the presence of heterogeneity and self-selection: an application to treatments of breast cancer patients,” *Health Economics,* vol. 16, pp. 1133-1157, 2007.

[35] A. Basu, “Estimating person-centered treatment (PeT) effects using instrumental variables: an application to evaluating prostate cancer treatments,” *Journal of Applied Econometrics,* vol. 29, no. 4, pp. 671-691, 2014.

[36] G. A. Scangas, A. K. Remenschneider, B. M. Su, M. G. Shrime and R. Metson, “Cost Utility Analysis of Endoscopic Sinus Surgery for Chronic Rhinosinusitis With and Without Nasal Polyposis,” *The Laryngoscope,* vol. 127, p. 29–37, 2017.

[37] L. Rudmik, Z. M. Soler, J. C. Mace, R. J. Schlosser and T. L. Smith, “Economic Evaluation of Endoscopic Sinus Surgery versus Continued Medical Therapy for Refractory Chronic Rhinosinusitis,” *Laryngoscope,* vol. 125, no. 1, pp. 25-32, 2015.

[38] G. A. Scangas, B. M. Su, A. K. Remenschneider, M. G. Shrime and R. Metson, “Cost utility analysis of endoscopic sinus surgery for chronic rhinosinusitis,” *International Forum of Allergy & Rhinology,* vol. 6, no. 6, pp. 582-589, 2016.

For peer review only

For peer review only - http://bmjopen.bmj.com/site/about/guidelines.xhtml

Title:

Healthcare costs for patients receiving surgery for chronic rhinosinusitis in England, using linked primary and secondary care electronic patient-level data

Authors:

Caroline S Clarke* (caroline.clarke@ucl.ac.uk), Elizabeth Williamson, Spiros Denaxas, James R Carpenter, Mike Thomas, Helen Blackshaw, Anne GM Schilder, Carl Philpott, Claire Hopkins and Stephen Morris, on behalf of the MACRO programme team

Supplementary Materials

Section A – codelists

CRS diagnostic terms – definite CRS

The following codelist was developed to identify patients with chronic rhinosinusitis, according to [illegible] diagnostic events. Patients with one or more of these diagnoses or procedures recorded during follow-up were considered to be definite CRS cases, with the date of diagnosis taken to be the date of the first such diagnosis or procedure.

Code type	ICD-10 Code		Description ("definite" CRS diagnosis)
ICD-10	J32		Chronic sinusitis
	J320		Chronic maxillary sinusitis
	J321		Chronic frontal sinusitis
	J322		Chronic ethmoidal sinusitis
	J323		Chronic sphenoidal sinusitis
	J324		Chronic pansinusitis
	J328		Other chronic sinusitis
	J329		Chronic sinusitis, unspecified
	J33		Nasal polyp
	J330		Polyp of nasal cavity
	**MEDCODE**	**Read code**	
MEDCODE with corresponding Read Code	811	7406000	Nasal polypectomy
	848	7411200	Intranasal antrostomy
	977	H110.00	Polyp of nasal cavity
	1673	7412100	Intranasal ethmoidectomy
	1674	H132.00	Chronic ethmoidal sinusitis
	2257	H13..00	Chronic sinusitis
	4433	H130.00	Chronic maxillary sinusitis
	4686	H11..00	Nasal polyps
	5437	H13z.00	Chronic sinusitis NOS
	6411	2D33.00	O/E - nasal polyp present

For peer review only - http://bmjopen.bmj.com/site/about/guidelines.xhtml

For peer review only

	6491	7416	FESS/Therapeutic endoscopy of nose and sinus
	7243	7.42E+03	FESS/Post operative division of adhesions
	8220	7416300	FESS/Uncinectomy
	10546	H13..11	Chronic rhinosinusitis
	11744	H11y100	Polyp of ethmoidal sinus
	14749	H110z00	Polyp of nasal cavity NOS
	14888	H11z.00	Nasal polyp NOS
	15163	H131.00	Chronic frontal sinusitis
	16626	7416D00	FESS - post operative removal of polyps (local anaesthetic)
	17173	H135.00	Recurrent sinusitis
	18083	7416C00	FESS - post operative suction clearance (local anaesthetic)
	18869	7416900	FESS/Antrostomy via middle meatus
	19742	H11y.11	Nasal sinus polyps
	20806	7415.11	FESS - diagnostic nasal antroscopy
	20832	7416500	FESS/Anterior ethmoidectomy
	21213	7412700	Radical frontal sinus antrostomy
	21923	7416.11	FESS/Therapeutic nasal antroscopy
	27869	7412800	Frontal sinusotomy NEC
	30990	S832300	Open wound of nasal sinus
	33709	7415z00	FESS/Diagnostic endoscopy of nose or sinus NOS
	33922	7416700	FESS/Anterior and posterior ethmoidectomy
	34165	7412300	Transantral ethmoidectomy
	34973	7416z00	FESS - therapeutic endoscopy of nose or sinus NOS
	35389	7416B00	FESS/Excisional surgery to middle turbinate
	35897	H11y200	Polyp of maxillary sinus
	37481	7416800	FESS/Sphenoidectomy
	39501	H13y000	Chronic pansinusitis
	42166	7416F00	Functional endoscopic sinus surg - polypectomy nasal sinus
	45995	7416y00	FESS - therapeutic endoscopy of nose or sinus OS
	46336	7416A00	FESS/Antrostomy via inferior meatus
	48703	H133.00	Chronic sphenoidal sinusitis
	49348	7416600	FESS/Anterior ethmoidectomy and frontal recess dissection
	49548	H13y.00	Other chronic sinusitis
	54375	H13yz00	Other chronic sinusitis NOS
	59019	7416400	FESS/Uncinectomy and excision of bulla
	59339	7415200	FESS - diagnostic antroscopy via middle meatus
	61281	H11y300	Polyp of sphenoidal sinus
	62936	7416200	FESS/Therapeutic antroscopy via middle meatus
	63733	Hyu2200	[X]Other chronic sinusitis
	64359	7412y00	Other specified operation on frontal sinus
	68003	7M1B000	Functional endoscopic sinus surgery
	69714	7406700	Nasal polypectomy using auto-debrider
	86064	7M1B100	Functional endoscopic nasal surgery
	**OPCS code**		
**OPCS**	E081		Polypectomy of internal nose
	E133		Intranasal antrostomy
	E142		Intranasal ethmoidectomy
	Y761		Functional endoscopic sinus surgery
	Y762		Functional endoscopic nasal surgery

For peer review only - http://bmjopen.bmj.com/site/about/guidelines.xhtml

CRS surgery terms – definite or very likely CRS surgery

The subgroup of surgical patients were those who had also undergone surgery [illegible] second table below.

Code type	Code	Description ("very likely" CRS surgery)
OPCS	E13	Other operations on maxillary antrum
	E131	Drainage of maxillary antrum NEC
	E132	Excision of lesion of maxillary antrum
	E136	Puncture of maxillary antrum
	E138	Other specified other operations on maxillary antrum
	E139	Unspecified other operations on maxillary antrum
	E14	Operations on frontal sinus
	E144	Transantral ethmoidectomy
	E147	Median drainage of frontal sinus
	E148	Other specified operations on frontal sinus
	E149	Unspecified operations on frontal sinus
	E15	Operations on sphenoid sinus
	E151	Drainage of sphenoid sinus
	E152	Puncture of sphenoid sinus
	E158	Other specified operations on sphenoid sinus
	E159	Unspecified operations on sphenoid sinus
	E16	Other operations on frontal sinus
	E161	Frontal sinus osteoplasty
	E162	Drainage of frontal sinus NEC
	E168	Other specified other operations on frontal sinus
	E169	Unspecified other operations on frontal sinus

For peer review only

For peer review only - http://bmjopen.bmj.com/site/about/guidelines.xhtml

Polyp diagnostic terms

The codelist for determining whether a patient was categorised as being in the positive-polyps subgroup or the unknown-polyps subgroup is below. Those patients exhibiting one or more of these [illegible]

Code type	Code	Description ("positive polyps")
	J33	Nasal polyp
	J330	Polyp of nasal cavity
ICD-10	J331	Polypoid sinus degeneration
	J338	Other polyp of sinus
	J339	Nasal polyp, unspecified
	11744	Polyp of ethmoidal sinus
	14749	Polyp of nasal cavity NOS
	14888	Nasal polyp NOS
	16626	FESS - post operative removal of polyps (local anaesthetic)
	19742	Nasal sinus polyps
	24535	Removal of antrochoanal polyp
	35897	Polyp of maxillary sinus
	42166	Functional endoscopic sinus surg - polypectomy nasal sinus
MEDCODE	4341	Antral (maxillary) polyp
	44518	Other polyp of sinus NOS
	44934	Polypoid sinus degeneration
	4686	Nasal polyps
	50528	Polypoid sinus degeneration NOS
	61281	Polyp of sphenoidal sinus
	6411	O/E - nasal polyp present
	69714	Nasal polypectomy using auto-debrider
	811	Nasal polypectomy
	91482	Polyp of frontal sinus
	977	Polyp of nasal cavity
OPCS	E081	Polypectomy of internal nose

For peer review only

For peer review only - http://bmjopen.bmj.com/site/about/guidelines.xhtml

Section B – Unit costs and other input information

NHS Reference Cost categories (2017-18 prices)	Inpatient (APC) events		Outpatient (OP) events	
	Elective Inpatient unit cost (£)	Day Case unit cost (£)	Outpatient unit cost (£)	OPCS/ICD-10 code(s)
Complex sinus procedure	£3,972.25	£2,383.70	£114.23	E147, E152, E153
Major Sinus Procedures	£2,918.76	£2,298.06	£118.52	E131, E132, E141, E143, E144, E146, E148, E149, E151, E154, E158, E159, E161, E162, E168, E169, E171, E172
Intermediate Sinus Procedures	£2,536.77	£1,920.27	£145.73	E133, E138, E139, E142, E178, E179, Y403, Y761, Y762, Z238, Z239
Minor Sinus Procedures	£2,320.64	£1,654.96	£113.14	E136
Intermediate Nose Procedures	£2,241.99	£1,128.15	£164.98	E058, E059, E088, E089, E108, E109, Y408, Y409, Z228, Z229
Nasal Polypectomy	£2,193.28	£1,587.09	£130.83	E081
Minor Nose Procedures	£1,876.76	£1,325.83	£148.90	E061, E062, E063, E064, E068, E069
Excision or Biopsy, of Lesion of Internal Nose	£1,748.75	£1,054.22	£144.62	E082, E101, E134
Other Specified Diagnostic Imaging of Other Sites, 19 years and over	£214.29	£214.29	£214.29	U064, U068, U069, U217
Computerised Tomography Scan of One Area, without Contrast, 19 years and over	£88.21	£88.21	£88.21	U051, U061, U212

Table B1. Unit costs, inpatient and outpatient health care contacts, from published NHS Reference Costs *[23]*. **Prices in 2017-2018 £.**

For peer review only - http://bmjopen.bmj.com/site/about/guidelines.xhtml

Mean duration of visit, minutes	GP	Nurse/Other	References
Standard consultation visit	9.22	9.22	[24]
Specific clinic or home visit	17.20	17.20	[25]
Telephone call	7.10	7.10	[25]
**Unit cost per visit**			
Standard consultation visit	£37.34	£6.45	[24]
Specific clinic or home visit	£69.66	£12.04	[24]
Telephone call	£28.76	£4.97	[24]

Table B2. Unit costs, primary care consultations. Including information on the duration of a contact, and the unit cost calculated on that basis. Prices in 2017-2018 £.

Antibiotics	Unit cost
Cephalosporin	£4.97
Macrolide	£6.64
Metronidazole	£2.13
Penicillin	£1.42
Quinolone	£2.45
Tetracycline	£8.57
Other antibiotic	£4.36
**Non-antibiotics**	**Unit cost**
Corticosteroids	£8.08
All other drugs	£4.69

Table B3. Unit costs of primary care medications, using the British National Formulary (BNF) online *[26]*. **Prices in 2017-2018 £.**

For peer review only

Section C – Numbers of patients "at risk" of a primary care, outpatient, or inpatient care event, centred on surgery date at midpoint of Q0

Years from surgery	-10	-8	-6	-4	-2	0	2	4	6	8	10
Quarters from surgery	-40	-32	-24	-16	-8	0	8	16	24	32	40
**Numbers "at risk" of primary care consultation/prescription event**											
**Positive polyps**	1551	2489	3606	4995	6697	9056	7028	5443	4109	2896	1897
**Unknown polyps**	812	1250	1773	2451	3289	4406	3199	2365	1689	1182	783
**All patients**	2363	3739	5379	7446	9986	13462	10227	7808	5798	4078	2680
**Numbers "at risk" of inpatient care event**											
**Positive polyps**	1451	2362	3434	4805	6461	8691	6683	5219	4035	2923	2007
**Unknown polyps**	768	1198	1717	2360	3163	4207	3226	2397	1738	1245	833
**All patients**	2219	3560	5151	7165	9624	12898	9909	7616	5773	4168	2840
**Numbers "at risk" of outpatient care event**											
**Positive polyps**	0	0	0	0	1135	6433	2962	9	0	0	0
**Unknown polyps**	0	0	0	0	633	3238	1234	5	0	0	0
**All patients**	0	0	0	0	1768	9671	4196	14	0	0	0

Table C1. Numbers of patients "at risk" of a primary care, outpatient, or inpatient care event, centred on surgery date

For peer review only - http://bmjopen.bmj.com/site/about/guidelines.xhtml

Section D – Mean inpatient costs per person-quarter, centred on surgery date at midpoint of Q0

Polypectomy costs were not split into DC and EL categories according to the length of stay as that regression did not converge, so instead a weighted cost was used according to the proportion of DC and EL polypectomies (32.8% DC and 67.2% EL).

Quarters after surgery (surgery date is at centre of Q0)	CT/other imaging, DC	Minor nose incl. biopsy, DC	Int nose and Minor sinus, DC	Int/Major/Complex sinus, DC	CT/other imaging, EL	Minor nose incl. biopsy, EL	Int nose and Minor sinus, EL	Int/Major/Complex sinus, EL	Polypectomy	TOTAL
**-8**	0.00	0.35	0.00	0.61	0.08	0.57	0.00	0.00	0.00	**1.62**
**-7**	0.03	0.00	0.00	0.00	0.08	0.00	0.00	1.56	0.00	**1.66**
**-6**	0.08	0.00	0.00	0.00	0.08	0.00	0.67	0.00	0.00	**0.82**
**-5**	0.07	0.32	0.00	1.10	0.27	2.07	0.65	1.46	0.00	**5.93**
**-4**	0.14	0.00	0.42	0.53	0.21	0.50	0.00	0.70	0.00	**2.52**
**-3**	0.05	0.00	0.41	0.00	0.23	0.96	0.00	0.68	0.00	**2.33**
**-2**	0.04	0.28	0.00	0.99	0.13	0.46	0.00	3.28	0.00	**5.20**
**-1**	0.02	0.82	0.38	0.48	0.34	0.89	0.00	3.79	0.00	**6.73**
**0**	**0.16**	**8.62**	**43.86**	**243.32**	**0.86**	**29.10**	**110.97**	**680.49**	**0.00**	**1117.37**
**1**	0.00	0.00	0.38	3.36	0.08	0.89	1.11	5.74	0.00	**11.56**
**2**	0.00	0.28	1.17	1.49	0.02	0.00	0.00	3.96	0.00	**6.92**
**3**	0.00	0.29	1.22	1.54	0.02	0.00	0.60	4.10	0.00	**7.77**
**4**	0.00	0.30	0.42	1.59	0.02	0.00	1.84	9.86	0.00	**14.04**
**5**	0.00	0.63	0.87	1.65	0.02	0.00	0.64	1.46	0.00	**5.28**
**6**	0.00	0.00	0.45	1.71	0.03	0.53	0.66	3.78	0.00	**7.15**
**7**	0.00	0.00	1.87	2.36	0.03	0.55	0.69	5.49	0.00	**10.98**
**8**	0.00	0.00	0.48	1.22	0.03	0.57	0.71	2.44	0.00	**5.45**

Table D1. Mean inpatient costs (Admittee Patient Care dataset from Hospital Episode Statistics) per person-quarter (2017-2018 £) - polyps unknown. DC = Day Case; EL = Elective Inpatient; CT = computed tomography; Int = intermediate

For peer review only

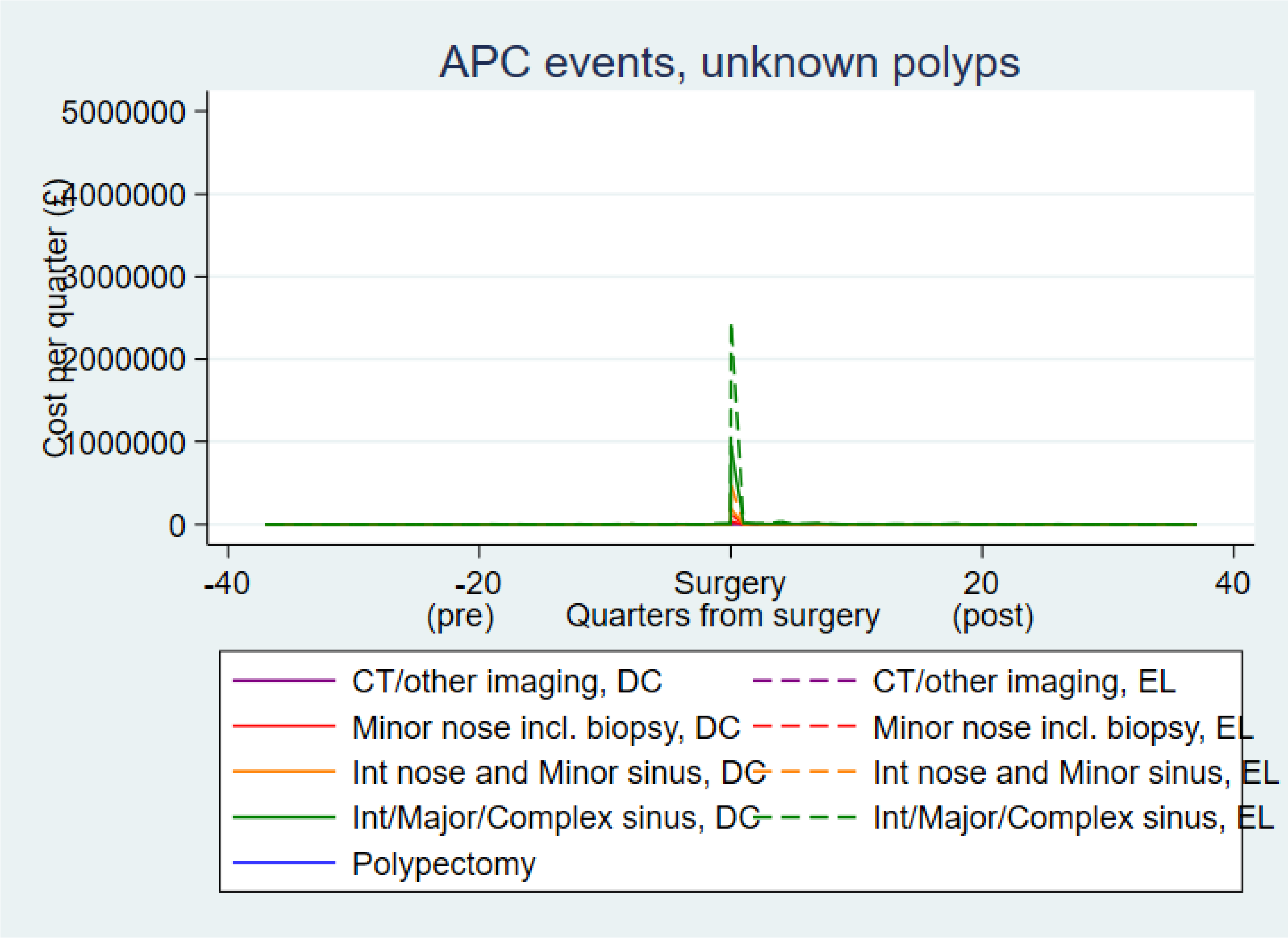
APC events, unknown polyps
Cost per quarter (£)
5000000
4000000
3000000
2000000
1000000
0
-40
-20
(pre)
Surgery
Quarters from surgery
20
(post)
40
CT/other imaging, DC
CT/other imaging, EL
Minor nose incl. biopsy, DC
Minor nose incl. biopsy, EL
Int nose and Minor sinus, DC
Int nose and Minor sinus, EL
Int/Major/Complex sinus, DC
Int/Major/Complex sinus, EL
Polypectomy

Quarters after surgery (surgery date is at centre of Q0)	CT/other imaging, DC	Minor nose incl. biopsy, DC	Int nose and Minor sinus, DC	Int/Major/Complex sinus, DC	CT/other imaging, EL	Minor nose incl. biopsy, EL	Int nose and Minor sinus, EL	Int/Major/Complex sinus, EL	Polypectomy	TOTAL
**-8**	0.00	0.00	0.00	0.30	0.09	0.00	0.00	0.00	0.00	**0.39**
**-7**	0.05	0.00	0.00	0.29	0.05	0.27	0.00	1.15	0.00	**1.81**
**-6**	0.05	0.00	0.00	0.00	0.11	0.26	0.33	0.00	0.00	**0.75**
**-5**	0.02	0.15	0.21	0.00	0.08	0.00	0.00	0.36	0.00	**0.83**
**-4**	0.05	0.45	0.00	0.26	0.15	0.49	0.00	0.34	0.00	**1.73**
**-3**	0.06	0.00	0.00	0.00	0.11	0.47	0.00	0.33	0.25	**1.21**
**-2**	0.05	0.00	0.00	0.00	0.13	0.90	0.56	0.32	0.00	**1.96**
**-1**	0.07	0.13	0.37	0.23	0.11	1.30	0.27	0.61	0.47	**3.57**
**0**	**0.02**	**7.85**	**40.09**	**152.60**	**0.14**	**28.44**	**166.57**	**474.22**	**1414.69**	**2284.63**
**1**	0.00	0.39	0.72	1.16	0.02	0.64	1.06	4.37	4.13	**12.49**
**2**	0.00	0.13	0.56	3.34	0.01	0.22	0.55	3.85	7.54	**16.20**
**3**	0.01	0.69	0.96	2.45	0.02	1.13	1.97	7.26	11.16	**25.63**
**4**	0.01	0.28	0.98	2.76	0.02	0.46	0.58	6.09	9.96	**21.15**
**5**	0.00	0.29	0.61	2.07	0.01	0.48	2.97	6.26	13.58	**26.26**
**6**	0.00	0.15	0.00	2.39	0.00	0.49	1.22	5.01	8.18	**17.45**
**7**	0.00	0.15	0.85	2.19	0.01	0.25	1.26	6.61	12.20	**23.53**
**8**	0.00	0.16	1.10	2.53	0.02	0.26	2.27	4.54	12.02	**22.91**

Table D2. Mean inpatient costs (Admittee Patient Care dataset from Hospital Episode Statistics) per person-quarter (2017-2018 £) - polyps positive. DC = Day Case; EL = Elective Inpatient; CT = computed tomography; Int = intermediate

For peer review only - http://bmjopen.bmj.com/site/about/guidelines.xhtml

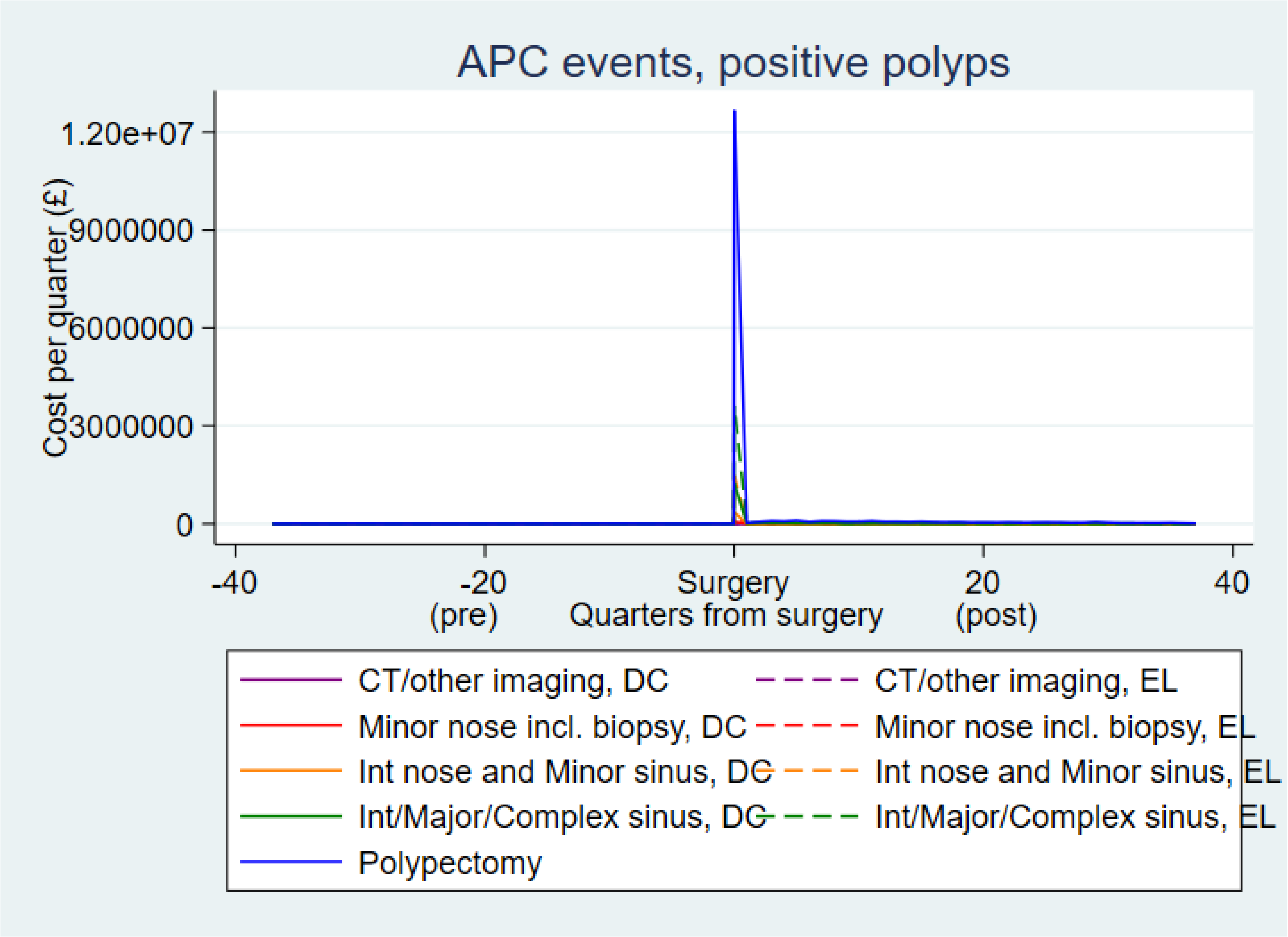

For peer review only - http://bmjopen.bmj.com/site/about/guidelines.xhtml

Quarters after surgery (surgery date is at centre of Q0)	CT/other imaging, DC	Minor nose incl. biopsy, DC	Int nose and Minor sinus, DC	Int/Major/Complex sinus, DC	CT/other imaging, EL	Minor nose incl. biopsy, EL	Int nose and Minor sinus, EL	Int/Major/Complex sinus, EL	Polypectomy	TOTAL
**-8**	0.00	0.12	0.00	0.40	0.09	0.19	0.00	0.00	0.00	**0.80**
**-7**	0.04	0.00	0.00	0.19	0.06	0.18	0.00	1.28	0.00	**1.76**
**-6**	0.06	0.00	0.00	0.00	0.10	0.18	0.44	0.00	0.00	**0.77**
**-5**	0.04	0.21	0.14	0.36	0.15	0.68	0.21	0.72	0.00	**2.51**
**-4**	0.08	0.30	0.14	0.35	0.17	0.49	0.00	0.46	0.00	**1.99**
**-3**	0.05	0.00	0.13	0.00	0.15	0.63	0.00	0.44	0.17	**1.58**
**-2**	0.05	0.09	0.00	0.32	0.13	0.76	0.38	1.28	0.00	**3.02**
**-1**	0.06	0.36	0.37	0.31	0.19	1.17	0.18	1.65	0.31	**4.60**
**0**	**0.07**	**8.10**	**41.32**	**181.87**	**0.37**	**28.66**	**148.42**	**540.56**	**952.62**	**1902.00**
**1**	0.00	0.26	0.61	1.88	0.04	0.72	1.08	4.82	2.79	**12.20**
**2**	0.00	0.18	0.76	2.74	0.01	0.15	0.37	3.88	5.11	**13.20**
**3**	0.01	0.56	1.04	2.16	0.02	0.76	1.53	6.23	7.58	**19.89**
**4**	0.01	0.29	0.80	2.39	0.02	0.31	0.98	7.31	6.78	**18.89**
**5**	0.00	0.40	0.69	1.94	0.02	0.32	2.23	4.71	9.27	**19.58**
**6**	0.00	0.10	0.14	2.18	0.01	0.50	1.05	4.61	5.59	**14.18**
**7**	0.00	0.11	1.17	2.24	0.02	0.35	1.08	6.25	8.37	**19.58**
**8**	0.00	0.11	0.91	2.12	0.03	0.36	1.78	3.87	8.26	**17.43**

Table D3. Mean inpatient costs (Admittee Patient Care dataset from Hospital Episode Statistics) per person-quarter (2017-2018 £) – all polyps. DC = Day Case; EL = Elective Inpatient; CT = computed tomography; Int = intermediate

For peer review only - http://bmjopen.bmj.com/site/about/guidelines.xhtml

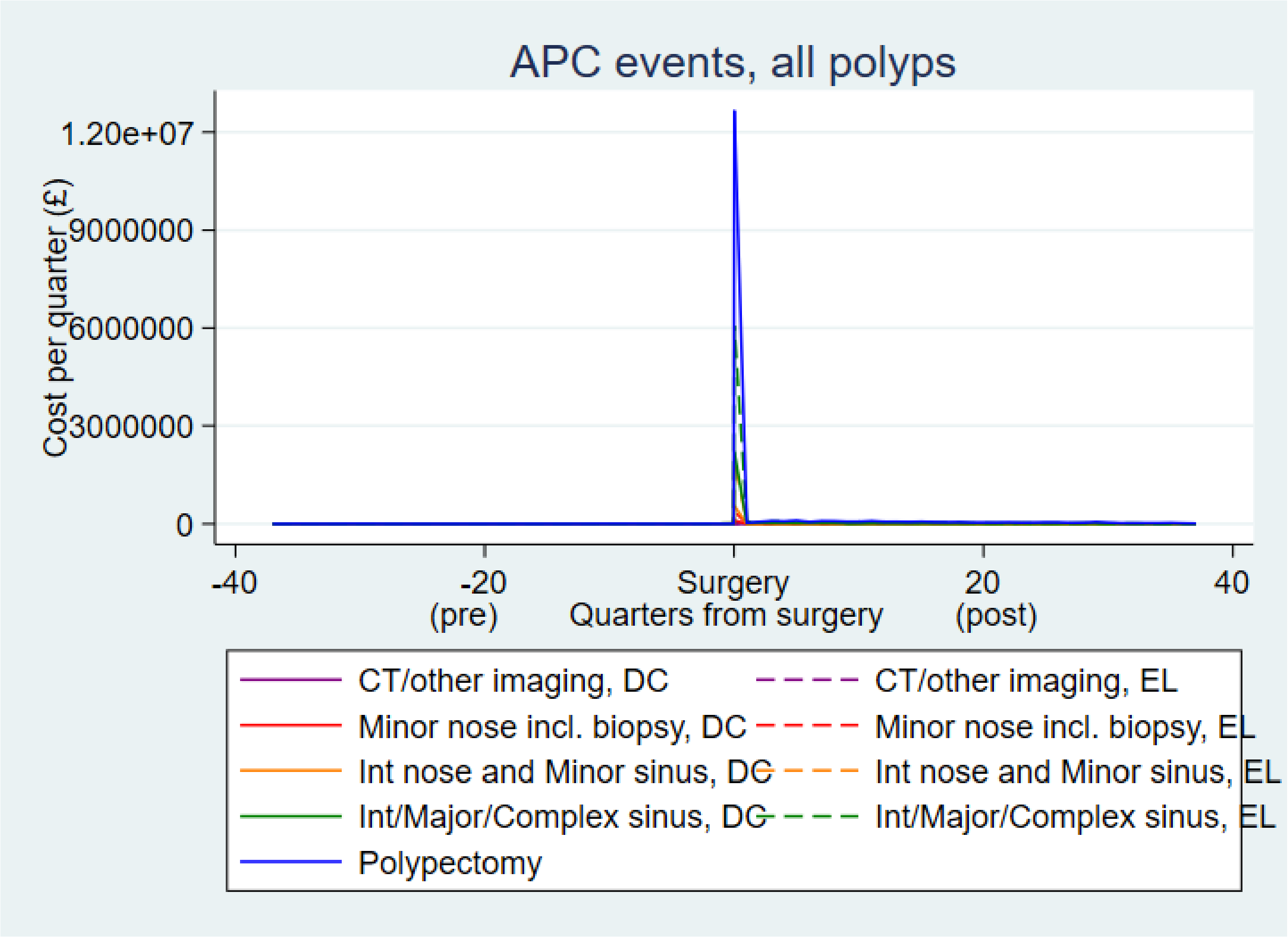
APC events, all polyps
Cost per quarter (£)
1.20e+07
9000000
6000000
3000000
0
-40
-20
(pre)
Surgery
Quarters from surgery
20
(post)
40
CT/other imaging, DC
CT/other imaging, EL
Minor nose incl. biopsy, DC
Minor nose incl. biopsy, EL
Int nose and Minor sinus, DC
Int nose and Minor sinus, EL
Int/Major/Complex sinus, DC
Int/Major/Complex sinus, EL
Polypectomy

13

Section E – Mean outpatient costs per person-quarter, centred on surgery date at midpoint of Q0

Quarters after surgery (surgery date is at centre of Q0)	CT/other imaging	Minor nose incl. biopsy	Polypectomy	Int nose and Minor sinus	Int sinus	Major/ complex sinus	TOTAL
**-8**	8.52	0.80	0.08	3.53	3.79	2.98	**19.69**
**-7**	10.60	1.00	0.03	3.84	5.26	3.43	**24.15**
**-6**	8.69	1.14	0.10	4.01	5.15	1.65	**20.74**
**-5**	9.70	1.03	0.05	4.66	5.60	3.16	**24.21**
**-4**	10.23	0.99	0.16	5.16	6.85	1.51	**24.90**
**-3**	13.62	1.22	0.18	6.06	6.81	3.37	**31.25**
**-2**	16.24	1.64	0.08	7.58	8.27	1.84	**35.67**
**-1**	25.04	1.84	0.44	11.36	12.02	6.17	**56.87**
**0**	**23.03**	**2.93**	**0.85**	**12.47**	**14.46**	**8.66**	**62.41**
**1**	14.88	1.63	0.32	7.57	8.64	2.97	**36.00**
**2**	12.05	1.86	0.12	6.30	6.76	2.04	**29.12**
**3**	9.76	1.53	0.17	6.18	5.75	2.09	**25.48**
**4**	9.36	1.18	0.18	5.40	5.83	2.51	**24.46**
**5**	9.66	0.97	0.15	4.94	5.13	2.58	**23.43**
**6**	9.66	1.18	0.12	4.56	5.95	3.04	**24.50**
**7**	8.91	1.23	0.27	3.90	5.41	1.56	**21.28**
**8**	8.76	1.12	0.13	3.86	5.01	1.61	**20.49**

Table E1. Mean outpatient costs (Outpatient Care dataset from Hospital Episode Statistics) per person-quarter (£) - polyps positive.

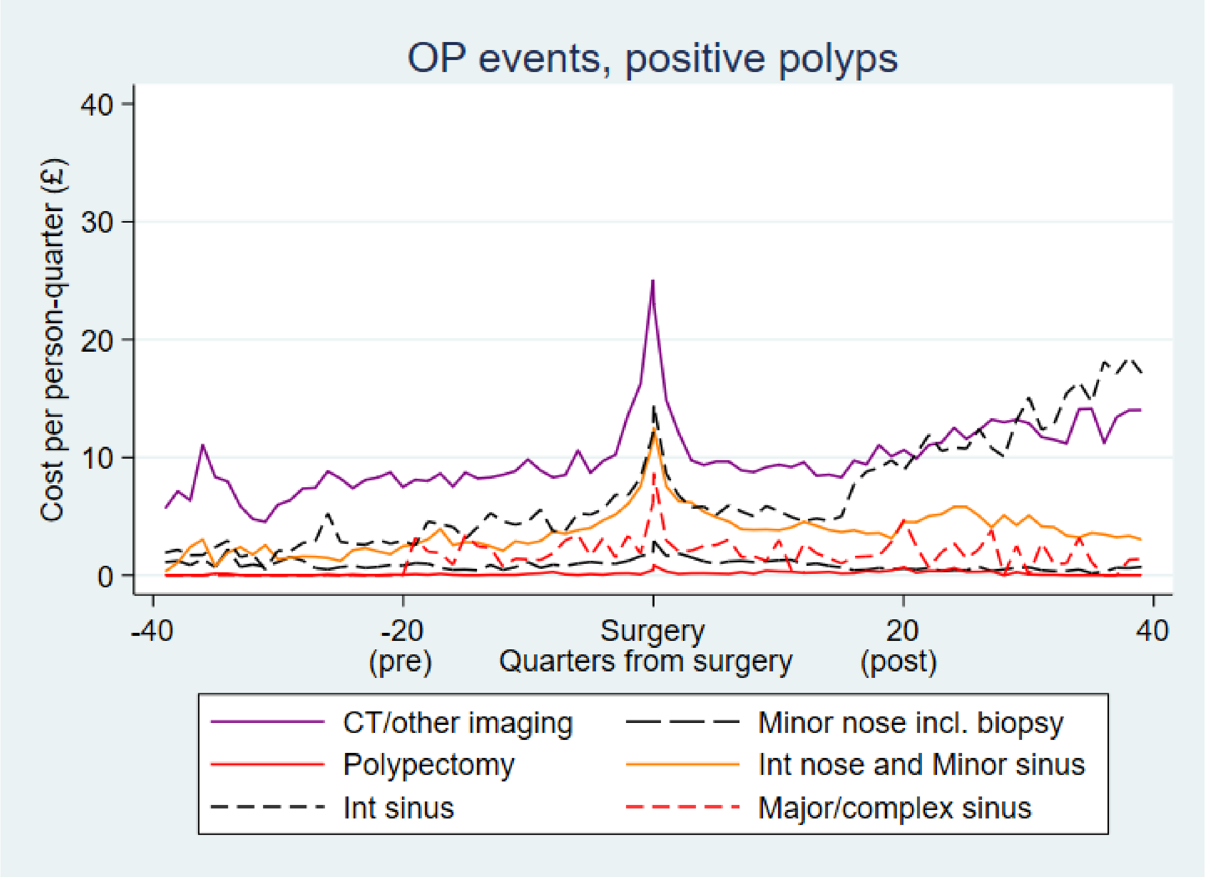

**Quarters after surgery (surgery date is at centre of Q0)**	CT/other imaging	Minor nose incl. biopsy	Polypectomy	Int nose and Minor sinus	Int sinus	Major/ complex sinus	**TOTAL**
**-8**	13.55	2.39	-	5.19	7.41	1.13	**29.67**
**-7**	15.59	3.31	-	4.24	7.94	0.00	**31.07**
**-6**	16.30	2.64	-	5.10	7.48	1.04	**32.57**
**-5**	16.35	2.34	-	5.21	7.07	0.00	**30.97**
**-4**	19.16	2.26	-	5.80	9.08	5.83	**42.14**
**-3**	20.40	2.40	-	7.48	9.41	3.72	**43.42**
**-2**	25.25	2.51	-	9.04	11.10	0.90	**48.79**
**-1**	32.30	4.55	-	11.26	14.70	5.18	**67.99**
**0**	**29.11**	**4.37**	**-**	**13.95**	**17.65**	**10.59**	**75.68**
**1**	25.51	3.16	-	12.17	12.35	5.51	**58.70**
**2**	17.85	2.54	-	9.62	9.16	1.43	**40.59**
**3**	15.27	2.41	-	7.26	8.76	2.22	**35.91**
**4**	15.19	1.93	-	6.57	7.83	2.29	**33.81**
**5**	15.00	2.01	-	6.24	7.46	1.59	**32.30**
**6**	13.02	1.57	-	6.59	6.54	4.92	**32.65**
**7**	14.07	1.32	-	5.68	5.89	9.38	**36.33**
**8**	15.98	1.28	-	5.63	5.87	2.65	**31.40**

Table E2. Mean outpatient costs (Outpatient Care dataset from Hospital Episode Statistics) per person-quarter (£) - polyps unknown.

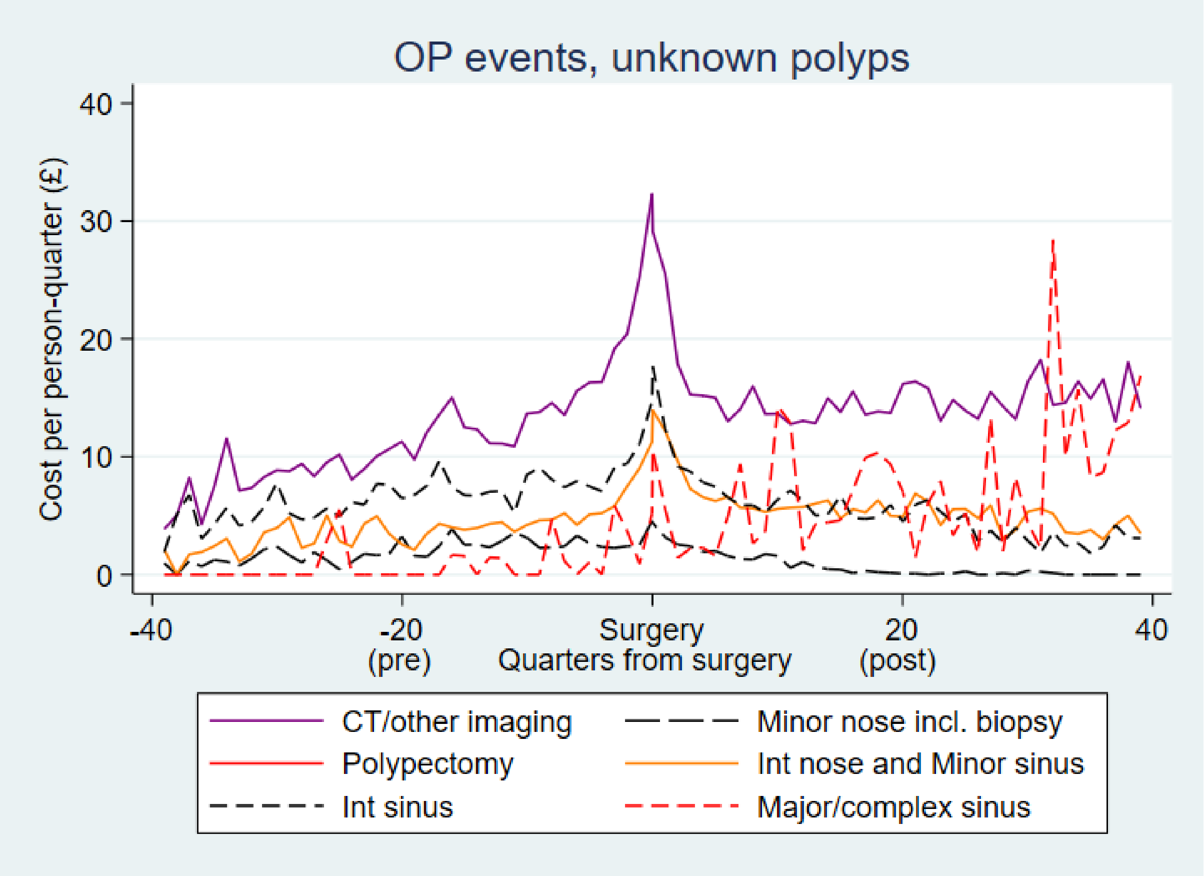

Quarters after surgery (surgery date is at centre of Q0)	CT/other imaging	Minor nose incl. biopsy	Polypectomy	Int nose and Minor sinus	Int sinus	Major/ complex sinus	TOTAL
**-8**	10.25	1.35	0.05	4.10	5.04	2.34	**23.14**
**-7**	12.32	1.80	0.02	3.98	6.18	2.25	**26.53**
**-6**	11.32	1.66	0.07	4.39	5.95	1.44	**24.82**
**-5**	11.98	1.48	0.03	4.85	6.10	2.08	**26.53**
**-4**	13.28	1.42	0.11	5.38	7.61	2.99	**30.78**
**-3**	15.93	1.62	0.12	6.54	7.70	3.49	**35.40**
**-2**	19.29	1.94	0.06	8.07	9.23	1.52	**40.11**
**-1**	27.49	2.76	0.29	11.33	12.93	5.83	**60.62**
**0**	**25.01**	**3.40**	**0.57**	**12.96**	**15.51**	**9.29**	**66.75**
**1**	18.32	2.13	0.21	9.06	9.84	3.79	**43.35**
**2**	13.92	2.08	0.08	7.37	7.53	1.84	**32.82**
**3**	11.52	1.81	0.12	6.52	6.71	2.13	**28.82**
**4**	11.22	1.42	0.12	5.77	6.47	2.44	**27.45**
**5**	11.35	1.30	0.10	5.35	5.87	2.27	**26.24**
**6**	10.72	1.30	0.08	5.21	6.14	3.63	**27.08**
**7**	10.53	1.26	0.18	4.46	5.56	4.01	**26.00**
**8**	11.02	1.17	0.09	4.41	5.28	1.93	**23.91**

Table E3. Mean outpatient costs (Outpatient Care dataset from Hospital Episode Statistics) per person-quarter (£) – all patients.

For peer

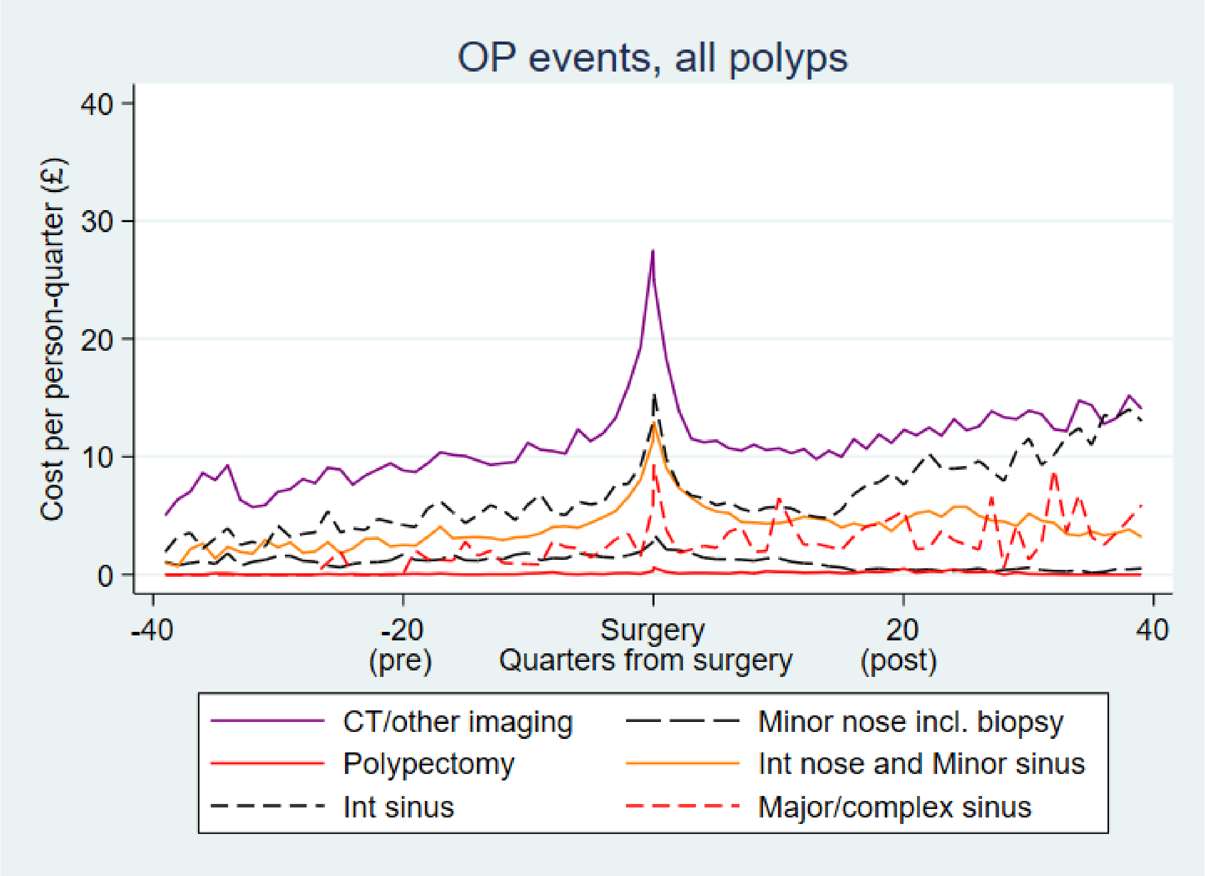

For peer review only - http://bmjopen.bmj.com/site/about/guidelines.xhtml

Section F – Mean primary care consultation costs per person-quarter, centred on surgery date at midpoint of Q0

Quarters after surgery (surgery date is at centre of Q0)	GP consultation	GP home visit	GP telephone	Nurse consultation	Nurse home visit	Nurse telephone	TOTAL
**-8**	7.98	0.03	0.09	0.09	0.01	0.00	**8.20**
**-7**	8.88	0.04	0.10	0.09	0.01	0.00	**9.12**
**-6**	10.45	0.06	0.17	0.13	0.00	0.00	**10.82**
**-5**	12.93	0.04	0.19	0.15	0.01	0.01	**13.31**
**-4**	16.95	0.06	0.21	0.17	0.01	0.01	**17.41**
**-3**	22.30	0.11	0.27	0.26	0.01	0.01	**22.96**
**-2**	27.36	0.11	0.39	0.33	0.02	0.01	**28.23**
**-1**	22.53	0.10	0.44	0.33	0.03	0.01	**23.43**
**0**	**5.36**	**0.01**	**0.12**	**0.09**	**0.01**	**0.00**	**5.59**
**1**	4.63	0.02	0.11	0.07	0.00	0.01	**4.85**
**2**	4.83	0.05	0.13	0.06	0.01	0.00	**5.09**
**3**	4.47	0.01	0.09	0.07	0.01	0.00	**4.65**
**4**	4.43	0.05	0.11	0.07	0.01	0.00	**4.67**
**5**	4.45	0.02	0.13	0.11	0.01	0.00	**4.72**
**6**	4.36	0.04	0.14	0.07	0.01	0.01	**4.62**
**7**	4.32	0.03	0.13	0.06	0.00	0.00	**4.55**
**8**	4.51	0.01	0.14	0.06	0.01	0.00	**4.72**

Table F1. Mean primary care consultation costs (CPRD dataset) per person-quarter (£) - polyps positive.

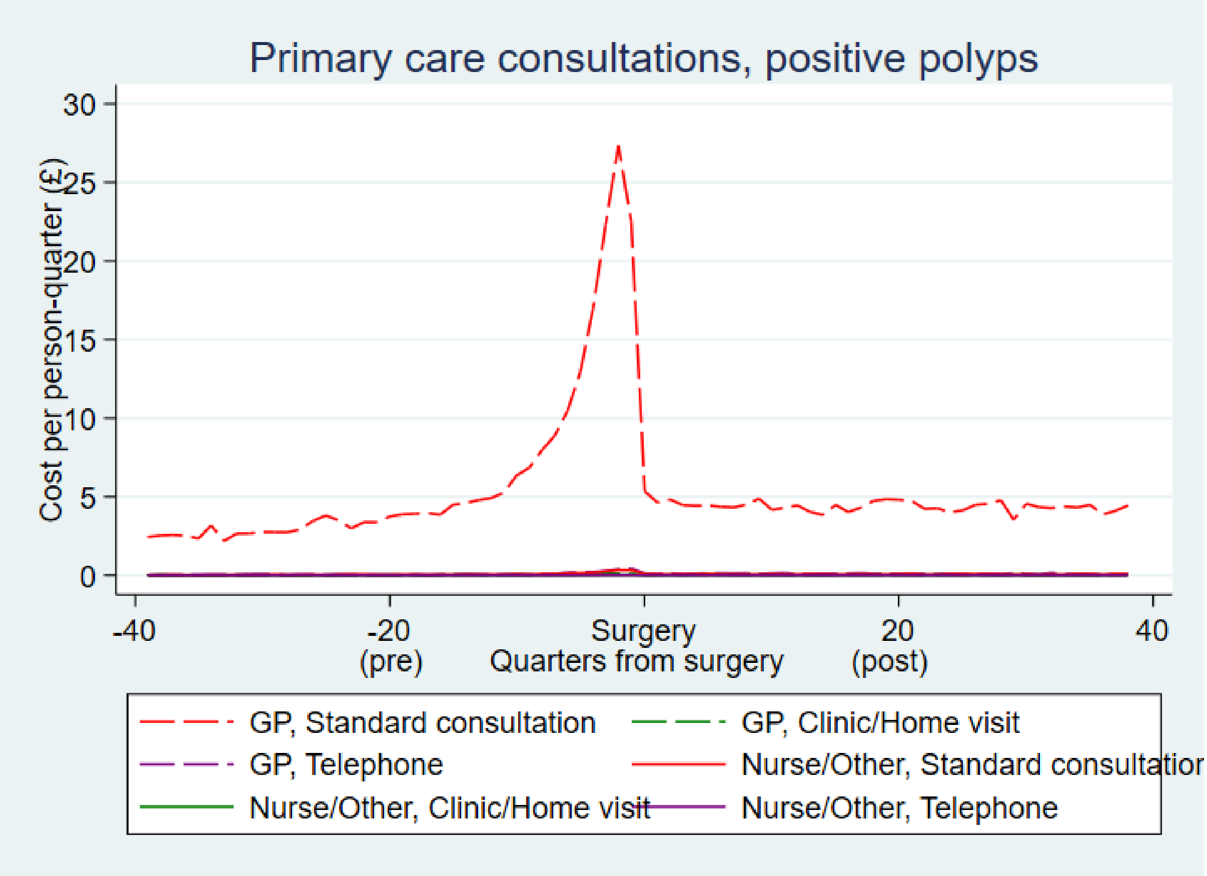

Quarters after surgery (surgery date is at centre of Q0)	GP consultation	GP home visit	GP telephone	Nurse consultation	Nurse home visit	Nurse telephone	TOTAL
**-8**	8.43	0.02	0.11	0.10	0.01	0.00	**8.68**
**-7**	9.64	0.02	0.14	0.13	0.01	0.00	**9.95**
**-6**	12.32	0.03	0.22	0.13	0.01	0.01	**12.72**
**-5**	14.46	0.03	0.24	0.14	0.01	0.00	**14.89**
**-4**	19.41	0.06	0.31	0.22	0.02	0.01	**20.04**
**-3**	21.60	0.08	0.51	0.28	0.02	0.01	**22.50**
**-2**	21.82	0.07	0.50	0.33	0.02	0.01	**22.76**
**-1**	16.23	0.08	0.52	0.25	0.01	0.01	**17.11**
**0**	**6.69**	**0.01**	**0.22**	**0.11**	**0.01**	**0.00**	7.04
**1**	6.77	0.00	0.22	0.12	0.01	0.01	**7.13**
**2**	6.74	0.02	0.09	0.07	0.01	0.01	**6.94**
**3**	5.47	0.02	0.08	0.08	0.00	0.00	**5.66**
**4**	6.57	0.02	0.15	0.07	0.00	0.00	**6.82**
**5**	6.38	0.05	0.12	0.07	0.01	0.00	**6.63**
**6**	5.03	0.03	0.16	0.07	0.01	0.00	**5.31**
**7**	5.85	0.02	0.17	0.05	0.00	0.00	**6.10**
**8**	6.56	0.02	0.17	0.12	0.00	0.00	**6.88**

Table F2. Mean primary care consultation costs (CPRD dataset) per person-quarter (£) - polyps unknown.

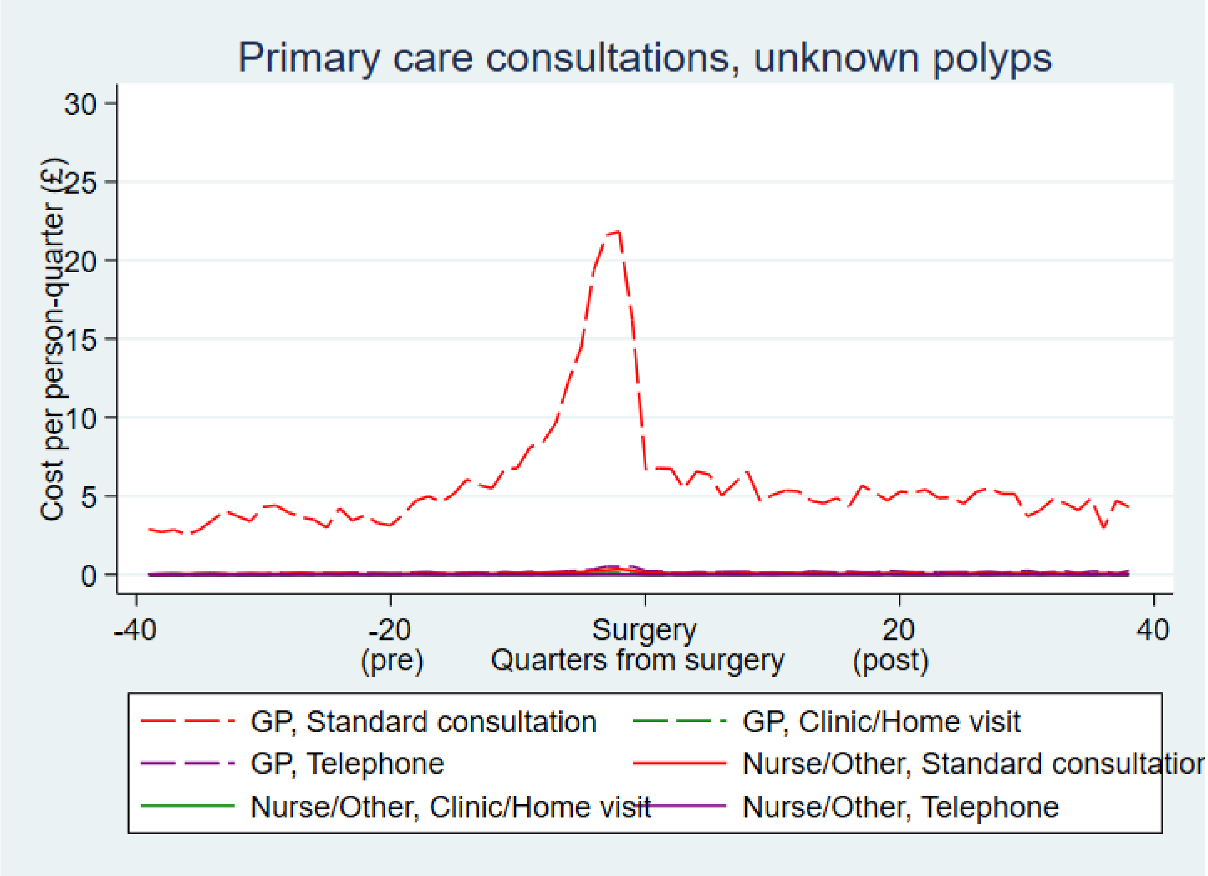

Quarters after surgery (surgery date is at centre of Q0)	GP consultation	GP home visit	GP telephone	Nurse consultation	Nurse home visit	Nurse telephone	TOTAL
**-8**	8.13	0.03	0.10	0.09	0.01	0.00	**8.36**
**-7**	9.13	0.03	0.11	0.10	0.01	0.00	**9.39**
**-6**	11.06	0.05	0.19	0.13	0.01	0.00	**11.45**
**-5**	13.43	0.03	0.20	0.15	0.01	0.01	**13.83**
**-4**	17.75	0.06	0.25	0.19	0.01	0.01	**18.27**
**-3**	22.07	0.10	0.35	0.27	0.02	0.01	**22.81**
**-2**	25.56	0.10	0.42	0.33	0.02	0.01	**26.44**
**-1**	20.47	0.09	0.46	0.31	0.02	0.01	**21.37**
**0**	**5.79**	**0.01**	**0.15**	**0.10**	**0.01**	**0.00**	**6.06**
**1**	5.32	0.01	0.15	0.09	0.01	0.01	**5.58**
**2**	5.44	0.04	0.12	0.07	0.01	0.00	**5.68**
**3**	4.79	0.02	0.09	0.07	0.01	0.00	**4.97**
**4**	5.11	0.04	0.12	0.07	0.00	0.00	**5.35**
**5**	5.06	0.03	0.13	0.10	0.01	0.00	**5.32**
**6**	4.57	0.03	0.14	0.07	0.01	0.01	**4.84**
**7**	4.80	0.03	0.14	0.06	0.00	0.00	**5.04**
**8**	5.15	0.01	0.15	0.07	0.01	0.00	**5.40**

Table F3. Mean primary care consultation costs (CPRD dataset) per person-quarter (£) – all patients.

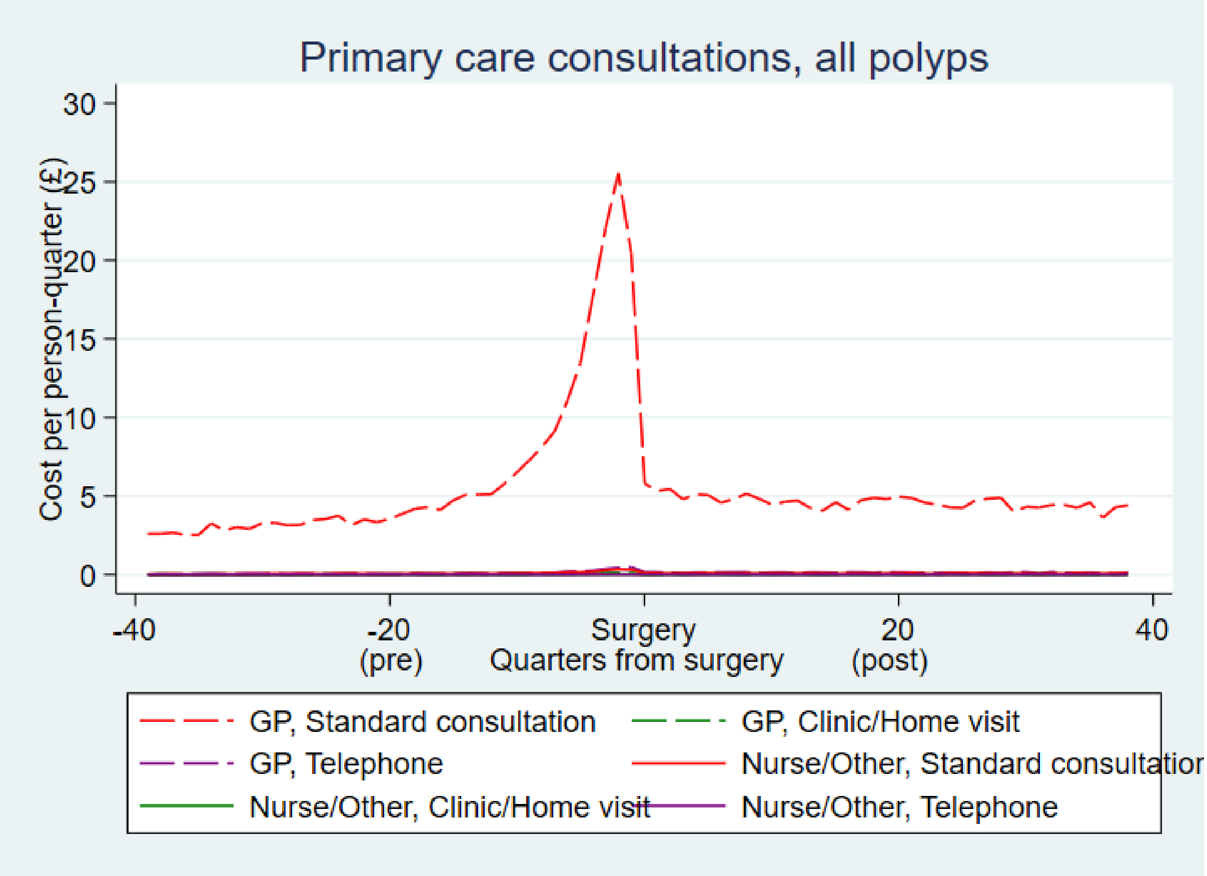

Section G – Mean primary care antibiotic prescription costs per-person-quarter, centred on surgery date at midpoint of Q0

Quarters after surgery (surgery date at centre of Q0)	Cephalo-sporin	Macrolides	Metro-nidazole	Penicillin	Quinolone	Tetra-cycline	Other	TOTAL
**-8**	0.07	0.26	0.01	0.18	0.02	0.30	0.03	**0.87**
**-7**	0.08	0.28	0.01	0.18	0.03	0.34	0.04	**0.97**
**-6**	0.08	0.27	0.01	0.20	0.02	0.36	0.03	**0.98**
**-5**	0.09	0.29	0.01	0.21	0.02	0.42	0.04	**1.08**
**-4**	0.09	0.33	0.01	0.22	0.03	0.44	0.04	**1.15**
**-3**	0.09	0.39	0.01	0.24	0.03	0.57	0.05	**1.37**
**-2**	0.10	0.41	0.01	0.25	0.03	0.63	0.04	**1.46**
**-1**	0.07	0.41	0.01	0.24	0.03	0.51	0.04	**1.32**
**0**	**0.09**	**0.31**	**0.01**	**0.21**	**0.03**	**0.31**	**0.04**	**0.99**
**1**	0.07	0.29	0.01	0.19	0.03	0.28	0.04	**0.92**
**2**	0.07	0.29	0.01	0.20	0.03	0.30	0.04	**0.93**
**3**	0.08	0.26	0.01	0.20	0.03	0.32	0.04	**0.95**
**4**	0.08	0.30	0.01	0.19	0.03	0.32	0.05	**0.97**
**5**	0.07	0.25	0.01	0.20	0.03	0.33	0.04	**0.93**
**6**	0.09	0.29	0.01	0.20	0.03	0.31	0.05	**0.97**
**7**	0.08	0.30	0.01	0.19	0.03	0.30	0.04	**0.94**
**8**	0.08	0.28	0.01	0.20	0.03	0.35	0.04	**0.99**

Table G1. Mean primary care antibiotic prescription costs (CPRD dataset) per person-quarter (£) - polyps positive.

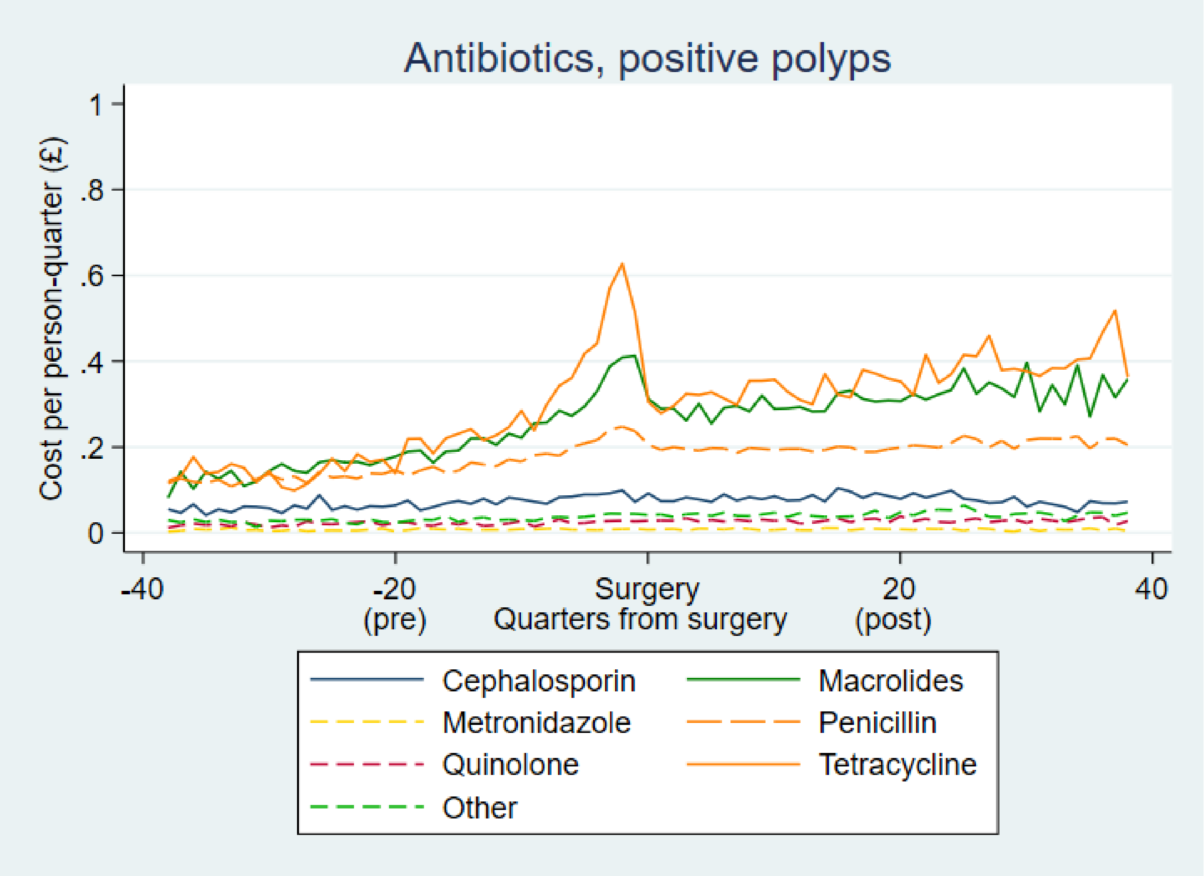

For peer review only - http://bmjopen.bmj.com/site/about/guidelines.xhtml

Quarters after surgery (surgery date at centre of Q0)	Cephalo-sporin	Macrolides	Metro-nidazole	Penicillin	Quinolone	Tetra-cycline	Other	TOTAL
**-8**	0.11	0.29	0.02	0.19	0.02	0.52	0.06	**1.21**
**-7**	0.11	0.32	0.01	0.22	0.02	0.52	0.04	**1.24**
**-6**	0.11	0.43	0.01	0.23	0.03	0.64	0.03	**1.49**
**-5**	0.15	0.42	0.01	0.28	0.03	0.65	0.05	**1.59**
**-4**	0.14	0.52	0.01	0.29	0.04	0.90	0.05	**1.96**
**-3**	0.13	0.62	0.02	0.30	0.04	0.98	0.04	**2.15**
**-2**	0.13	0.60	0.02	0.29	0.04	0.98	0.05	**2.11**
**-1**	0.13	0.54	0.01	0.27	0.03	0.77	0.05	**1.82**
**0**	**0.13**	**0.37**	**0.01**	**0.21**	**0.04**	**0.45**	**0.05**	**1.27**
**1**	0.11	0.35	0.01	0.22	0.04	0.50	0.05	**1.29**
**2**	0.10	0.36	0.02	0.20	0.04	0.50	0.05	**1.26**
**3**	0.10	0.32	0.01	0.19	0.03	0.48	0.04	**1.17**
**4**	0.10	0.35	0.01	0.21	0.03	0.54	0.06	**1.30**
**5**	0.10	0.38	0.01	0.20	0.03	0.47	0.05	**1.24**
**6**	0.08	0.39	0.01	0.18	0.03	0.48	0.05	**1.23**
**7**	0.09	0.36	0.01	0.20	0.03	0.51	0.06	**1.26**
**8**	0.10	0.40	0.02	0.19	0.03	0.52	0.05	**1.30**

Table G2. Mean primary care antibiotic prescription costs (CPRD dataset) per person-quarter (£) - polyps unknown.

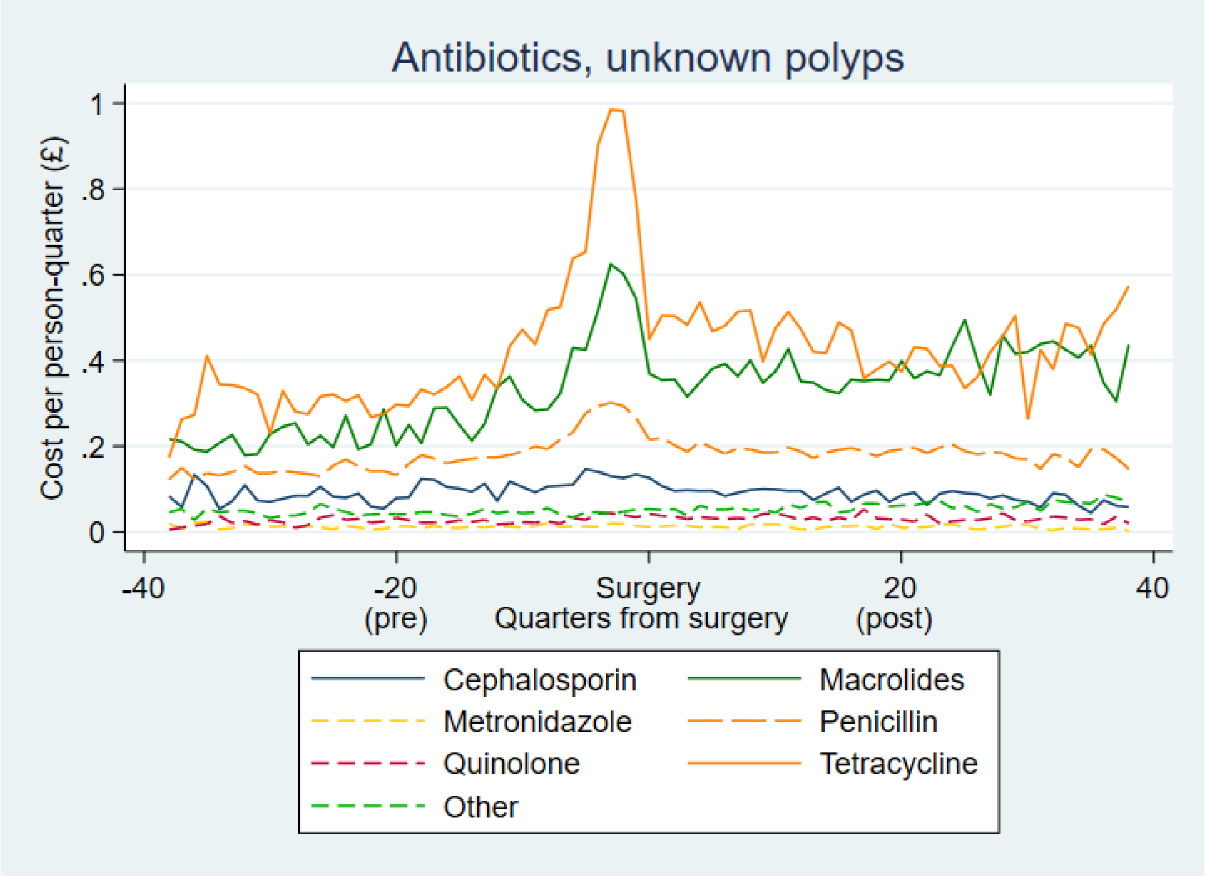

Quarters after surgery (surgery date at centre of Q0)	Cephalo-sporin	Macrolides	Metro-nidazole	Penicillin	Quinolone	Tetra-cycline	Other	TOTAL
**-8**	0.08	0.27	0.01	0.19	0.02	0.37	0.04	**0.98**
**-7**	0.09	0.30	0.01	0.19	0.03	0.40	0.04	**1.06**
**-6**	0.09	0.32	0.01	0.21	0.03	0.45	0.03	**1.15**
**-5**	0.11	0.34	0.01	0.23	0.02	0.50	0.04	**1.25**
**-4**	0.11	0.39	0.01	0.24	0.03	0.59	0.04	**1.41**
**-3**	0.10	0.47	0.01	0.26	0.03	0.71	0.04	**1.62**
**-2**	0.11	0.47	0.01	0.26	0.03	0.74	0.05	**1.68**
**-1**	0.09	0.46	0.01	0.25	0.03	0.60	0.05	**1.48**
**0**	**0.10**	**0.33**	**0.01**	**0.21**	**0.03**	**0.35**	**0.05**	**1.08**
**1**	0.08	0.31	0.01	0.20	0.03	0.35	0.05	**1.04**
**2**	0.08	0.31	0.01	0.20	0.03	0.36	0.04	**1.04**
**3**	0.09	0.28	0.01	0.19	0.03	0.37	0.04	**1.02**
**4**	0.08	0.32	0.01	0.20	0.03	0.39	0.05	**1.08**
**5**	0.08	0.29	0.01	0.20	0.03	0.37	0.04	**1.03**
**6**	0.09	0.32	0.01	0.19	0.03	0.37	0.05	**1.05**
**7**	0.08	0.32	0.01	0.19	0.03	0.37	0.04	**1.04**
**8**	0.09	0.32	0.01	0.20	0.03	0.40	0.04	**1.09**

Table G3. Mean primary care antibiotic prescription costs (CPRD dataset) per person-quarter (£) – all patients.

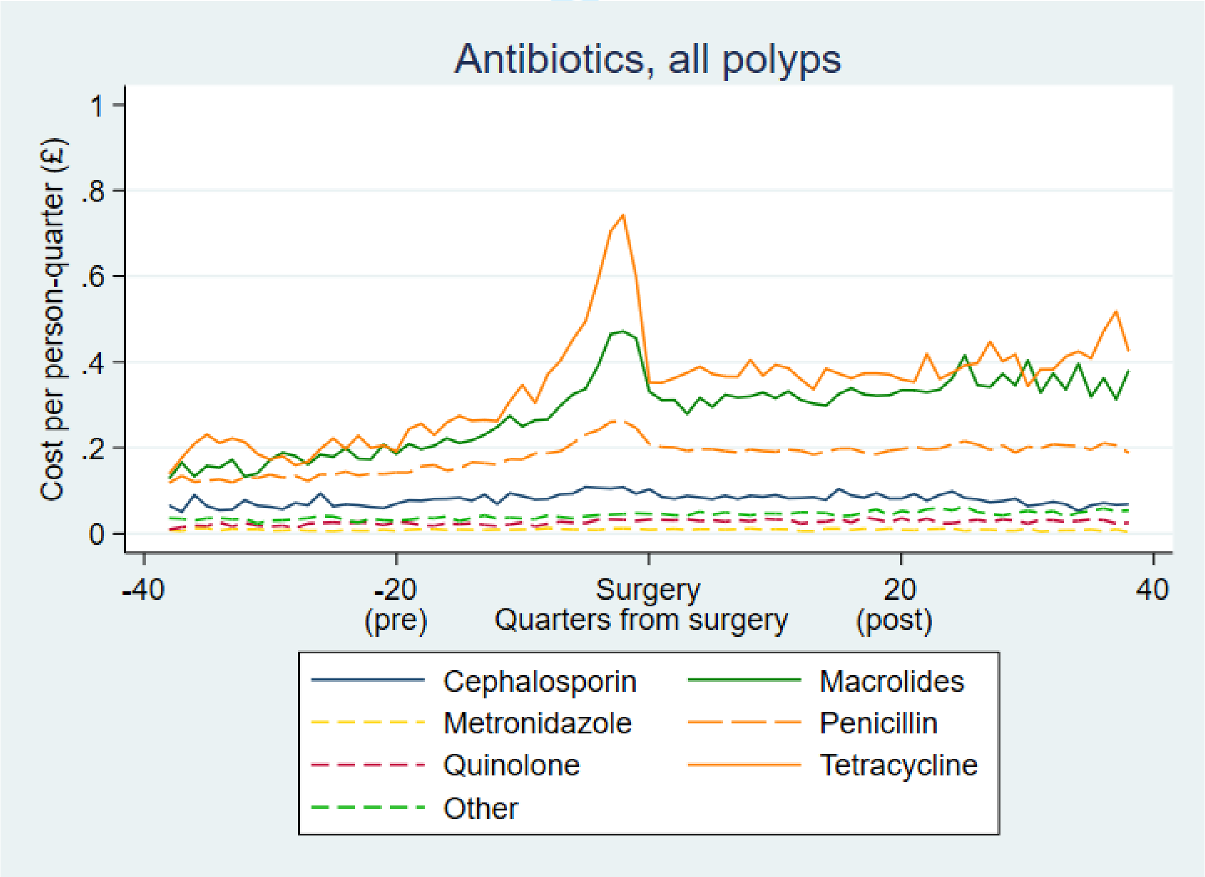

For peer review only - http://bmjopen.bmj.com/site/about/guidelines.xhtml

Section H – Mean primary care non-antibiotic prescription costs per-person-quarter, centred on surgery date at midpoint of Q0

Quarters after surgery (surgery date at centre of Q0)	Steroids	All other non-abx meds	**TOTAL**
**-8**	3.80	2.17	**5.97**
**-7**	3.96	2.24	**6.20**
**-6**	4.35	2.25	**6.60**
**-5**	4.89	2.37	**7.26**
**-4**	5.45	2.43	**7.88**
**-3**	6.12	2.56	**8.68**
**-2**	6.84	2.57	**9.41**
**-1**	6.48	2.64	**9.13**
**0**	**5.33**	**2.46**	**7.79**
**1**	5.15	2.51	**7.66**
**2**	5.12	2.60	**7.72**
**3**	4.90	2.69	**7.59**
**4**	4.81	2.67	**7.49**
**5**	4.86	2.76	**7.62**
**6**	4.90	2.82	**7.73**
**7**	4.83	2.82	**7.65**
**8**	4.83	2.79	**7.62**

Table H1. Mean primary care non-antibiotic prescription costs (CPRD dataset) per person-quarter (£) - polyps positive.

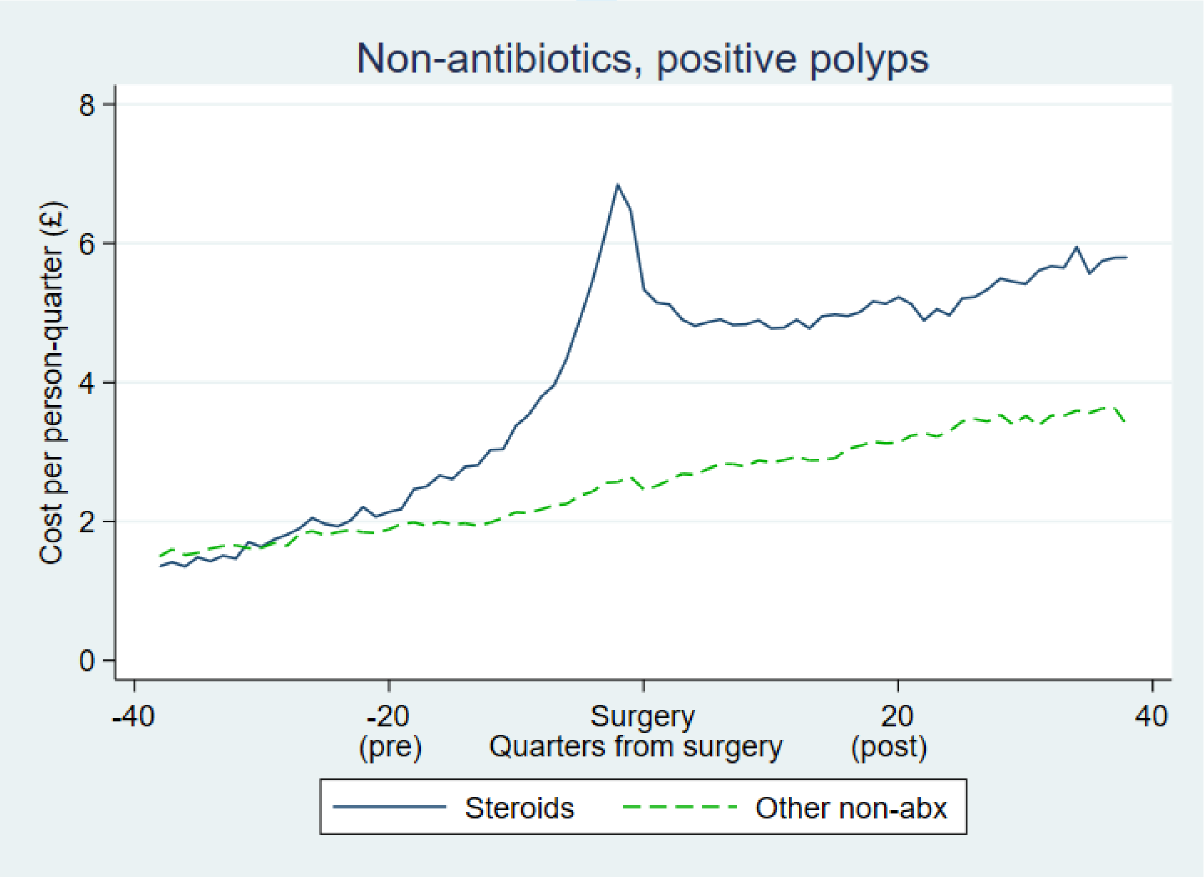

For peer review only - http://bmjopen.bmj.com/site/about/guidelines.xhtml

**Quarters after surgery (surgery date at centre of Q0)**	Steroids	All other non-abx meds	**TOTAL**
**-8**	1.96	2.75	**4.70**
**-7**	2.09	2.81	**4.89**
**-6**	2.43	2.82	**5.25**
**-5**	2.76	2.92	**5.68**
**-4**	3.22	3.03	**6.25**
**-3**	3.61	3.03	**6.64**
**-2**	3.76	3.19	**6.96**
**-1**	3.43	3.13	**6.56**
**0**	**2.62**	**2.93**	**5.54**
**1**	2.63	3.00	**5.63**
**2**	2.47	3.02	**5.49**
**3**	2.34	3.04	**5.39**
**4**	2.50	3.04	**5.54**
**5**	2.43	3.12	**5.55**
**6**	2.40	3.05	**5.45**
**7**	2.31	3.05	**5.36**
**8**	2.38	3.18	**5.56**

Table H2. Mean primary care non-antibiotic prescription costs (CPRD dataset) per person-quarter (£) - polyps unknown.

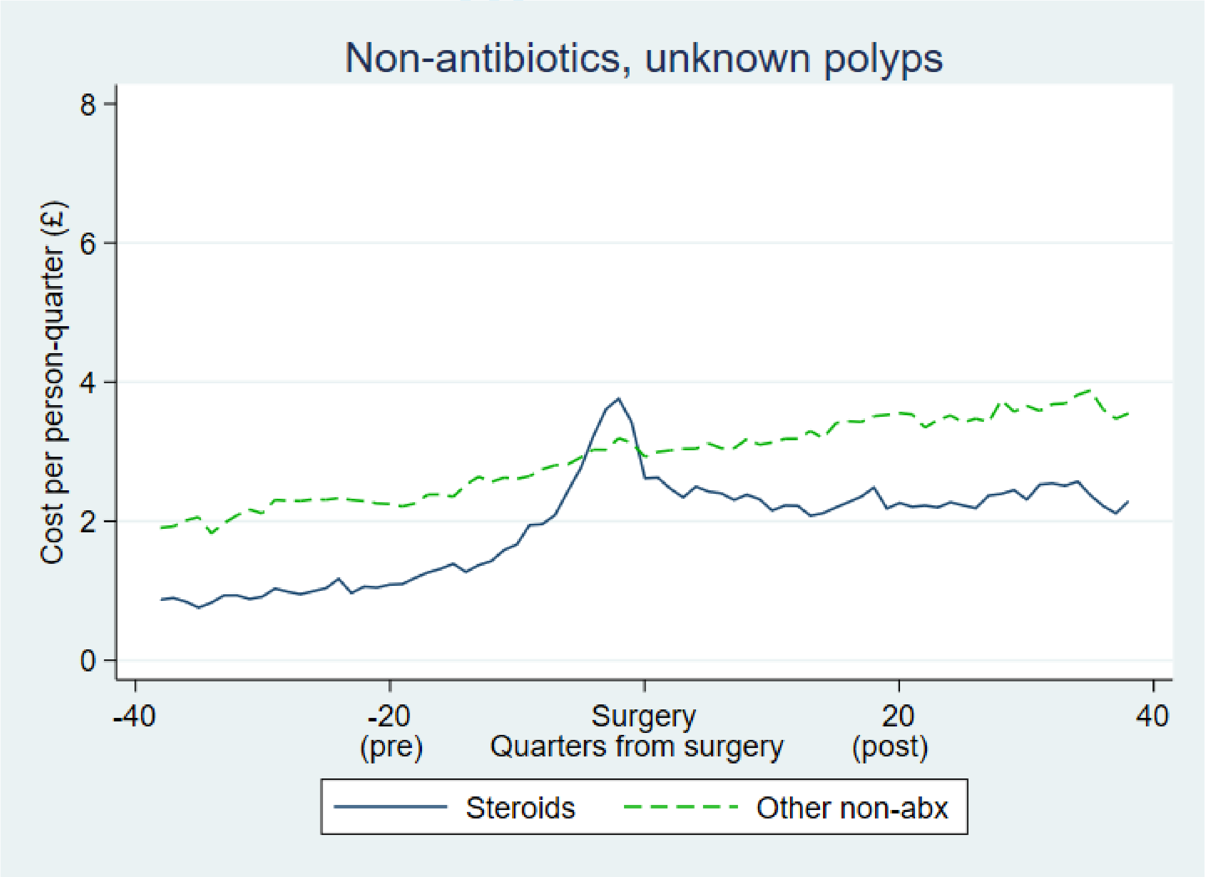

For peer review only - http://bmjopen.bmj.com/site/about/guidelines.xhtml

**Quarters after surgery (surgery date at centre of Q0)**	Steroids	All other non-abx meds	**TOTAL**
**-8**	3.19	2.36	**5.55**
**-7**	3.34	2.42	**5.77**
**-6**	3.72	2.44	**6.15**
**-5**	4.19	2.55	**6.74**
**-4**	4.72	2.63	**7.35**
**-3**	5.30	2.71	**8.01**
**-2**	5.84	2.77	**8.61**
**-1**	5.49	2.80	**8.29**
**0**	**4.45**	**2.61**	**7.06**
**1**	4.34	2.67	**7.00**
**2**	4.27	2.73	**7.00**
**3**	4.09	2.80	**6.89**
**4**	4.08	2.79	**6.87**
**5**	4.09	2.87	**6.96**
**6**	4.12	2.89	**7.01**
**7**	4.04	2.89	**6.93**
**8**	4.07	2.91	**6.98**

Table H3. Mean primary care non-antibiotic prescription costs (CPRD dataset) per person-quarter (£) – all patients.

For peer

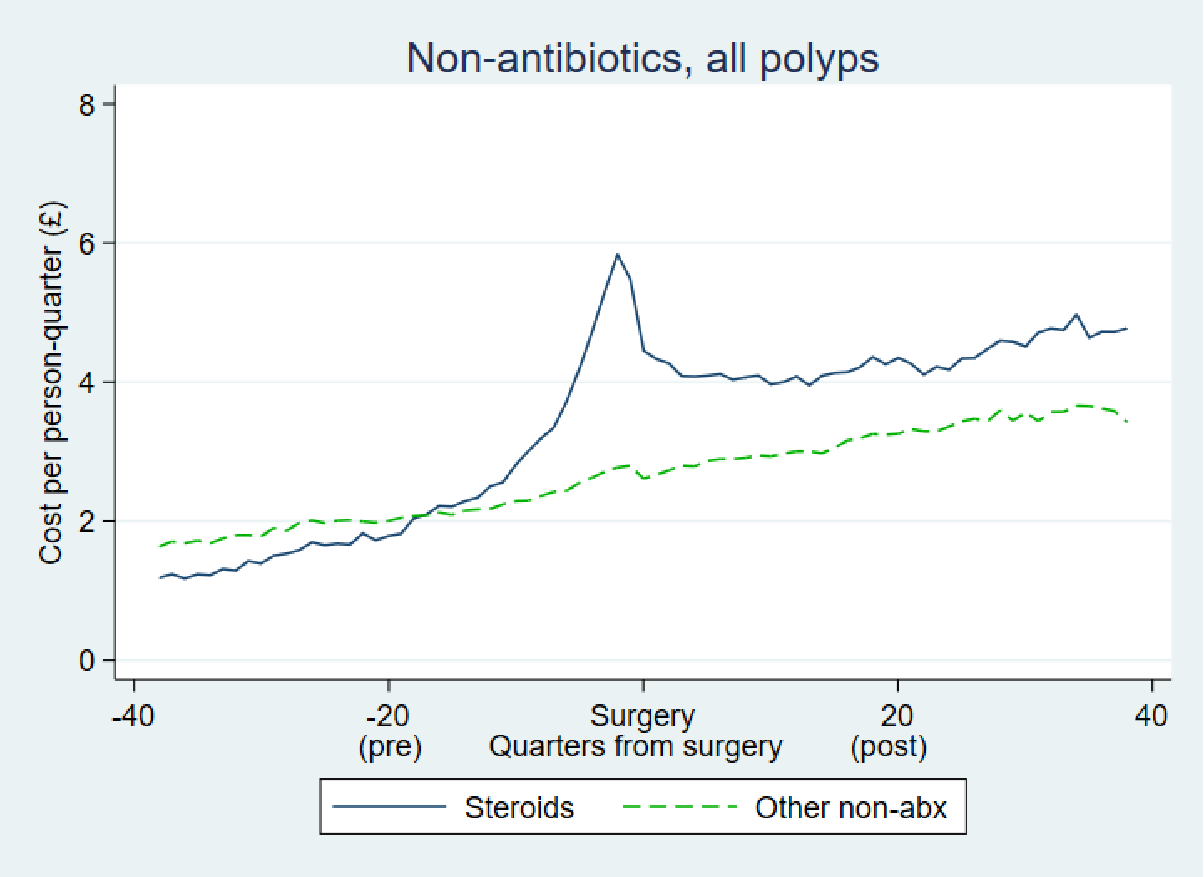

BMJ Open

Observational retrospective study calculating health service costs of patients receiving surgery for chronic rhinosinusitis in England, using linked patient-level primary and secondary care electronic data

Journal:	*BMJ Open*
Manuscript ID	bmjopen-2021-055603.R1
Article Type:	Original research
Date Submitted by the Author:	16-Dec-2021
Complete List of Authors:	Clarke, Caroline; University College London, Research Department of Primary Care and Population Health<br>Williamson, Elizabeth; London School of Hygiene & Tropical Medicine, Department of Medical Statistics; Health Data Research UK<br>Denaxas, S; Health Data Research UK; University College London, Institute of Health Informatics<br>Carpenter, James; London School of Hygiene & Tropical Medicine, Department of Medical Statistics; University College London, MRC Clinical Trials Unit at UCL<br>Thomas, Mike; University of Southampton, Primary Care and Population Science<br>Blackshaw, Helen; ENT Clinical<br>Schilder, Anne; NIHR UCLH Biomedical Research Centre; University College London, Ear Institute<br>Philpott, Carl; University of East Anglia, Norwich Medical School; James Paget University Hospitals NHS Foundation Trust,<br>Hopkins, Claire; Guy's and St Thomas' NHS Foundation Trust, ENT<br>Morris, Stephen; University of Cambridge, Primary Care Unit, Department of Public Health and Primary Care
**Primary Subject Heading**:	Health economics
Secondary Subject Heading:	Ear, nose and throat/otolaryngology, Health services research
Keywords:	HEALTH ECONOMICS, OTOLARYNGOLOGY, Otolaryngology < SURGERY, Clinical trials < THERAPEUTICS

SCHOLARONE™
Manuscripts

I, the Submitting Author has the right to grant and does grant on behalf of all authors of the Work (as defined in the below author licence), an exclusive licence and/or a non-exclusive licence for contributions from authors who are: i) UK Crown employees; ii) where BMJ has agreed a CC-BY licence shall apply, and/or iii) in accordance with the terms applicable for US Federal Government officers or employees acting as part of their official duties; on a worldwide, perpetual, irrevocable, royalty-free basis to BMJ Publishing Group Ltd ("BMJ") its licensees and where the relevant Journal is co-owned by BMJ to the co-owners of the Journal, to publish the Work in this journal and any other BMJ products and to exploit all rights, as set out in our licence.

The Submitting Author accepts and understands that any supply made under these terms is made by BMJ to the Submitting Author unless you are acting as an employee on behalf of your employer or a postgraduate student of an affiliated institution which is paying any applicable article publishing charge ("APC") for Open Access articles. Where the Submitting Author wishes to make the Work available on an Open Access basis (and intends to pay the relevant APC), the terms of reuse of such Open Access shall be governed by a Creative Commons licence – details of these licences and which Creative Commons licence will apply to this Work are set out in our licence referred to above.

Other than as permitted in any relevant BMJ Author's Self Archiving Policies, I confirm this Work has not been accepted for publication elsewhere, is not being considered for publication elsewhere and does not duplicate material already published. I confirm all authors consent to publication of this Work and authorise the granting of this licence.

For peer review only

Title:

Observational retrospective study calculating health service costs of patients receiving surgery for chronic rhinosinusitis in England, using linked patient-level primary and secondary care electronic data

Running head: MACRO healthcare cost CRS surgery

Authors:

Caroline S Clarke[1]* (caroline.clarke@ucl.ac.uk), Elizabeth Williamson[2,3], Spiros Denaxas[3,4], James R Carpenter[2,3,5], Mike Thomas[6], Helen Blackshaw[7], Anne GM Schilder[8,9], Carl Philpott[10,11], Claire Hopkins[12] and Stephen Morris[13], on behalf of the MACRO programme team

Corresponding author: Caroline S Clarke (caroline.clarke@ucl.ac.uk)

Name	Affiliation
Caroline S Clarke[1]	1. Research Department of Primary Care and Population Health, University College London, London, UK.
Elizabeth Williamson[2,3],	2. Department of Medical Statistics, London School of Hygiene & Tropical Medicine, London, UK;<br>3. Health Data Research UK, London, UK
Spiros Denaxas[3,4],	3. Health Data Research UK, London, UK;<br>4. Institute of Health Informatics, University College London, London, UK
James R Carpenter[2,3,5],	2. Department of Medical Statistics, London School of Hygiene & Tropical Medicine, London, UK;<br>3. Health Data Research UK, London, UK;<br>5. MRC Clinical Trials Unit at UCL, London, UK
Mike Thomas[6],	6. Primary Care and Population Science, University of Southampton, UK
Helen Blackshaw[7]	7. ENT Clinical, Zeist, Netherlands
Anne GM Schilder[8,9]	8. NIHR University College London Hospitals Biomedical Research Centre,<br>9. Ear Institute, UCL, London, UK
Carl Philpott[10,11]	10. Norwich Medical School, University of East Anglia, Norwich, UK;<br>11. ENT Department, James Paget University Hospital NHS Foundation Trust, Great Yarmouth, UK
Claire Hopkins[12]	12. ENT Department, Guy's and St Thomas' NHS Foundation Trust, London, UK
Stephen Morris[13]	13. Primary Care Unit, Department of Public Health and Primary Care, University of Cambridge

For peer review only - http://bmjopen.bmj.com/site/about/guidelines.xhtml

Abstract:

Objectives

Chronic rhinosinusitis (CRS) symptoms are experienced by an estimated 11% of UK adults, and symptoms have major impacts on quality of life. Data from UK and elsewhere suggest high economic burden of CRS, but detailed cost information and economic analyses regarding surgical pathway are lacking. This paper estimates healthcare costs for patients receiving surgery for CRS in England.

Design

Observational retrospective study examining cost of healthcare of patients receiving CRS surgery.

Setting

Linked electronic health records from the Clinical Practice Research Datalink, Hospital Episode Statistics and Office for National Statistics databases in England.

Participants

A phenotyping algorithm utilising medical ontology terms identified "definite" CRS cases who received CRS surgery. Patients were registered with a general practice in England. Data covered the period 1997-2016. A cohort of 13,462 patients had received surgery for CRS, with 9,056 (67%) having confirmed nasal polyps.

Outcome measures

Information was extracted on numbers and types of primary care prescriptions and consultations, and inpatient and outpatient hospital investigations and procedures. Resource use was costed using published sources.

Results

Total National Health Service costs in CRS surgery patients were £2,173 over one year including surgery. Total costs per person-quarter were £1,983 in the quarter containing surgery, mostly comprising surgical inpatient care costs (£1,902), and around £60 per person-quarter in the 2 years before and after surgery, of which half were outpatient costs. Outpatient and primary care costs were low compared to the peak in inpatient costs at surgery. The highest outpatient expenditure was on computed tomography scans, peaking in the quarter preceding surgery.

Conclusions

We present the first study of costs to the English healthcare system for patients receiving surgery for CRS. The total aggregate costs provide a further impetus for trials to evaluate the relative benefit of surgical intervention.

For peer review only

Key words:

Cost of health care, chronic rhinosinusitis, observational data, surgery, electronic health records

HEALTH ECONOMICS, OTOLARYNGOLOGY, Otolaryngology < SURGERY, Clinical trials < THERAPEUTICS

Word count

4319/4000 not including tables, figures, legends, references.

Article summary

Strengths and Limitations

- Using linked patient-level primary and secondary healthcare records covering 8% of the England population, we provide a comprehensive picture of the costs to the national healthcare system for chronic rhinosinusitis (CRS) surgical patients undergoing surgery for their CRS
- Our work addresses a paucity of evidence regarding the direct costs of the surgical treatment pathway for CRS in England, and provides a valuable resource to aid commissioning decisions and future research involving surgical treatments for CRS in the UK
- Coding limitations common in observational data mean that the ‘unknown-polyps’ subgroup cannot definitively be stated to contain only CRS patients without polyps (CRSsNP) as some patients with polyps might also be present if their polyps were not recorded in a standard way

For peer review only

For peer review only - http://bmjopen.bmj.com/site/about/guidelines.xhtml

1. Introduction

Chronic rhinosinusitis (CRS) represents a common source of ill health, affecting 5-12% of the general population [1]. In the UK, 11% of adults reported having CRS symptoms [2]. Symptoms, often poorly controlled [3], and including nasal obstruction, nasal discharge, facial pain, anosmia and sleep disturbance, have major impacts on quality of life (QOL), possibly greater than the QOL impacts of chronic respiratory disease or angina [4]. In addition, expenditure on rhinosinusitis treatments has been estimated in the US as higher than for diseases such as ulcer disease, acute asthma and hay fever [5]. The socio-economic cost of CRS is significant with 57% of patients reporting absenteeism in Sweden in 2008-09 [6], 28% experiencing associated anxiety and depression (UK, data collected 2007-2013) [7], and an estimated 19 missed work days per CRS patient per year (England, recruitment 2013-2015) [8]. In 2011, CRS cost the US healthcare system $8.6 billion with significant direct and indirect costs [9] [10]. Our recent systematic review of literature regarding the cost-effectiveness of surgical intervention confirms the lack of UK perspective economic evaluations, particularly relating to the UK healthcare system [11].

This study forms part of the MACRO Programme, “Defining best Management for Adults with Chronic RhinOsinusitis”, and information from this cost analysis will supplement the analysis of the MACRO randomised controlled trial (RCT), which began recruitment in 2019 [12] [13]. The overarching aims of MACRO are to address major deficiencies in the evidence base for CRS management, establish best practice for management of adults with CRS, and design the ideal patient pathway across primary and secondary care. This observational cohort analysis of CRS surgery patients established the costs to the National Health Service (NHS) of treatments received by these patients from general practices/general practitioners (GPs) and in NHS hospitals in England as inpatients (including day cases) and outpatients, and estimated how much they cost, by polyp-defined subgroup as described below, using linked patient-level primary and secondary care electronic health record data and mortality data from the ONS. The total aggregated costs to the NHS provide a further impetus for trials to evaluate the benefit of surgical intervention.

2. Methods

2.1. *Study design and population*

Linked electronic health records (EHR) from the Clinical Practice Research Datalink (CPRD, primary care, covers ~8% England population) [14], Hospital Episode Statistics (HES, covering inpatient and outpatient care provided in NHS hospitals in England) and Office for National Statistics (ONS, mortality data) databases were used. Scientific and ethical approval for the use of and data linkages within the CPRD primary care data was obtained following application to the Independent Scientific Advisory Committee (ISAC), a non-statutory expert advisory body (Protocol number 16_200). Data and phenotyping algorithms were accessed as part of the CALIBER resource [15] [16].

The population used was a subset of the cohort used in previous work by this group that considered the risk of mortality and cardiovascular events following macrolide prescription in CRS patients [17]. An EHR phenotyping algorithm, comprising primary care and secondary care diagnoses and secondary care procedures deemed to indicate a ‘definite’ diagnosis of CRS, was developed in collaboration with clinicians (see Supplementary Materials, Section A) using a similar approach to that published by Rudmik, Lui and Macdonald [18] [19] [20]. Patients with one or more of these diagnoses or procedures recorded during

For peer review only - http://bmjopen.bmj.com/site/about/guidelines.xhtml

follow-up were classified as 'definite' CRS cases, with the date of diagnosis taken as the date of the first such specified diagnosis or procedure. A further list of 'definite' and 'very likely' surgery OPCS Classification of Interventions and Procedures version 4 (OPCS-4) codes was similarly developed, and the surgical cohort used in this cost analysis was the group of 'definite' CRS patients who had had surgery defined as either 'definitely' or 'very likely' to have been for CRS (see Supplementary Materials, Section A).

Eligible patients entered the analysis cohort on the latest of: current general practice registration date of the patient, date on which research quality data was first provided by the general practice (based on internal CPRD algorithm [14]), their 16th birthday, or study start date (1 April 1997). Cases were required to have a minimum of one year's research-quality information prior to their CRS diagnosis, and a minimum of one day's research-quality data at an individual level following diagnosis. Patients left the cohort on the earliest of: transfer-out date from the general practice, last data collection from general practice, 80th birthday, death (recorded in either CPRD or ONS), or study end date (29 February 2016). Outpatient data were available from 1 April 2003.

A patient's follow-up period began on their CRS diagnosis date and ended when they left the cohort. The index date around which patients' treatment information was centred was the date on which the first CRS-specific surgery took place during the analysis period, meaning that day zero could correspond to any calendar date between 1 April 1997 and 29 February 2016 for any patient. Costs were calculated per patient-quarter, with the surgery date (day zero, index date) placed at the midpoint of quarter zero (Q0), so Q0 contained costs incurred during the 45.7 days before and after surgery as well as on the surgery date itself.

CRS has traditionally been divided into two main phenotypes, CRS with nasal polyps (CRSwNP) and CRS without nasal polyps (CRSsNP), with differences in underlying pathophysiology and association with other conditions such as asthma [21]. CRSwNP patients are more likely to have higher disease burden and more likely to receive surgery [22]. Accordingly, participants were split into two subgroups as in our previous work [13] [17] [23], according to the patient's polyp status: positive polyp status, where polyps were specifically recorded or implied in the EHR at some point during the patient's follow-up (see Supplementary Materials, Section A); or unknown polyp status, meaning either that polyps were absent or that they were perhaps present but were not recorded.

A flowchart illustrating the relationships between the overall diagnosis cohort, the smaller surgical cohort used in this analysis and the two polyp-based subgroups is given in Supplementary Materials, Section A.

2.2. Resource use and unit costs

Costs were calculated from an NHS perspective [24], and prices were in 2017-18 UK pounds sterling. Resource use data were extracted on numbers and types of consultations, investigations, procedures including surgeries, and prescriptions, and classified according to categories available in the relevant published unit costs.

Cost information was categorised for analysis according to these five groups: (i) hospital admitted patient care (APC) from HES APC events (costed as Day Case or Elective Inpatient); (ii) hospital outpatient (OP) attendances from HES OP events; and (iii) primary care visits (GP contacts, practice nurse contacts, other primary care contacts), (iv) primary care antibiotics prescriptions, and (v) other relevant primary care prescriptions, with the latter three groups all from CPRD events data.

Inpatient and outpatient care codes included sinus procedures, nose procedures, nasal polypectomy, and diagnostic imaging, and were grouped into cost categories as detailed in Supplementary Materials, Table B1, and NHS Reference Costs [25] were applied. Inpatient care lasting less than 1 day according to the duration

For peer review only - http://bmjopen.bmj.com/site/about/guidelines.xhtml

captured in CALIBER was costed as a Day Case, and stays longer than 1 day were costed as Elective Inpatient admissions. NHS Reference Costs from 2017-18 were used where available for that category, or earlier NHS Reference Costs were used where required, with uplift to 2017-18 prices using HCHS inflation indices [26]. This was required for outpatient complex sinus procedures (2016-17 prices were used and uplifted), and outpatient major sinus procedures (2015-16 prices used and uplifted).

Unit costs and related information for primary care consultations were obtained from the Personal Social Services Research Unit (PSSRU) [26, 27] (see Supplementary Materials, Table B2). Longitudinal CPRD data which looked at GP contacts in England in 2010-2011 for respiratory tract infections suggested that 1% of adults received treatment for rhinosinusitis from their GP each year, with a median of 4 GP visits, and with 91% of these patients receiving an antibiotic prescription [28], so antibiotic prescriptions from primary care were analysed as a separate category. The dataset contained six commonly used antibiotics that were costed separately, and 38 less common antibiotics that were grouped together and a mean cost applied. The non-antibiotic medications comprised corticosteroids (including combinations with antihistamine) and all other drugs (i.e. painkillers, antihistamines, decongestants, and combinations thereof). Unit costs were obtained from the British National Formulary [29] (see Supplementary Materials, Table B3).

2.3. *Statistical analysis*

Poisson regression was used to calculate incidence rates per quarter (91.3 days) for each of the five types of event listed above in section 2.2, split by polyp status, and unit costs described above were applied to event rates to calculate costs.

Events were censored at 10 years before or after the surgery date for inpatient and primary care, and at 2 years before and after for outpatient care, as including events at dates further away led to small event numbers and therefore large uncertainties (see Supplementary Materials, Table C1, for the denominators at each timepoint, i.e. numbers of patients at risk of having a healthcare event at that moment according to their presence within the follow-up period). The total costs were therefore calculated in the period covering 2 years before and after surgery, split into quarters and also summarised as one-year costs from surgery to allow comparison with other studies.

Discounting was not included as future costs were not projected. Information from electronic records was considered complete, so no imputation was performed. Stata v16 was used to run the analyses [30]. Mean per-person-quarter costs split according to the five categories above were calculated for the quarter containing the surgery date at its mid-point (Q0), and the mean per quarter for the 8 quarters before and 8 quarters after Q0, to provide estimates of costs for surgical patients both in the lead up to their surgery and in subsequent months, as well as around the surgery date itself. Total one-year surgery costs were also calculated per person by summing the 4 quarters from surgery, i.e. summing costs from Q0, Q1, Q2, and Q3.

2.4. *Patient and Public Involvement statement*

Patient and public involvement collaborators are involved in the MACRO programme including its design, conduct, reporting and dissemination, but were not directly involved in the production of this cost analysis publication.

For peer review only

For peer review only - http://bmjopen.bmj.com/site/about/guidelines.xhtml

3. Results

3.1. Patient cohort and demographics

Of the 62,685 patients identified as definitely having CRS in 1997-2016 and registered in the GP practices covered by the CPRD in England, 13,462 received CRS-related surgery and were included in this analysis. Two-thirds (9,056, 67%) were in the polyp-positive subgroup, with the rest (4,406, 33%) in the polyp-unknown subgroup. In the wider group including CRS-definite patients both with and without surgery (n=62,685), these proportions were reversed, namely one-third (23,036, 37%) were polyp-positive and two-thirds (39,649, 63%) were not. These proportions agree with other published work regarding the incidence of nasal polyps in CRS patients [22] [31] [32] [33]. Patient demographic information is in Table 1.

Table 1. Patient demographic information at surgery date.

	Unknown polyp status		Positive polyp status		All patients	
**Total patients, n**	4,406		9,056		13,462	
**Age in years, mean (SD)**	42.4 (14.6)		47.9 (14.7)		46.1 (14.9)	
	n	%	n	%	n	%
**Sex**						
Male	2,029	46.1	6,073	67.1	8,102	60.2
Female	2,377	53.9	2,983	32.9	5,360	39.8
**Ethnicity**						
White	4,038	91.6	8,264	91.3	12,302	91.4
India/South Asia	88	2.0	209	2.3	297	2.2
Black	45	1.0	68	0.8	113	0.8
China/East Asia	42	1.0	81	0.9	123	0.9
Mixed	51	1.2	120	1.3	171	1.3
Unknown	142	3.2	314	3.5	456	3.4
**Region of England**						
North East	51	1.2	179	2.0	230	1.7
North West	809	18.4	1,585	17.5	2,394	17.8
Yorkshire	208	4.7	444	4.9	652	4.8
East Midlands	126	2.9	287	3.2	413	3.1
West Midlands	399	9.1	1,044	11.5	1,443	10.7
East	516	11.7	1,109	12.2	1,625	12.1
South West	627	14.2	1,192	13.2	1,819	13.5
South Central	523	11.9	953	10.5	1,476	11.0
London	543	12.3	1,072	11.8	1,615	12.0
South East	604	13.7	1,191	13.2	1,795	13.3

For peer review only

3.2. Total costs

The total per-person costs to the NHS for one year (Q0-Q3) in patients receiving surgery for CRS was £1,408 in those with unknown polyp status, £2,547 in those with known positive polyp status, and £2,173 overall for all patients. The majority of this expenditure took place in Q0 (Table 2) and the highest single cost category was polypectomy in the polyps-positive group (Table 3). Table 2 shows the mean per-patient-quarter costs, total and by cost component, over the two-year period before the surgery date, during Q0 when surgery took place, and over the two-year period after surgery. Inpatient care costs peaked during Q0 and comprised the majority of Q0 costs. Outpatient costs during Q0 were approximately twice those in the before or after periods but small in comparison to Q0 inpatient costs. The cost of primary care consultations appeared to be lower during Q0 compared to the time preceding surgery and did not rebound in the following two years, and the two categories of primary care prescription costs were low at all times, with little apparent change

For peer review only - http://bmjopen.bmj.com/site/about/guidelines.xhtml

around the surgery date. The standard errors for the mean per-patient-quarter costs in the 2 years before and after surgery are given in Table 4 but are omitted from Table 2 for readability purposes.

Table 2. Costs per patient-quarter, broken down by healthcare/prescription category, by time period, and by polyp status. Prices in 2017-2018 £; DC = Day Case; EL = Elective Inpatient; Abx = antibiotics.

	Inpatient care (DC and EL)	Outpatient	Primary care consultations	Primary care Abx	Primary care Non-abx	TOTAL
**Mean per-patient-quarter costs over 2 years before surgery (-Q1 to -Q8)**						
Unknown polyps (£)	3.35	40.83	16.08	1.70	5.87	**67.82**
Positive polyps (£)	1.53	29.69	16.68	1.15	7.64	**56.69**
All patients (£)	2.13	33.49	16.49	1.33	7.06	**60.50**
**Per person-quarter (in Q0, containing index surgery)**						
Unknown polyps (£)	1117.37	75.68	7.04	1.27	5.54	**1206.90**
Positive polyps (£)	2284.63	62.41	5.59	0.99	7.79	**2361.42**
All patients (£)	1902.00	66.75	6.06	1.08	7.06	**1982.95**
**Mean per-patient per-quarter costs over 2 years after surgery (Q1 to Q8)**						
Unknown polyps (£)	8.64	37.71	6.43	1.26	5.50	**59.54**
Positive polyps (£)	20.70	25.60	4.73	0.95	7.63	**59.62**
All patients (£)	16.87	29.46	5.27	1.05	6.96	**59.61**

3.3. Admitted patient care – Day Case (<1 day) and Elective Inpatient (>1 day)

Hospital admissions costs were £2.13 (SE £1.18) per patient-quarter in the 8 quarters leading up to the surgery quarter (£1.53 (SE £0.93) in polyps-positive patients and £3.35 (SE £2.11) in polyps-unknown patients) (see Table 4). The majority of these costs were during Q0 (£1,902 overall; £1,117 in polyps-unknown patients and £2,285 in polyps-positive patients), and costs per patient-quarter in the subsequent 8 quarters were lower than this peak, at £16.87 (SE £2.97) per patient-quarter (see Table 4).

Regarding revision surgeries, 0.4% of patients in this analysis had a second surgery during the second half of Q0 after their index surgery, and 4.9% of patients received a second surgery at some point during the 8 quarters following Q0. These subsequent surgeries were identified using the same codes as those by which the patients were selected into the cohort, and were included in the costs simply as downstream hospital costs. There was no evidence of a preferred length of wait between first and second surgeries.

Table 3 shows the cost breakdown during Q0. The highest expenditure in polyp-positive patients was on Polypectomy (E081), covering around one-third of all events in this group, and a further 40% corresponded to one of functional endoscopic sinus surgery (FESS), intranasal antrostomy, or intranasal ethmoidectomy, which together formed the major part of the Intermediate/Major/Complex sinus procedures group. In polyp-unknown patients, the highest expenditure was on FESS, intranasal antrostomy, or intranasal ethmoidectomy, which again formed the major part of the Intermediate/Major/Complex sinus procedures group. Types of procedures were grouped together as seen in Table 3 as some codes had small event numbers, thus regressions did not converge unless some groupings were made beyond the categories listed in Supplementary Materials Table B1. Groupings were made based on consecutive unit costs in Elective Inpatient data and the same groupings were used in Day Case data for consistency of reporting. Tables showing costs split by category and polyp subgroup are given in the Supplementary Materials, Section D.

For peer review only - http://bmjopen.bmj.com/site/about/guidelines.xhtml

Table 3. Mean inpatient costs per patient-quarter in Q0 by procedure category, split by polyp status. CT = computed tomography; DC = Day Case; EL = Elective Inpatient; Q0 = quarter containing surgery date at centre. Prices in 2017-2018 £.

Q0	CT/other imaging, DC	Minor nose incl. biopsy, DC	Int nose and Minor sinus, DC	Int/Major/ Complex sinus, DC	CT/other imaging, EL	Minor nose incl. biopsy, EL	Int nose and Minor sinus, EL	Int/Major/ Complex sinus, EL	Polyp-ectomy	TOTAL (Q0)
**Unknown polyps**	0.16	8.62	43.86	243.32	0.86	29.10	110.97	680.49	0.00	**1117.37**
**Positive polyps**	0.02	7.85	40.09	152.60	0.14	28.44	166.57	474.22	1414.69	**2284.63**
**All patients**	0.07	8.10	41.32	181.87	0.37	28.66	148.42	540.56	952.62	**1902.00**

For peer review only

Table 4. Costs during the surgery quarter (Q0) and 2 years before and after. Prices in 2017-2018 £. SE = standard error, DC = Day Case, EL= Elective Inpatient, Q0 = quarter containing surgery date at centre.

	Per-patient costs over 2 years preceding surgery	Mean (SE) per person-quarter over 2 years preceding surgery	Per-patient costs in the quarter containing surgery (Q0)	Mean (SE) per person-quarter over 2 years following surgery	Per-patient costs over 2 years following surgery
**Inpatient costs (DC and EL)**					
Unknown polyps	26.81	3.35 (2.11)	**1117.37**	8.64 (2.97)	69.15
Positive polyps	12.26	1.53 (0.93)	**2284.63**	20.70 (4.56)	165.61
All patients	17.02	2.13 (1.18)	**1902.00**	16.87 (2.97)	134.96
**Outpatient costs**					
Unknown polyps	326.61	40.83 (12.22)	**75.68**	37.71 (8.40)	301.69
Positive polyps	237.49	29.69 (11.41)	**62.41**	25.60 (4.64)	204.77
All patients	267.93	33.49 (11.57)	**66.75**	29.46 (5.78)	235.67
**Primary care consultations**					
Unknown polyps	128.64	16.08 (5.09)	**7.04**	6.43 (0.62)	51.47
Positive polyps	133.48	16.68 (7.02)	**5.59**	4.73 (0.16)	37.87
All patients	131.91	16.49 (6.28)	**6.06**	5.27 (0.28)	42.18
**Primary Care Antibiotics Prescriptions**					
Unknown polyps	13.57	1.70 (0.35)	**1.27**	1.26 (0.04)	10.05
Positive polyps	9.20	1.15 (0.20)	**0.99**	0.95 (0.03)	7.60
All patients	10.63	1.33 (0.24)	**1.08**	1.05 (0.02)	8.38
**Primary Care Non-Antibiotics Prescriptions**					
Unknown polyps	46.93	5.87 (0.80)	**5.54**	5.50 (0.09)	43.96
Positive polyps	61.12	7.64 (1.25)	**7.79**	7.63 (0.07)	61.08
All patients	56.48	7.06 (1.10)	**7.06**	6.96 (0.05)	55.65

3.4. Outpatient attendances

Outpatient care costs were £33.49 (SE £11.57) per patient-quarter in the 8 quarters preceding surgery; (£29.69 (SE £11.41) in polyps-positive patients and £40.83 (SE £12.22) in polyps-unknown patients) (see Table 4), then £66.75 during Q0 (£62.41 for polyps-positive and £75.68 for polyps-unknown). Costs per patient-quarter were reduced from this peak in the subsequent 8 quarters, at around £30 per patient-quarter (see Table 4).

For peer review only - http://bmjopen.bmj.com/site/about/guidelines.xhtml

Table 5 shows the breakdown of costs during Q0 and the quarters immediately preceding and succeeding Q0. The highest expenditure in both subgroups was on CT (computed tomography)/other scans, which comprised around two-thirds CT scans and one-third X-rays. All categories showed a peak in costs in Q0 except for CT/other scans, which instead had a slightly higher peak in the quarter immediately preceding surgery (see Table 5). This tallies with the advice in EPOS 2020 stating that CT scans should always be given before surgery [1]. Tables showing the values split by category and by polyp subgroup, and graphs illustrating this information (i.e. expanding on information in Table 5) are given in Supplementary Materials, Section E.

Table 5. Mean outpatient costs per person-quarter in Q0 and the immediately preceding and succeeding quarters, by procedure category, split by polyp status. CT = computed tomography; Q0 = quarter containing surgery date at centre. Prices in 2017-2018 £.

	CT/other imaging	Minor nose incl. biopsy	Int nose and Minor sinus	Int sinus	Major/ complex sinus	Polyp-ectomy	TOTAL (by person-quarter)
**Polyps unknown**							
**-Q1**	32.30	4.55	11.26	14.70	5.18	-	**67.99**
**Q0**	**29.11**	**4.37**	**13.95**	**17.65**	**10.59**	**-**	**75.68**
**Q1**	25.51	3.16	12.17	12.35	5.51	-	**58.70**
**Polyps positive**							
**-Q1**	25.04	1.84	11.36	12.02	6.17	0.44	**56.87**
**Q0**	**23.03**	**2.93**	**12.47**	**14.46**	**8.66**	**0.85**	**62.41**
**Q1**	14.88	1.63	7.57	8.64	2.97	0.32	**36.00**
**All patients**							
**-Q1**	27.49	2.76	11.33	12.93	5.83	0.29	**60.62**
**Q0**	**25.01**	**3.40**	**12.96**	**15.51**	**9.29**	**0.57**	**66.75**
**Q1**	18.32	2.13	9.06	9.84	3.79	0.21	**43.35**

3.5. *Primary care consultations*

Primary care consultation costs were £16.49 (SE £6.28) per patient-quarter in the 8 quarters preceding surgery (£16.68 (SE £7.02) in polyps-positive patients and £16.08 (SE £5.09) in polyps-unknown patients) (see Table 4), then £6.06 during Q0 (£7.04 in polyps-unknown patients, £5.59 in polyps-positive patients), and costs per patient-quarter were similarly reduced in the subsequent 8 quarters, at around £5-6 per patient-quarter (see Table 4). The highest expenditure in both subgroups was GP face-to-face consultations at the GP practice. Tables showing the values split by category and by polyp subgroup, and graphs illustrating this information, are given in Supplementary Materials, Section F.

3.6. *Primary care prescriptions – antibiotics*

Primary care antibiotics prescription costs were £1.33 (SE £0.24) per patient-quarter in the 8 quarters before surgery (£1.15 (SE £0.20) in polyps-positive patients and £1.70 (SE £0.35) in polyps-unknown patients), then £1.08 during Q0 (£1.27 in polyps-unknown patients, £0.99 in polyps-positive patients), and similar in the subsequent 8 quarters, at around £1 per patient-quarter (see Table 4). The highest expenditure was on tetracyclines, followed by macrolides, and tables showing the values split by category and by polyp subgroup, and graphs illustrating this information, are shown in the Supplementary Materials, Section G.

3.7. *Primary care prescriptions – steroids and other non-antibiotics*

Primary care non-antibiotics prescription costs were primarily for corticosteroids, plus general sinusitis drugs like painkillers and decongestants, and were £7.06 (SE £1.10) per patient-quarter in the 8 quarters before

For peer review only - http://bmjopen.bmj.com/site/about/guidelines.xhtml

surgery (£7.64 (SE £1.25) in polyps-positive patients and £5.87 (SE £0.80) in polyps-unknown patients), then £7.06 during Q0 (£7.79 for polyps-unknown, £5.54 for polyps-positive), and similar in the subsequent 8 quarters, at around £7 per patient-quarter (see Table 4). Tables showing the values split by category and polyp subgroup, and graphs illustrating this information, are given in Supplementary Materials, Section H. This information includes only prescriptions made by the GP, and does not include other medications bought over the counter by the patient.

4. Discussion

In this paper, we have shown that inpatient surgical sinus procedures and nasal polypectomies are the largest healthcare cost in patients receiving surgery for CRS when considering the costs of primary and secondary care to the NHS in England, at around £1000-2000 per person-quarter in the quarter containing the surgery date (Q0). Other secondary and primary healthcare costs in the 8 quarters before and after Q0 are considerably smaller, at around £60 per person-quarter across polyp subgroups. These are average values over the whole population and are not split according to demographic groups.

Average total costs across secondary and primary care settings were £1,983 per patient overall during Q0, or £2,361 per polyp-positive patient and £1,207 per polyp-unknown patient, in 2017-2018 prices. Hospital overnight admission and day case inpatient costs incurred during Q0 were the costliest category across the 4.25-year analysis period, dwarfing other cost components. Primary care prescription costs were low across both groups, with antibiotics costing around £1 per person-quarter and non-antibiotics around £7 per person-quarter. Outpatient care costs appeared higher than primary care costs at around £30 per person-quarter before and after surgery, and around £67 per person-quarter during Q0. Primary care consultation costs appeared higher before surgery than after (£16 vs. £6 per person-quarter), and inpatient care costs appeared higher after surgery than before (£17 vs. £2 per person-quarter). These findings suggest that the costs to the NHS associated with CRS, especially the non-surgical costs, are currently low. They also suggest that CRS surgery does not appreciably impact overall management costs, either upwards or downwards, although these costs are low so it would be difficult to see a meaningful change. These values are presented as descriptive statistics and formal significance testing among the various categories and timepoints described above has not been performed.

There were certain limitations in this analysis. Only costs for those patients for whom CRS surgery codes were recorded during the time period were included, and the analysis was based around the date of their first CRS surgery as captured during the analysis time period. If a patient had another surgery before they entered the cohort, this would not have appeared in the dataset, thus we cannot be entirely certain that the index surgery was indeed the patient's first CRS surgery.

Other limitations relate to other aspects of coding and identification of patients and their treatments, as the dataset used was collected by hospitals and GP practices for reimbursement and clinical management purposes, and not specifically for research purposes, and patients were not prospectively recruited into the dataset so there was no prospectively defined baseline. For example, identification of CRS patients and their diagnosis dates and treatment information was performed using phenotyping code lists of treatments and diagnostic markers, using methodology common to observational analyses using routine data and expert clinical opinion to determine the code lists. Thus the identification of patients and treatments was reliant on patients' practitioners or coding staff having entered certain codes or combinations of codes. Furthermore, the coding regarding polyp status is limited, as there is no code to confirm that a patient does not have

For peer review only - http://bmjopen.bmj.com/site/about/guidelines.xhtml

polyps, there is only the absence of a positive report of polyps. This is based on treatments recorded, including the reporting of a polypectomy, leading to a certain circularity when reporting the treatments received by subgroup.

This analysis used CPRD for primary care information, which covers around 8% of the population of England, and is broadly representative of the UK although with acknowledged gaps including people who are universally underrepresented in UK healthcare systems, for example homeless people and those with non-standard residency or migration status [34].

We used the standard English NHS cost perspective, although we did not have information on Personal Social Services, the costs of which would normally be included in analyses for the National Institute for Health and Care Excellence (NICE) [24], or on other community-based health care such as Improving Access to Psychological Therapies (IAPT), which might be relevant to this population. We also did not have emergency care costs, but we do not anticipate that this would be a major part of this care pathway. We had no information on wider societal costs, for example relating to productivity (time off work) or any out-of-pocket costs for patients. It is possible therefore that information regarding factors that are important to patients and their families was not captured in this analysis.

Other work published in this area has focused mostly on US costs and used different unit costs and included different cost categories. Bhattacharyya et al. [35] investigated costs of CRSwNP patients in a US claims database using information gathered in 2013-2014, beginning at CRS diagnosis. When CRSwNP patients undergoing FESS were compared to CRSwNP patients not undergoing surgery, they found that the extra cost of surgery during that first year was $13,532. This was an observational, retrospective case-control study, meaning that treatment decisions were not randomly assigned within the CRSwNP group, and therefore any differences in costs according to treatment decisions were susceptible to selection bias. Studies have also been published examining cost breakdowns of CRS patients in the US regarding the distribution of expenditure across different care categories. For example, Caulley et al. [36] considered all CRS patients in the US Medical Expenditure Panel Survey, taking a cross-section in 2011, and found that ambulatory office-based consultations and prescriptions each accounted for a greater proportion of expenditure than inpatient hospital visits, although this was for all CRS patients, not just those receiving surgery, and the US system is both structured and financed quite differently from the UK system. For example, certain medications available in North America for the management of CRSwNP like monoclonal antibodies are not available in the English NHS, and therefore no patient in the present analysis had received these. Aspirin desensitisation also has very restricted availability in the UK and is only offered in a small number of UK centres so was also not captured here. Bhattacharyya et al. [35] however reported that prescription costs were not a major part of CRS costs for CRS patients undergoing surgery or not undergoing surgery in their observational study using the Truven Health MarketScan US claims database.

Our analysis only included surgical CRS patients, and did not attempt to include non-surgical patients to allow comparison of treatments received by surgical and non-surgical patients, as this is difficult to do in observational datasets and can lead to misleading results, with important limitations due to the lack of randomisation, as there are unobserved and unmeasured confounders that can govern what treatment people receive. RCTs aim to identify and capture these confounders, using a large enough sample size that there is balance across the arms, and the analysis is adjusted for confounders. There are methods such as instrumental variable analysis that attempt to mimic randomisation using statistical methods, but it is typically hard to find a suitable instrument [37] [38]. Using random allocation to assign treatments is therefore a powerful tool in eliminating selection bias, and is not available in analysis using routine observational data, hence the importance of the MACRO RCT [12], which began recruiting patients in 2019.

For peer review only - http://bmjopen.bmj.com/site/about/guidelines.xhtml

MACRO is randomising patients 1:1:1 to receive appropriate medical therapy (AMT), surgery plus AMT, or long-term low-dose macrolides plus AMT, and collecting all relevant information required to make a randomised comparison between surgery and non-surgical treatments in a full cost-utility analysis [39] [40] [41]. The MACRO RCT will provide key information regarding changes in quality of life on receiving surgery for CRS and allow us to provide information regarding the relative cost-effectiveness of surgery and other treatments in the UK context.

5. Conclusion

This is the first study we are aware of that analysed the costs of primary and secondary healthcare received by patients undergoing surgery for chronic rhinosinusitis using English NHS costs. It included a large sample size that was representative of care given by the NHS in England and showed that the inpatient costs including CRS surgery itself were around £2000 during the quarter containing surgery, and that the cost of management before and after surgery in primary and secondary care settings was low in comparison at around £60 per person-quarter in the two preceding and subsequent years.

This study reports important new evidence regarding the cost of English NHS healthcare costs for patients receiving CRS surgery, and provides further justification for the use of randomised clinical trials to investigate the relative cost-effectiveness of surgical treatments for CRS, as well as providing useful information that can be applied in future work in the UK and similar contexts, including our own future analysis of the MACRO trial data.

Acknowledgements

The authors wish to acknowledge the valuable input from the MACRO WS1 study team and the wider MACRO programme team, including Hannah Evans, and our Patient and Public Involvement Collaborators.

Contributorship statement

CSC was lead author, and wrote the first draft of the article. CSC and EW cleaned and analysed the data regarding rates of events. CSC and SM planned and conducted the cost analyses. EW, CSC, SM, MT, CH and CP formulated the phenotyping codelists for identifying the patient cohort and relevant treatments and diagnoses. CSC, EW, SD, JRC, MT, HB, AGMS, CP, CH and SM were involved in formulating the overall research question, and designing and conducting the study. All authors contributed to and approved the final manuscript.

Competing interests:

In addition to the NIHR funding for this work acknowledged above, the following interests outside the submitted work have been declared by the authors. EW: personal fees from AstraZeneca for provision of training on propensity score methodology unrelated to the current manuscript; JC: grants from UK Medical Research Council (grant numbers MC_UU_12023/21 and MC_UU_12023/29), consultancy from AstraZeneca and Novartis on statistical methodology for the analysis of partially observed data, and book royalties from “Multiple Imputation and its Application” (Wiley) and “Meta-analysis using R” (Springer); CP: personal fees for advisory work from GSK and Sanofi, and work as a Trustee of Fifth Sense; CH: Advisory board for GSK, Sanofi and AstraZeneca and speakers fees for Mylan and Intersect. The remaining authors have nothing to declare.

For peer review only - http://bmjopen.bmj.com/site/about/guidelines.xhtml

For peer review only

Funding

This work was supported by the National Institute for Health Research (NIHR) under its Programme Grants for Applied Research (PGfAR) Programme (Grant Reference Number RP-PG-0614-20011). The views expressed are those of the author(s) and not necessarily those of the National Health Service, the National Institute for Health Research, or the Department of Health and Social Care. SD is supported by: (i) National Institute for Health Research (NIHR) University College London Hospitals Biomedical Research Centre (UCLH BRC); (ii) Health Data Research UK (award ref: LOND1), which is funded by the UK Medical Research Council, Engineering and Physical Sciences Research Council, Economic and Social Research Council, Department of Health and Social Care (England), Chief Scientist Office of the Scottish Government Health and Social Care Directorates, Health and Social Care Research and Development Division (Welsh Government), Public Health Agency (Northern Ireland), British Heart Foundation and Wellcome Trust; (iii) BigData@Heart programme that has received funding from the Innovative Medicines Initiative 2 Joint Undertaking under grant agreement no. 116074. This Joint Undertaking receives support from the European Union's Horizon 2020 research and innovation programme and EFPIA; (iv) British Heart Foundation Accelerator Award (AA/18/6/24223).

Data sharing statement

The data were pseudonymised patient-level data from the CALIBER resource and are not publicly available. Analysis code can be made available on reasonable request and in accordance with relevant guidance.

Ethics statement

Not applicable as human participants were not directly included in this work. This study used routinely collected data. Scientific and ethical approval for the use of and data linkages within the Clinical Practice Research Datalink (CPRD) primary care data was obtained following application to the Independent Scientific Advisory Committee (ISAC), a non-statutory expert advisory body (Protocol number 16_200).

6. References

[1] W. J. Fokkens, V. J. Lund, C. Hopkins, P. W. Hellings, R. Kern, S. Reitsma, M. Bernal-Sprekelsen and J. Mullol, “European Position Paper on Rhinosinusitis and Nasal Polyps 2020,” *Rhinology,* vol. 58, no. Suppl. 29, pp. 1-464, 2020.

[2] D. Hastan, W. J. Fokkens, C. Bachert, R. B. Newson, J. Bislimovska, A. Bockelbrink, P. J. Bousquet, G. Brozek, A. Bruno, S. E. Dahlen, B. Forsberg, M. Gunnbjörnsdóttir, L. Kasper, U. Krämer, M. L. Kowalski, B. Lange, B. Lundbäck, E. Salagean, A. Todo-Bom, P. Tomassen, E. Toskala, C. M. van Drunen, J. Bousquet, T. Zuberbier, D. Jarvis and P. Burney, “Chronic rhinosinusitis in Europe - an underestimated disease. A GA²LEN study.,” *Allergy,* vol. 66, no. 9, pp. 1216-1223, 2011.

[3] B. Lange, R. Holst, T. Thilsing, J. Baelum and A. Kjeldsen, “Quality of life and associated factors in persons with chronic rhinosinusitis in the general population: A prospective questionnaire and clinical cross-sectional study,” *Clinical Otolaryngology,* vol. 38, no. 6, pp. 474-480, 2013.

[4] R. E. Gliklich and R. Metson, “The health impact of chronic sinusitis in patients seeking otolaryngologic care,” *Otolaryngology - Head and Neck Surgery,* vol. 113, no. 1, pp. 104-109, 1995.

For peer review only - http://bmjopen.bmj.com/site/about/guidelines.xhtml

[5] N. Bhattacharyya, “Contemporary Assessment of the Disease Burden of Sinusitis,” *Allergy & Rhinology,* vol. 1, no. 1, 2010.

[6] P. Sahlstrand-Johnson, B. Ohlsson, C. von Buchwald, M. Jannert and M. Ahlner-Elmqvist, “A multi-centre study on quality of life and absenteeism in patients with CRS referred for endoscopic surgery,” *Rhinology ,* vol. 49, no. 4, pp. 420-428, 2011.

[7] S. E. Erskine, C. Hopkins, A. Clark, S. Anari, A. Robertson, S. Sunkaraneni, J. A. Wilson, J. Beezhold and C. M. Philpott, “Chronic rhinosinusitis and mood disturbance,” *Rhinology,* vol. 55, no. 2, pp. 113-119, 2017.

[8] N. W. Wahid, R. Smith, A. Clark, M. Salam and C. M. Philpott, “The socioeconomic cost of chronic rhinosinusitis study,” *Rhinology,* vol. 58, no. 2, pp. 112-125, 2020.

[9] V. K. Anand, “Epidemiology and economic impact of rhinosinusitis,” *Annals of otology, rhinology & laryngology, Suppl.,* vol. 193, pp. 3-5, 2004.

[10] N. Bhattacharyya , “The economic burden and symptom manifestations of chronic rhinosinusitis,” *American Journal of Rhinology & Allergy,* vol. 17, no. 1, pp. 27-32, 2003.

[11] A. Bernic, O. Dessouky, C. Philpott, S. Morris and C. Hopkins, “Cost-effective surgical intervention in chronic rhinosinusitis,” *Current Otorhinolaryngology Reports,* vol. 3, pp. 117-123, 2015.

[12] C. Philpott, S. le Conte, D. Beard, J. Cook, W. Sones, S. Morris, C. S. Clarke, M. Thomas, P. Little, J. Vennik, V. Lund, H. Blackshaw, A. Schilder, S. Durham, S. Denaxas, J. Carpenter, J. Boardman and C. Hopkins, “Clarithromycin and endoscopic sinus surgery for adults with chronic rhinosinusitis with and without nasal polyps: study protocol for the MACRO randomised controlled trial,” *Trials ,* vol. 20, p. 246, 2019.

[13] H. Blackshaw, J. Vennik, C. Philpott, M. Thomas, C. Eyles, J. Carpenter, C. S. Clarke, S. Morris, A. Schilder, V. Lund, P. Little, S. Durham, S. Denaxas, E. Williamson, D. Beard, J. Cook, S. le Conte, K. Airey, J. Boardman and C. Hopkins, “Expert panel process to optimise the design of a randomised controlled trial in chronic rhinosinusitis (the MACRO programme),” *Trials,* vol. 20, p. 230, 2019.

[14] E. Herrett, A. M. Gallagher, K. Bhaskaran, H. Forbes, R. Mathur, T. van Staa and L. Smeeth, “Data Resource Profile: Clinical Practice Research Datalink (CPRD),” *International Journal of Epidemiology,* vol. 44, no. 3, pp. 827-836, 2015.

[15] S. Denaxas, A. Gonzalez-Izquierdo, K. Direk, N. K. Fitzpatrick, G. Fatemifar, A. Banerjee, R. J. Dobson, L. J. Howe, V. Kuan, R. T. Lumbers, L. Pasea, R. S. Patel, A. D. Shah, A. D. Hingorani, C. Sudlow and H. Hemingway, “UK phenomics platform for developing and validating electronic health record phenotypes: CALIBER,” *Journal of the American Medical Informatics Association,* vol. 26, no. 12, p. 1545–1559, 2019.

[16] V. Kuan, S. Denaxas, A. Gonzalez-Izquierdo, K. Direk, O. Bhatti, S. Husain, S. Sutaria, M. Hingorani, D. Nitsch, C. A. Parisinos, R. T. Lumbers, R. Mathur, R. Sofat, J. P. Casas, I. C. Wong, H. Hemingway and A. D. Hingorani, “A chronological map of 308 physical and mental health conditions from 4 million individuals in the English National Health Service,” *Lancet Digital Health,* vol. 1, pp. e63-77, 2019.

[17] E. Williamson, S. Denaxas, S. Morris, C. S. Clarke, M. D. Thomas, H. Evans, K. Direk, A. Gonzalez-Izquierdo, P. Little, V. Lund, H. Blackshaw, A. Schilder, C. Philpott, C. Hopkins and J. Carpenter, “Risk of mortality and cardiovascular events following macrolide prescription in chronic rhinosinusitis patients:

For peer review only - http://bmjopen.bmj.com/site/about/guidelines.xhtml

a cohort study using linked primary care electronic health records," *Rhinology,* vol. 57, no. 4, pp. 252-260, 2019.

[18] L. Rudmik, Y. Xu, E. Kukec, M. Liu, S. Dean and H. Quan, "A validated case definition for chronic rhinosinusitis in administrative data: a Canadian perspective," *International Forum of Allergy & Rhinology,* vol. 6, no. 11, pp. 1167-1172, 2016.

[19] J. T. Lui and L. Rudmik, "Case definitions for chronic rhinosinusitis in administrative data: A systematic review," *American Journal of Rhinology & Allergy,* vol. 29, no. 5, pp. e146-151, 2015.

[20] K. I. Macdonald, S. J. Kilty and C. van Walraven, "Development and validation of an administrative data algorithm to identify adults who have endoscopic sinus surgery for chronic rhinosinusitis," *Journal of Otolaryngology - Head & Neck Surgery,* vol. 46, no. 1, p. 38, 2017.

[21] C. Hopkins, C. Philpott, S. Carrie, J. Blythe, M. Thomas, P. Little, G. Scadding, V. J. Lund, S. Wilkes, A. Swift and H. Saleh, "Commissioning guide: Chronic Rhinosinusitis," ENT UK, 2016.

[22] A. Banerji, J. F. Piccirillo, S. E. Thawley, R. G. Levitt, K. B. Schechtman, M. A. Kramper and D. L. Hamilos, "Chronic rhinosinusitis patients with polyps or polypoid mucosa have a greater burden of illness," *American Journal of Rhinology,* vol. 21, pp. 19-26, 2007.

[23] C. Hopkins, E. Williamson, S. Morris, C. S. Clarke, M. Thomas, H. Evans, P. Little, V. J. Lund, H. Blackshaw, A. Schilder, C. Philpott, J. Carpenter and S. Denaxas, "Antibiotic usage in chronic rhinosinusitis: analysis of national primary care electronic health records," *Rhinology,* vol. 57, no. 6, pp. 420-442, 2019.

[24] NICE, "Guide to the methods of technology appraisal," 2013. [Online]. Available: https://www.nice.org.uk/process/pmg9.

[25] NHS Improvement, "NHS Reference Costs," [Online]. Available: https://improvement.nhs.uk/resources/national-cost-collection/.

[26] L. Curtis and A. Burns, "Unit Costs of Health and Social Care 2018," Personal Social Services Research Unit (PSSRU), University of Kent, Canterbury, 2018.

[27] L. Curtis and A. Burns, "Unit Costs of Health and Social Care 2015," Personal Social Services Research Unit (PSSRU), University of Kent, Canterbury, 2015.

[28] M. C. Gulliford, A. Dregan, M. V. Moore, M. Ashworth, T. van Staa, G. McCann, J. Charlton, L. Yardley, P. Little and L. McDermott, "Continued high rates of antibiotic prescribing to adults with respiratory tract infection: survey of 568 UK general practices," *BMJ Open,* vol. 4, no. 10, p. e006245, 2014.

[29] Joint Formulary Committee, "British National Formulary (online)," London.

[30] StataCorp, *Stata Statistical Software: Release 16,* College Station, TX, USA: StataCorp LP, 2019.

[31] C. Hopkins, J. P. Browne, R. Slack, V. Lund, J. Topham, B. Reeves, L. Copley, P. Brown and J. van der Meulen, "The national comparative audit of surgery for nasal polyposis and chronic rhinosinusitis," *Clinical Otolaryngology,* vol. 31, no. 5, pp. 390-398, 2006.

[32] C. Hopkins, P. Andrews and C. E. Holy, "Does time to endoscopic sinus surgery impact outcomes in chronic rhinosinusitis? Retrospective analysis using the UK clinical practice research data," *Rhinology,* vol. 53, pp. 18-24, 2015.

For peer review only - http://bmjopen.bmj.com/site/about/guidelines.xhtml

[33] E. S. Lourijsen, C. A. de Borgie, M. Vleming and W. J. Fokkens, “Endoscopic sinus surgery in adult patients with chronic rhinosinusitis with nasal polyps (PolypESS): study protocol for a randomised controlled trial,” *Trials,* vol. 18, p. 39, 2017.

[34] T. Elwell-Sutton, J. Fok, F. Albanese, H. Mathie and R. Holland, “Factors associated with access to care and healthcare utilization in the homeless population of England,” *Journal of Public Health,* vol. 39, no. 1, pp. 26-33, 2017.

[35] N. Bhattacharyya, S. Villeneuve, V. N. Joish, C. Amand, L. Mannent, N. Amin, P. Rowe, J. Maroni, L. Eckert, T. Yang and A. Khan, “Cost Burden and Resource Utilization in Patients With Chronic Rhinosinusitis and Nasal Polyps,” *Laryngoscope,* vol. 129, pp. 1969-1975, 2019.

[36] L. Caulley, K. Thavorn, L. Rudmik, C. Cameron and S. J. Kilty, “Direct costs of adult chronic rhinosinusitis by using,” *Journal of Allergy and Clinical Immunology,* vol. 136, no. 6, pp. 1517-1522, 2015.

[37] A. Basu, J. J. Heckman, S. Navarro-Lozano and S. Urzua, “Use of instrumental variables in the presence of heterogeneity and self-selection: an application to treatments of breast cancer patients,” *Health Economics,* vol. 16, pp. 1133-1157, 2007.

[38] A. Basu, “Estimating person-centered treatment (PeT) effects using instrumental variables: an application to evaluating prostate cancer treatments,” *Journal of Applied Econometrics,* vol. 29, no. 4, pp. 671-691, 2014.

[39] G. A. Scangas, A. K. Remenschneider, B. M. Su, M. G. Shrime and R. Metson, “Cost Utility Analysis of Endoscopic Sinus Surgery for Chronic Rhinosinusitis With and Without Nasal Polyposis,” *The Laryngoscope,* vol. 127, p. 29–37, 2017.

[40] L. Rudmik, Z. M. Soler, J. C. Mace, R. J. Schlosser and T. L. Smith, “Economic Evaluation of Endoscopic Sinus Surgery versus Continued Medical Therapy for Refractory Chronic Rhinosinusitis,” *Laryngoscope,* vol. 125, no. 1, pp. 25-32, 2015.

[41] G. A. Scangas, B. M. Su, A. K. Remenschneider, M. G. Shrime and R. Metson, “Cost utility analysis of endoscopic sinus surgery for chronic rhinosinusitis,” *International Forum of Allergy & Rhinology,* vol. 6, no. 6, pp. 582-589, 2016.

For peer review only

For peer review only - http://bmjopen.bmj.com/site/about/guidelines.xhtml

Title:

Observational retrospective study calculating health service costs of patients receiving surgery for chronic rhinosinusitis in England, using linked patient-level primary and secondary care electronic data

Authors:

Caroline S Clarke* (caroline.clarke@ucl.ac.uk), Elizabeth Williamson, Spiros Denaxas, James R Carpenter, Mike Thomas, Helen Blackshaw, Anne GM Schilder, Carl Philpott, Claire Hopkins and Stephen Morris, on behalf of the MACRO programme team

For peer review only

Supplementary Materials

A. Section A – Codelists

i. CRS diagnostic terms – definite CRS

The following codelist was developed to identify patients with chronic rhinosinusitis, according to "definite" diagnostic events. Patients with one or more of these diagnoses or procedures recorded during follow-up were considered to be definite CRS cases, with the date of diagnosis taken to be the date of the first such diagnosis or procedure.

Code type	ICD-10 Code		Description ("definite" CRS diagnosis)
ICD-10	J32		Chronic sinusitis
	J320		Chronic maxillary sinusitis
	J321		Chronic frontal sinusitis
	J322		Chronic ethmoidal sinusitis
	J323		Chronic sphenoidal sinusitis
	J324		Chronic pansinusitis
	J328		Other chronic sinusitis
	J329		Chronic sinusitis, unspecified
	J33		Nasal polyp
	J330		Polyp of nasal cavity
	**MEDCODE**	**Read code**	
MEDCODE with corresponding Read Code	811	7406000	Nasal polypectomy
	848	7411200	Intranasal antrostomy
	977	H110.00	Polyp of nasal cavity
	1673	7412100	Intranasal ethmoidectomy
	1674	H132.00	Chronic ethmoidal sinusitis
	2257	H13..00	Chronic sinusitis
	4433	H130.00	Chronic maxillary sinusitis
	4686	H11..00	Nasal polyps
	5437	H13z.00	Chronic sinusitis NOS
	6411	2D33.00	O/E - nasal polyp present

For peer review only - http://bmjopen.bmj.com/site/about/guidelines.xhtml

	6491	7416	FESS/Therapeutic endoscopy of nose and sinus
	7243	7.42E+03	FESS/Post operative division of adhesions
	8220	7416300	FESS/Uncinectomy
	10546	H13..11	Chronic rhinosinusitis
	11744	H11y100	Polyp of ethmoidal sinus
	14749	H110z00	Polyp of nasal cavity NOS
	14888	H11z.00	Nasal polyp NOS
	15163	H131.00	Chronic frontal sinusitis
	16626	7416D00	FESS - post operative removal of polyps (local anaesthetic)
	17173	H135.00	Recurrent sinusitis
	18083	7416C00	FESS - post operative suction clearance (local anaesthetic)
	18869	7416900	FESS/Antrostomy via middle meatus
	19742	H11y.11	Nasal sinus polyps
	20806	7415.11	FESS - diagnostic nasal antroscopy
	20832	7416500	FESS/Anterior ethmoidectomy
	21213	7412700	Radical frontal sinus antrostomy
	21923	7416.11	FESS/Therapeutic nasal antroscopy
	27869	7412800	Frontal sinusotomy NEC
	30990	S832300	Open wound of nasal sinus
	33709	7415z00	FESS/Diagnostic endoscopy of nose or sinus NOS
	33922	7416700	FESS/Anterior and posterior ethmoidectomy
	34165	7412300	Transantral ethmoidectomy
	34973	7416z00	FESS - therapeutic endoscopy of nose or sinus NOS
	35389	7416B00	FESS/Excisional surgery to middle turbinate
	35897	H11y200	Polyp of maxillary sinus
	37481	7416800	FESS/Sphenoidectomy
	39501	H13y000	Chronic pansinusitis
	42166	7416F00	Functional endoscopic sinus surg - polypectomy nasal sinus
	45995	7416y00	FESS - therapeutic endoscopy of nose or sinus OS
	46336	7416A00	FESS/Antrostomy via inferior meatus
	48703	H133.00	Chronic sphenoidal sinusitis
	49348	7416600	FESS/Anterior ethmoidectomy and frontal recess dissection
	49548	H13y.00	Other chronic sinusitis
	54375	H13yz00	Other chronic sinusitis NOS
	59019	7416400	FESS/Uncinectomy and excision of bulla
	59339	7415200	FESS - diagnostic antroscopy via middle meatus
	61281	H11y300	Polyp of sphenoidal sinus
	62936	7416200	FESS/Therapeutic antroscopy via middle meatus
	63733	Hyu2200	[X]Other chronic sinusitis
	64359	7412y00	Other specified operation on frontal sinus
	68003	7M1B000	Functional endoscopic sinus surgery
	69714	7406700	Nasal polypectomy using auto-debrider
	86064	7M1B100	Functional endoscopic nasal surgery
	**OPCS code**		
**OPCS**	E081		Polypectomy of internal nose
	E133		Intranasal antrostomy
	E142		Intranasal ethmoidectomy
	Y761		Functional endoscopic sinus surgery
	Y762		Functional endoscopic nasal surgery

For peer review only - http://bmjopen.bmj.com/site/about/guidelines.xhtml

ii. CRS surgery terms – definite or very likely CRS surgery

The subgroup of surgical patients were those who had also undergone surgery that was "definitely" or "very likely" to have been for CRS and the codelist for "very likely" CRS surgery is given in a second table below.

Code type	Code	Description ("very likely" CRS surgery)
OPCS	E13	Other operations on maxillary antrum
	E131	Drainage of maxillary antrum NEC
	E132	Excision of lesion of maxillary antrum
	E136	Puncture of maxillary antrum
	E138	Other specified other operations on maxillary antrum
	E139	Unspecified other operations on maxillary antrum
	E14	Operations on frontal sinus
	E144	Transantral ethmoidectomy
	E147	Median drainage of frontal sinus
	E148	Other specified operations on frontal sinus
	E149	Unspecified operations on frontal sinus
	E15	Operations on sphenoid sinus
	E151	Drainage of sphenoid sinus
	E152	Puncture of sphenoid sinus
	E158	Other specified operations on sphenoid sinus
	E159	Unspecified operations on sphenoid sinus
	E16	Other operations on frontal sinus
	E161	Frontal sinus osteoplasty
	E162	Drainage of frontal sinus NEC
	E168	Other specified other operations on frontal sinus
	E169	Unspecified other operations on frontal sinus

For peer review only - http://bmjopen.bmj.com/site/about/guidelines.xhtml

iii. Polyp diagnostic terms

The codelist for determining whether a patient was categorised as being in the positive-polyps subgroup or the unknown-polyps subgroup is below. Those patients exhibiting one or more of these codes were classified as "polyps positive" and those lacking these codes were "polyps unknown".

Code type	Code	Description ("positive polyps")
ICD-10	J33	Nasal polyp
	J330	Polyp of nasal cavity
	J331	Polypoid sinus degeneration
	J338	Other polyp of sinus
	J339	Nasal polyp, unspecified
MEDCODE	11744	Polyp of ethmoidal sinus
	14749	Polyp of nasal cavity NOS
	14888	Nasal polyp NOS
	16626	FESS - post operative removal of polyps (local anaesthetic)
	19742	Nasal sinus polyps
	24535	Removal of antrochoanal polyp
	35897	Polyp of maxillary sinus
	42166	Functional endoscopic sinus surg - polypectomy nasal sinus
	4341	Antral (maxillary) polyp
	44518	Other polyp of sinus NOS
	44934	Polypoid sinus degeneration
	4686	Nasal polyps
	50528	Polypoid sinus degeneration NOS
	61281	Polyp of sphenoidal sinus
	6411	O/E - nasal polyp present
	69714	Nasal polypectomy using auto-debrider
	811	Nasal polypectomy
	91482	Polyp of frontal sinus
	977	Polyp of nasal cavity
OPCS	E081	Polypectomy of internal nose

For peer review only

For peer review only - http://bmjopen.bmj.com/site/about/guidelines.xhtml

iv. Flowchart illustrating the application of the codelists in this section of the Supplementary Materials

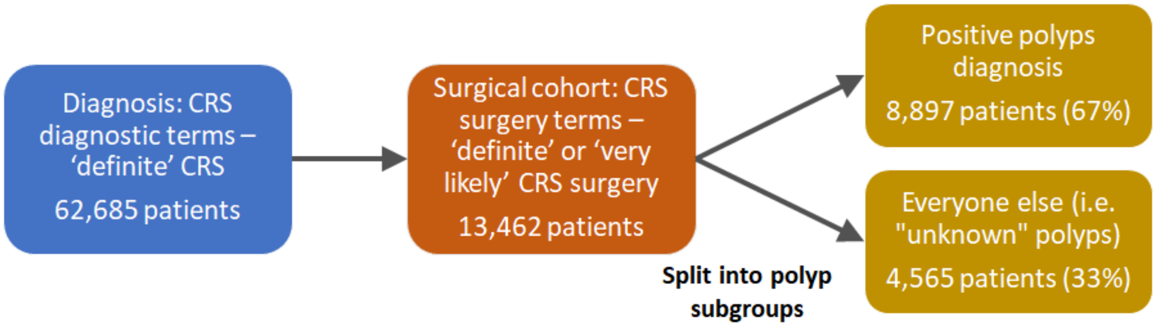

CRS diagnostic terms: Defined as described in Supplementary Materials Section A

CRS surgery terms: Defined as described in Supplementary Materials Section A

Polyp diagnostic terms: Defined as described in Supplementary Materials Section A

For peer review only

B. Section B – Unit costs and other input information

NHS Reference Cost categories (2017-18 prices)	Inpatient (APC) events		Outpatient (OP) events	
	Elective Inpatient unit cost (£)	Day Case unit cost (£)	Outpatient unit cost (£)	OPCS/ICD-10 code(s)
Complex sinus procedure	£3,972.25	£2,383.70	£114.23	E147, E152, E153
Major Sinus Procedures	£2,918.76	£2,298.06	£118.52	E131, E132, E141, E143, E144, E146, E148, E149, E151, E154, E158, E159, E161, E162, E168, E169, E171, E172
Intermediate Sinus Procedures	£2,536.77	£1,920.27	£145.73	E133, E138, E139, E142, E178, E179, Y403, Y761, Y762, Z238, Z239
Minor Sinus Procedures	£2,320.64	£1,654.96	£113.14	E136
Intermediate Nose Procedures	£2,241.99	£1,128.15	£164.98	E058, E059, E088, E089, E108, E109, Y408, Y409, Z228, Z229
Nasal Polypectomy	£2,193.28	£1,587.09	£130.83	E081
Minor Nose Procedures	£1,876.76	£1,325.83	£148.90	E061, E062, E063, E064, E068, E069
Excision or Biopsy, of Lesion of Internal Nose	£1,748.75	£1,054.22	£144.62	E082, E101, E134
Other Specified Diagnostic Imaging of Other Sites, 19 years and over	£214.29	£214.29	£214.29	U064, U068, U069, U217
Computerised Tomography Scan of One Area, without Contrast, 19 years and over	£88.21	£88.21	£88.21	U051, U061, U212

Table B1. Unit costs, inpatient and outpatient health care contacts, from published NHS Reference Costs *[23]*. **Prices in 2017-2018 £.**

For peer review only

Mean duration of visit, minutes	GP	Nurse/Other	References
Standard consultation visit	9.22	9.22	[24]
Specific clinic or home visit	17.20	17.20	[25]
Telephone call	7.10	7.10	[25]
**Unit cost per visit**			
Standard consultation visit	£37.34	£6.45	[24]
Specific clinic or home visit	£69.66	£12.04	[24]
Telephone call	£28.76	£4.97	[24]

Table B2. Unit costs, primary care consultations. Including information on the duration of a contact, and the unit cost calculated on that basis. Prices in 2017-2018 £.

Antibiotics	Unit cost
Cephalosporin	£4.97
Macrolide	£6.64
Metronidazole	£2.13
Penicillin	£1.42
Quinolone	£2.45
Tetracycline	£8.57
Other antibiotic	£4.36
**Non-antibiotics**	**Unit cost**
Corticosteroids	£8.08
All other drugs	£4.69

Table B3. Unit costs of primary care medications, using the British National Formulary (BNF) online *[26]*. Prices in 2017-2018 £.

For peer review only - http://bmjopen.bmj.com/site/about/guidelines.xhtml

For peer review only

C. Section C – Numbers of patients “at risk” of a primary care, outpatient, or inpatient care event, centred on surgery date at midpoint of Q0

Years from surgery	-10	-8	-6	-4	**-2**	**0**	**2**	4	6	8	10
Quarters from surgery	-40	-32	-24	-16	**-8**	**0**	**8**	16	24	32	40
**Numbers "at risk" of primary care consultation/prescription event**											
Positive polyps	1551	2489	3606	4995	**6697**	**9056**	**7028**	5443	4109	2896	1897
Unknown polyps	812	1250	1773	2451	**3289**	**4406**	**3199**	2365	1689	1182	783
All patients	2363	3739	5379	7446	**9986**	**13462**	**10227**	7808	5798	4078	2680
**Numbers "at risk" of inpatient care event**											
Positive polyps	1451	2362	3434	4804	**6460**	**8689**	**6713**	5242	4048	2930	2007
Unknown polyps	768	1198	1717	2360	**3163**	**4205**	**3239**	2408	1744	1247	833
All patients	2219	3560	5151	7164	**9623**	**12894**	**9952**	7650	5792	4177	2840
**Numbers "at risk" of outpatient care event**											
Positive polyps	0	0	0	0	**1135**	**8755**	**2962**	9	0	0	0
Unknown polyps	0	0	0	0	**633**	**4242**	**1234**	5	0	0	0
All patients	0	0	0	0	**1768**	**12997**	**4196**	14	0	0	0

Table C1. Numbers of patients “at risk” of a primary care, outpatient, or inpatient care event, centred on surgery date.

For peer review only

For peer review only - http://bmjopen.bmj.com/site/about/guidelines.xhtml

D. Section D – Mean inpatient costs per person-quarter, centred on surgery date at midpoint of Q0

Polypectomy costs were not split into DC and EL categories according to the length of stay as that regression did not converge, so instead a weighted cost was used according to the proportion of DC and EL polypectomies (32.8% DC and 67.2% EL).

Quarters after surgery (surgery date is at centre of Q0)	CT/other imaging, DC	Minor nose incl. biopsy, DC	Int nose and Minor sinus, DC	Int/Major/Complex sinus, DC	CT/other imaging, EL	Minor nose incl. biopsy, EL	Int nose and Minor sinus, EL	Int/Major/Complex sinus, EL	Polypectomy	TOTAL
**-8**	0.00	0.35	0.00	0.61	0.08	0.57	0.00	0.00	0.00	**1.62**
**-7**	0.03	0.00	0.00	0.00	0.08	0.00	0.00	1.56	0.00	**1.66**
**-6**	0.08	0.00	0.00	0.00	0.08	0.00	0.67	0.00	0.00	**0.82**
**-5**	0.07	0.32	0.00	1.10	0.27	2.07	0.65	1.46	0.00	**5.93**
**-4**	0.14	0.00	0.42	0.53	0.21	0.50	0.00	0.70	0.00	**2.52**
**-3**	0.05	0.00	0.41	0.00	0.23	0.96	0.00	0.68	0.00	**2.33**
**-2**	0.04	0.28	0.00	0.99	0.13	0.46	0.00	3.28	0.00	**5.20**
**-1**	0.02	0.82	0.38	0.48	0.34	0.89	0.00	3.79	0.00	**6.73**
**0**	**0.16**	**8.62**	**43.86**	**243.32**	**0.86**	**29.10**	**110.97**	**680.49**	**0.00**	**1117.37**
**1**	0.00	0.00	0.38	3.36	0.08	0.89	1.11	5.74	0.00	**11.56**
**2**	0.00	0.28	1.17	1.49	0.02	0.00	0.00	3.96	0.00	**6.92**
**3**	0.00	0.29	1.22	1.54	0.02	0.00	0.60	4.10	0.00	**7.77**
**4**	0.00	0.30	0.42	1.59	0.02	0.00	1.84	9.86	0.00	**14.04**
**5**	0.00	0.63	0.87	1.65	0.02	0.00	0.64	1.46	0.00	**5.28**
**6**	0.00	0.00	0.45	1.71	0.03	0.53	0.66	3.78	0.00	**7.15**
**7**	0.00	0.00	1.87	2.36	0.03	0.55	0.69	5.49	0.00	**10.98**
**8**	0.00	0.00	0.48	1.22	0.03	0.57	0.71	2.44	0.00	**5.45**

Table D1. Mean inpatient costs (Admittee Patient Care dataset from Hospital Episode Statistics) per person-quarter (2017-2018 £) - polyps unknown. DC = Day Case; EL = Elective Inpatient; CT = computed tomography; Int = intermediate

For peer review only - http://bmjopen.bmj.com/site/about/guidelines.xhtml

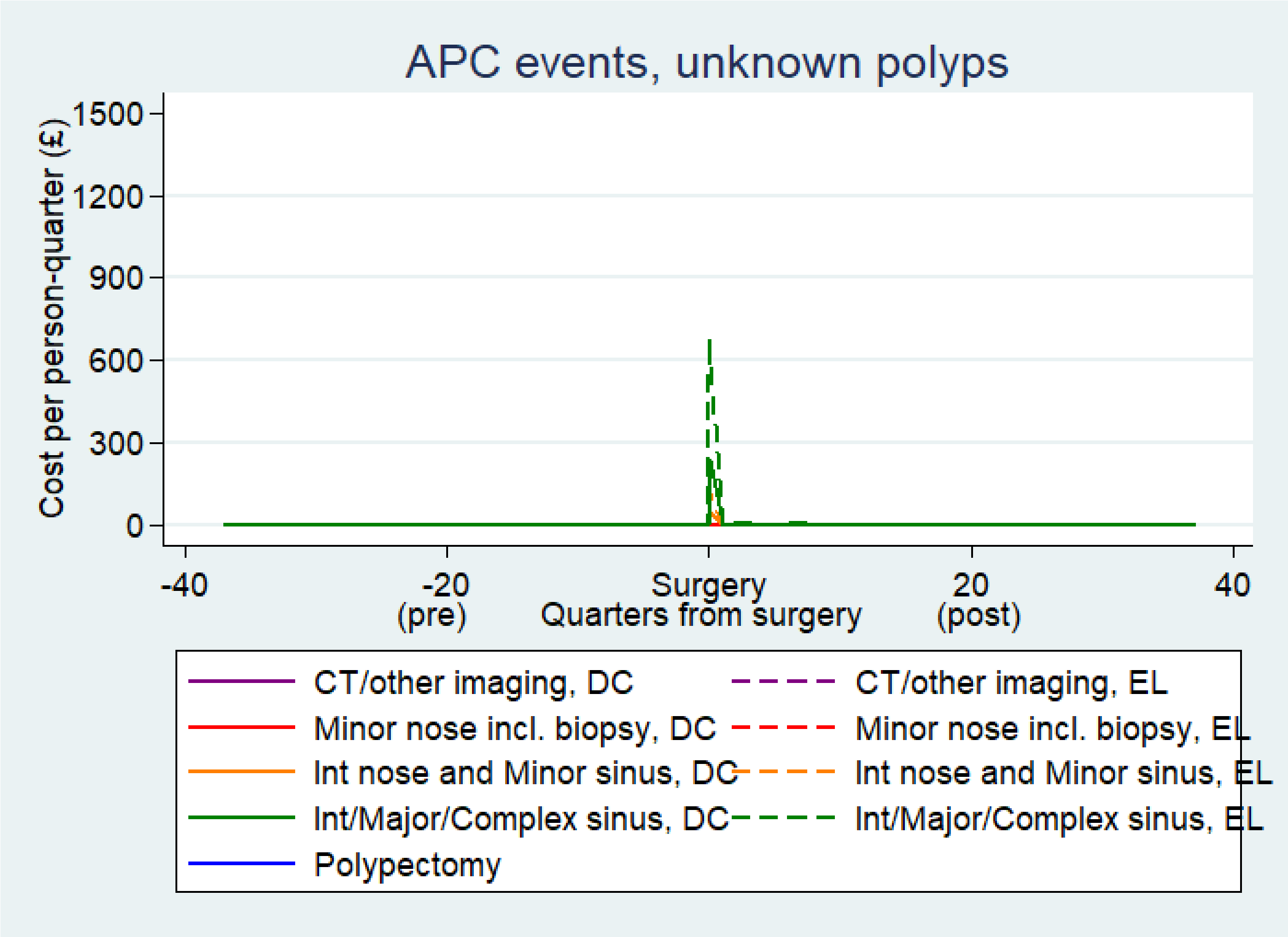
APC events, unknown polyps
Cost per person-quarter (£)
1500
1200
900
600
300
0
-40
-20
(pre)
Surgery
Quarters from surgery
20
(post)
40
CT/other imaging, DC
CT/other imaging, EL
Minor nose incl. biopsy, DC
Minor nose incl. biopsy, EL
Int nose and Minor sinus, DC
Int nose and Minor sinus, EL
Int/Major/Complex sinus, DC
Int/Major/Complex sinus, EL
Polypectomy

10

Quarters after surgery (surgery date is at centre of Q0)	CT/other imaging, DC	Minor nose incl. biopsy, DC	Int nose and Minor sinus, DC	Int/Major/Complex sinus, DC	CT/other imaging, EL	Minor nose incl. biopsy, EL	Int nose and Minor sinus, EL	Int/Major/Complex sinus, EL	Polypectomy	TOTAL
**-8**	0.00	0.00	0.00	0.30	0.09	0.00	0.00	0.00	0.00	**0.39**
**-7**	0.05	0.00	0.00	0.29	0.05	0.27	0.00	1.15	0.00	**1.81**
**-6**	0.05	0.00	0.00	0.00	0.11	0.26	0.33	0.00	0.00	**0.75**
**-5**	0.02	0.15	0.21	0.00	0.08	0.00	0.00	0.36	0.00	**0.83**
**-4**	0.05	0.45	0.00	0.26	0.15	0.49	0.00	0.34	0.00	**1.73**
**-3**	0.06	0.00	0.00	0.00	0.11	0.47	0.00	0.33	0.25	**1.21**
**-2**	0.05	0.00	0.00	0.00	0.13	0.90	0.56	0.32	0.00	**1.96**
**-1**	0.07	0.13	0.37	0.23	0.11	1.30	0.27	0.61	0.47	**3.57**
**0**	**0.02**	**7.85**	**40.09**	**152.60**	**0.14**	**28.44**	**166.57**	**474.22**	**1414.69**	**2284.63**
**1**	0.00	0.39	0.72	1.16	0.02	0.64	1.06	4.37	4.13	**12.49**
**2**	0.00	0.13	0.56	3.34	0.01	0.22	0.55	3.85	7.54	**16.20**
**3**	0.01	0.69	0.96	2.45	0.02	1.13	1.97	7.26	11.16	**25.63**
**4**	0.01	0.28	0.98	2.76	0.02	0.46	0.58	6.09	9.96	**21.15**
**5**	0.00	0.29	0.61	2.07	0.01	0.48	2.97	6.26	13.58	**26.26**
**6**	0.00	0.15	0.00	2.39	0.00	0.49	1.22	5.01	8.18	**17.45**
**7**	0.00	0.15	0.85	2.19	0.01	0.25	1.26	6.61	12.20	**23.53**
**8**	0.00	0.16	1.10	2.53	0.02	0.26	2.27	4.54	12.02	**22.91**

Table D2. Mean inpatient costs (Admittee Patient Care dataset from Hospital Episode Statistics) per person-quarter (2017-2018 £) - polyps positive. DC = Day Case; EL = Elective Inpatient; CT = computed tomography; Int = intermediate

For peer review only - http://bmjopen.bmj.com/site/about/guidelines.xhtml

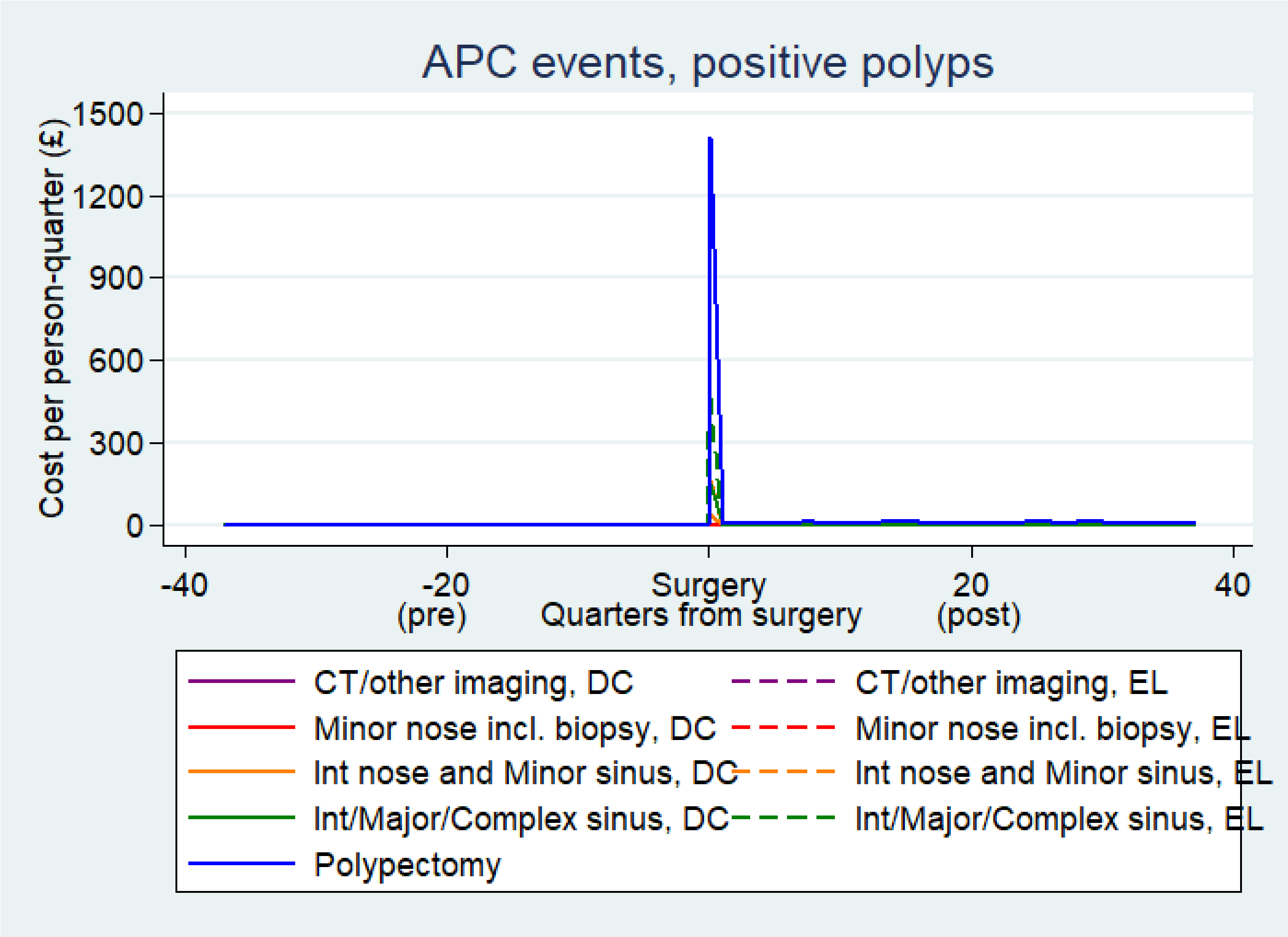
APC events, positive polyps
Cost per person-quarter (£)
1500
1200
900
600
300
0
-40
-20
(pre)
Surgery
Quarters from surgery
20
(post)
40
CT/other imaging, DC
CT/other imaging, EL
Minor nose incl. biopsy, DC
Minor nose incl. biopsy, EL
Int nose and Minor sinus, DC
Int nose and Minor sinus, EL
Int/Major/Complex sinus, DC
Int/Major/Complex sinus, EL
Polypectomy

12

Quarters after surgery (surgery date is at centre of Q0)	CT/other imaging, DC	Minor nose incl. biopsy, DC	Int nose and Minor sinus, DC	Int/Major/Complex sinus, DC	CT/other imaging, EL	Minor nose incl. biopsy, EL	Int nose and Minor sinus, EL	Int/Major/Complex sinus, EL	Polypectomy	TOTAL
**-8**	0.00	0.12	0.00	0.40	0.09	0.19	0.00	0.00	0.00	**0.80**
**-7**	0.04	0.00	0.00	0.19	0.06	0.18	0.00	1.28	0.00	**1.76**
**-6**	0.06	0.00	0.00	0.00	0.10	0.18	0.44	0.00	0.00	**0.77**
**-5**	0.04	0.21	0.14	0.36	0.15	0.68	0.21	0.72	0.00	**2.51**
**-4**	0.08	0.30	0.14	0.35	0.17	0.49	0.00	0.46	0.00	**1.99**
**-3**	0.05	0.00	0.13	0.00	0.15	0.63	0.00	0.44	0.17	**1.58**
**-2**	0.05	0.09	0.00	0.32	0.13	0.76	0.38	1.28	0.00	**3.02**
**-1**	0.06	0.36	0.37	0.31	0.19	1.17	0.18	1.65	0.31	**4.60**
**0**	**0.07**	**8.10**	**41.32**	**181.87**	**0.37**	**28.66**	**148.42**	**540.56**	**952.62**	**1902.00**
**1**	0.00	0.26	0.61	1.88	0.04	0.72	1.08	4.82	2.79	**12.20**
**2**	0.00	0.18	0.76	2.74	0.01	0.15	0.37	3.88	5.11	**13.20**
**3**	0.01	0.56	1.04	2.16	0.02	0.76	1.53	6.23	7.58	**19.89**
**4**	0.01	0.29	0.80	2.39	0.02	0.31	0.98	7.31	6.78	**18.89**
**5**	0.00	0.40	0.69	1.94	0.02	0.32	2.23	4.71	9.27	**19.58**
**6**	0.00	0.10	0.14	2.18	0.01	0.50	1.05	4.61	5.59	**14.18**
**7**	0.00	0.11	1.17	2.24	0.02	0.35	1.08	6.25	8.37	**19.58**
**8**	0.00	0.11	0.91	2.12	0.03	0.36	1.78	3.87	8.26	**17.43**

Table D3. Mean inpatient costs (Admittee Patient Care dataset from Hospital Episode Statistics) per person-quarter (2017-2018 £) – all polyp statuses. DC = Day Case; EL = Elective Inpatient; CT = computed tomography; Int = intermediate

For peer review only

For peer review only - http://bmjopen.bmj.com/site/about/guidelines.xhtml

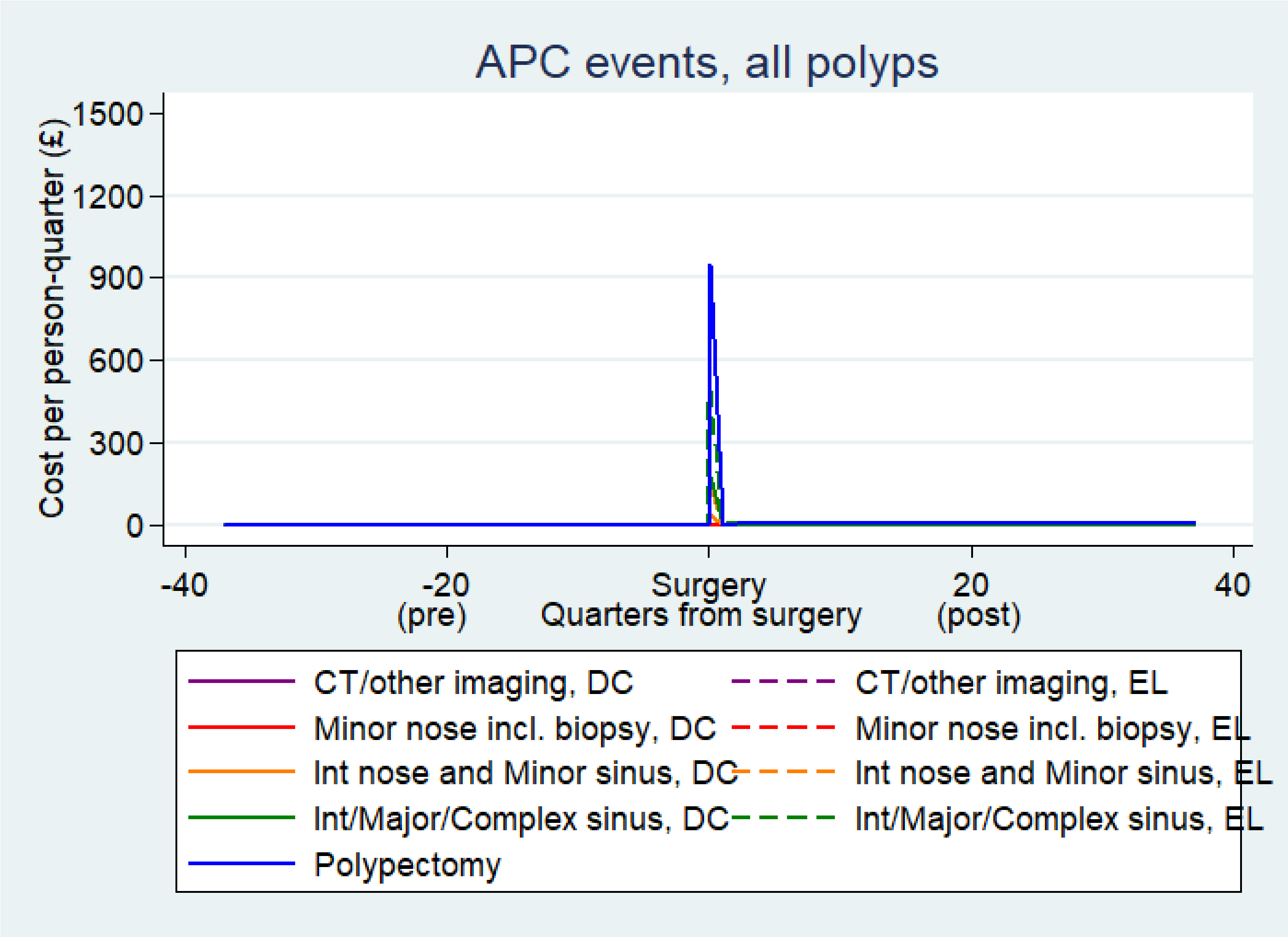
APC events, all polyps
Cost per person-quarter (£)
1500
1200
900
600
300
0
-40
-20
(pre)
Surgery
Quarters from surgery
20
(post)
40
CT/other imaging, DC
CT/other imaging, EL
Minor nose incl. biopsy, DC
Minor nose incl. biopsy, EL
Int nose and Minor sinus, DC
Int nose and Minor sinus, EL
Int/Major/Complex sinus, DC
Int/Major/Complex sinus, EL
Polypectomy

14

E. Section E – Mean outpatient costs per person-quarter, centred on surgery date at midpoint of Q0

Quarters after surgery (surgery date is at centre of Q0)	CT/other imaging	Minor nose incl. biopsy	Polypectomy	Int nose and Minor sinus	Int sinus	Major/ complex sinus	TOTAL
**-8**	8.52	0.80	0.08	3.53	3.79	2.98	**19.69**
**-7**	10.60	1.00	0.03	3.84	5.26	3.43	**24.15**
**-6**	8.69	1.14	0.10	4.01	5.15	1.65	**20.74**
**-5**	9.70	1.03	0.05	4.66	5.60	3.16	**24.21**
**-4**	10.23	0.99	0.16	5.16	6.85	1.51	**24.90**
**-3**	13.62	1.22	0.18	6.06	6.81	3.37	**31.25**
**-2**	16.24	1.64	0.08	7.58	8.27	1.84	**35.67**
**-1**	25.04	1.84	0.44	11.36	12.02	6.17	**56.87**
**0**	**23.03**	**2.93**	**0.85**	**12.47**	**14.46**	**8.66**	**62.41**
**1**	14.88	1.63	0.32	7.57	8.64	2.97	**36.00**
**2**	12.05	1.86	0.12	6.30	6.76	2.04	**29.12**
**3**	9.76	1.53	0.17	6.18	5.75	2.09	**25.48**
**4**	9.36	1.18	0.18	5.40	5.83	2.51	**24.46**
**5**	9.66	0.97	0.15	4.94	5.13	2.58	**23.43**
**6**	9.66	1.18	0.12	4.56	5.95	3.04	**24.50**
**7**	8.91	1.23	0.27	3.90	5.41	1.56	**21.28**
**8**	8.76	1.12	0.13	3.86	5.01	1.61	**20.49**

Table E1. Mean outpatient costs (Outpatient Care dataset from Hospital Episode Statistics) per person-quarter (£) - polyps positive.

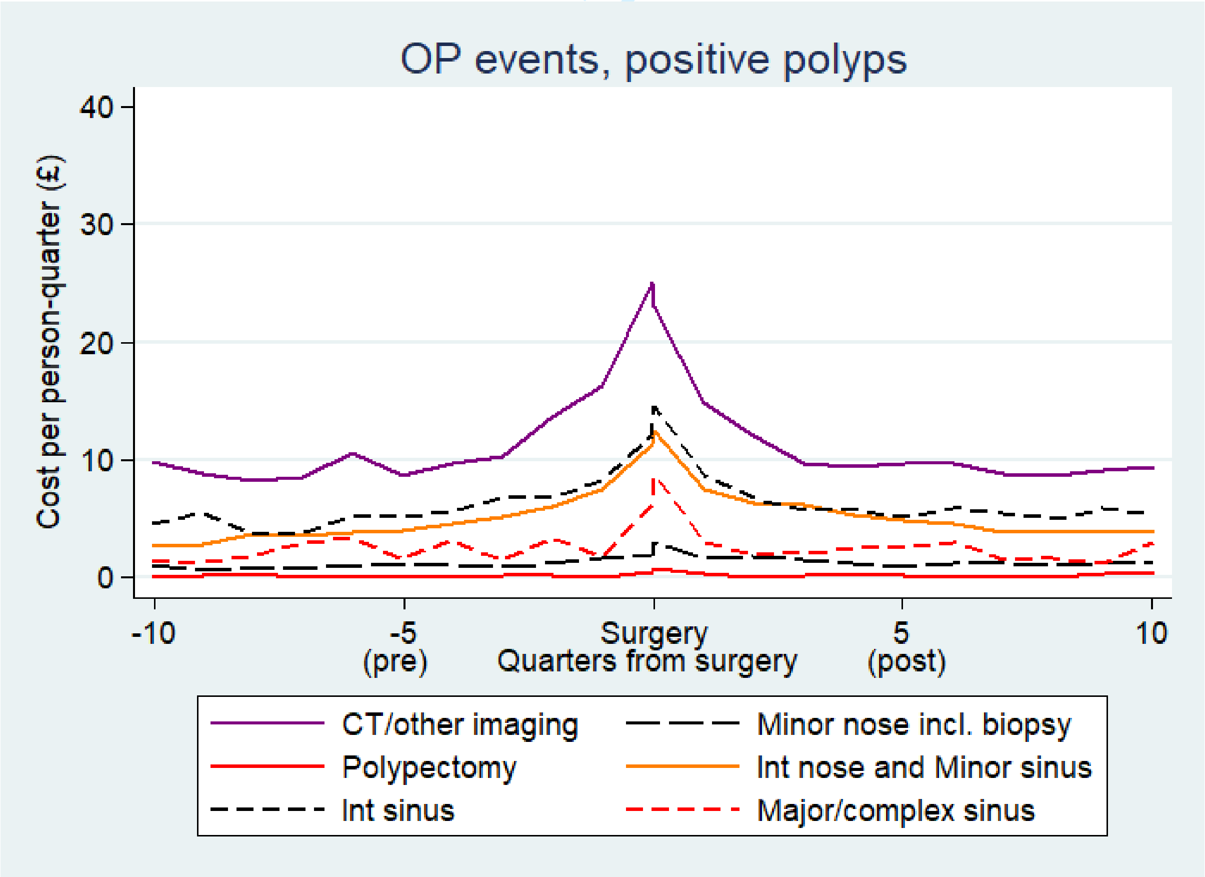

Quarters after surgery (surgery date is at centre of Q0)	CT/other imaging	Minor nose incl. biopsy	Polypectomy	Int nose and Minor sinus	Int sinus	Major/ complex sinus	TOTAL
**-8**	13.55	2.39	-	5.19	7.41	1.13	**29.67**
**-7**	15.59	3.31	-	4.24	7.94	0.00	**31.07**
**-6**	16.30	2.64	-	5.10	7.48	1.04	**32.57**
**-5**	16.35	2.34	-	5.21	7.07	0.00	**30.97**
**-4**	19.16	2.26	-	5.80	9.08	5.83	**42.14**
**-3**	20.40	2.40	-	7.48	9.41	3.72	**43.42**
**-2**	25.25	2.51	-	9.04	11.10	0.90	**48.79**
**-1**	32.30	4.55	-	11.26	14.70	5.18	**67.99**
**0**	**29.11**	**4.37**	**-**	**13.95**	**17.65**	**10.59**	**75.68**
**1**	25.51	3.16	-	12.17	12.35	5.51	**58.70**
**2**	17.85	2.54	-	9.62	9.16	1.43	**40.59**
**3**	15.27	2.41	-	7.26	8.76	2.22	**35.91**
**4**	15.19	1.93	-	6.57	7.83	2.29	**33.81**
**5**	15.00	2.01	-	6.24	7.46	1.59	**32.30**
**6**	13.02	1.57	-	6.59	6.54	4.92	**32.65**
**7**	14.07	1.32	-	5.68	5.89	9.38	**36.33**
**8**	15.98	1.28	-	5.63	5.87	2.65	**31.40**

Table E2. Mean outpatient costs (Outpatient Care dataset from Hospital Episode Statistics) per person-quarter (£) - polyps unknown.

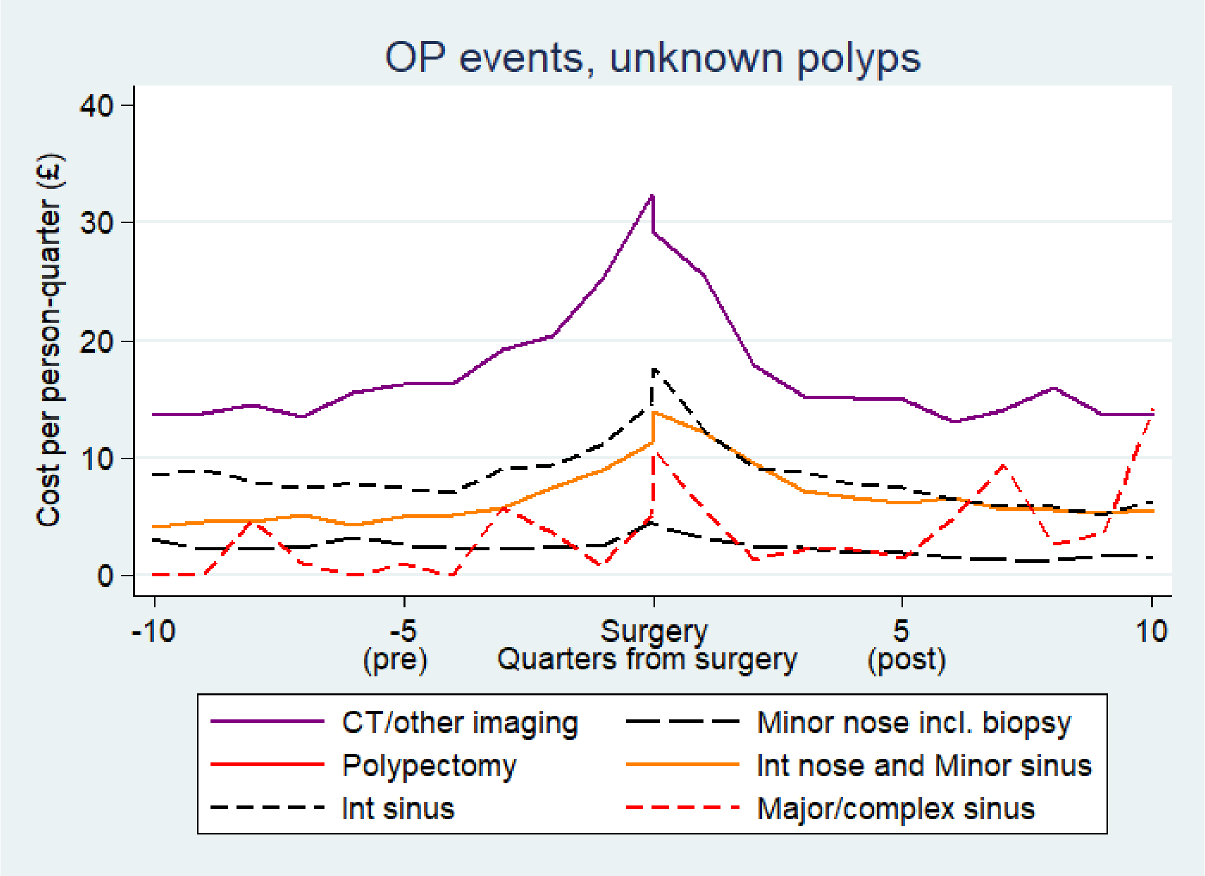

For peer review only - http://bmjopen.bmj.com/site/about/guidelines.xhtml

Quarters after surgery (surgery date is at centre of Q0)	CT/other imaging	Minor nose incl. biopsy	Polypectomy	Int nose and Minor sinus	Int sinus	Major/ complex sinus	TOTAL
**-8**	10.25	1.35	0.05	4.10	5.04	2.34	**23.14**
**-7**	12.32	1.80	0.02	3.98	6.18	2.25	**26.53**
**-6**	11.32	1.66	0.07	4.39	5.95	1.44	**24.82**
**-5**	11.98	1.48	0.03	4.85	6.10	2.08	**26.53**
**-4**	13.28	1.42	0.11	5.38	7.61	2.99	**30.78**
**-3**	15.93	1.62	0.12	6.54	7.70	3.49	**35.40**
**-2**	19.29	1.94	0.06	8.07	9.23	1.52	**40.11**
**-1**	27.49	2.76	0.29	11.33	12.93	5.83	**60.62**
**0**	**25.01**	**3.40**	**0.57**	**12.96**	**15.51**	**9.29**	**66.75**
**1**	18.32	2.13	0.21	9.06	9.84	3.79	**43.35**
**2**	13.92	2.08	0.08	7.37	7.53	1.84	**32.82**
**3**	11.52	1.81	0.12	6.52	6.71	2.13	**28.82**
**4**	11.22	1.42	0.12	5.77	6.47	2.44	**27.45**
**5**	11.35	1.30	0.10	5.35	5.87	2.27	**26.24**
**6**	10.72	1.30	0.08	5.21	6.14	3.63	**27.08**
**7**	10.53	1.26	0.18	4.46	5.56	4.01	**26.00**
**8**	11.02	1.17	0.09	4.41	5.28	1.93	**23.91**

Table E3. Mean outpatient costs (Outpatient Care dataset from Hospital Episode Statistics) per person-quarter (£) – all patients.

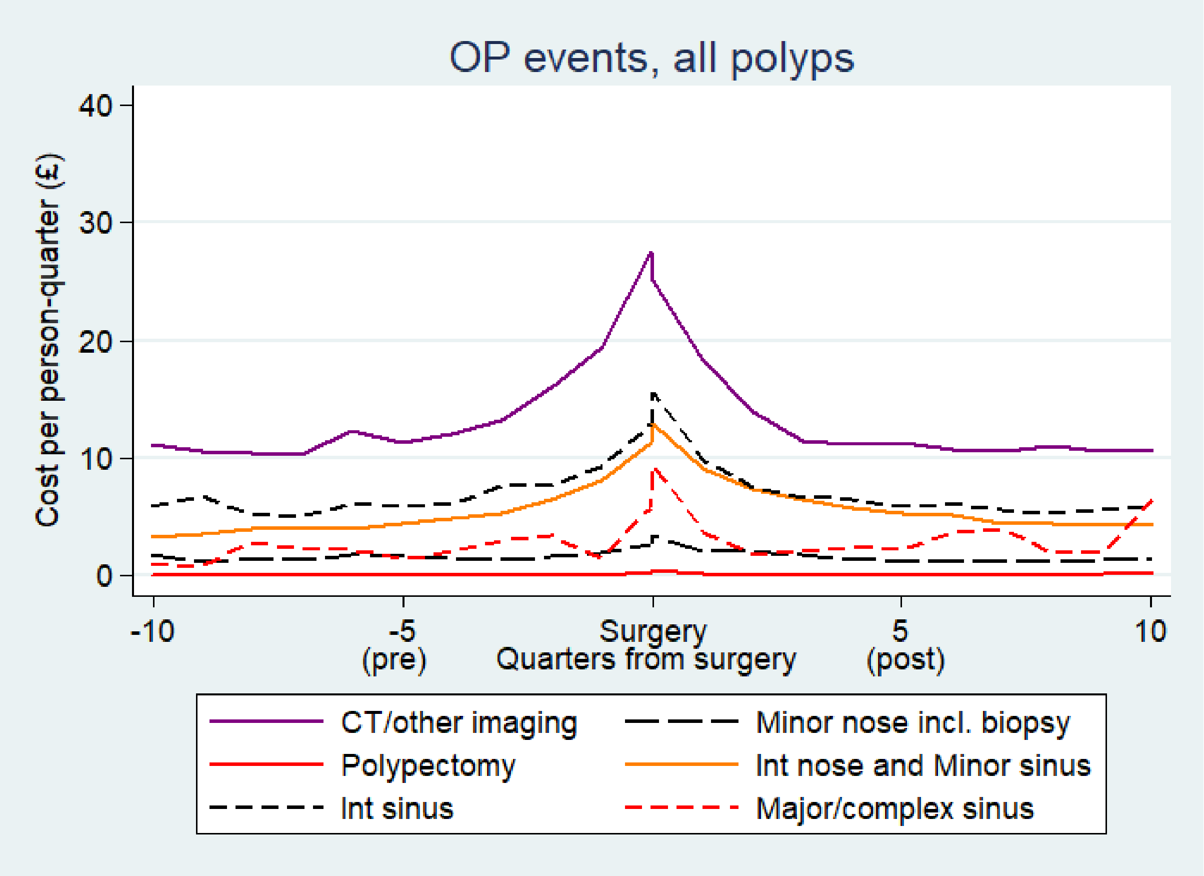

For peer review only - http://bmjopen.bmj.com/site/about/guidelines.xhtml

F. Section F – Mean primary care consultation costs per person-quarter, centred on surgery date at midpoint of Q0

**Quarters after surgery (surgery date is at centre of Q0)**	GP consultation	GP home visit	GP telephone	Nurse consultation	Nurse home visit	Nurse telephone	**TOTAL**
**-8**	7.98	0.03	0.09	0.09	0.01	0.00	**8.20**
**-7**	8.88	0.04	0.10	0.09	0.01	0.00	**9.12**
**-6**	10.45	0.06	0.17	0.13	0.00	0.00	**10.82**
**-5**	12.93	0.04	0.19	0.15	0.01	0.01	**13.31**
**-4**	16.95	0.06	0.21	0.17	0.01	0.01	**17.41**
**-3**	22.30	0.11	0.27	0.26	0.01	0.01	**22.96**
**-2**	27.36	0.11	0.39	0.33	0.02	0.01	**28.23**
**-1**	22.53	0.10	0.44	0.33	0.03	0.01	**23.43**
**0**	**5.36**	**0.01**	**0.12**	**0.09**	**0.01**	**0.00**	5.59
**1**	4.63	0.02	0.11	0.07	0.00	0.01	**4.85**
**2**	4.83	0.05	0.13	0.06	0.01	0.00	**5.09**
**3**	4.47	0.01	0.09	0.07	0.01	0.00	**4.65**
**4**	4.43	0.05	0.11	0.07	0.01	0.00	**4.67**
**5**	4.45	0.02	0.13	0.11	0.01	0.00	**4.72**
**6**	4.36	0.04	0.14	0.07	0.01	0.01	**4.62**
**7**	4.32	0.03	0.13	0.06	0.00	0.00	**4.55**
**8**	4.51	0.01	0.14	0.06	0.01	0.00	**4.72**

Table F1. Mean primary care consultation costs (CPRD dataset) per person-quarter (£) - polyps positive.

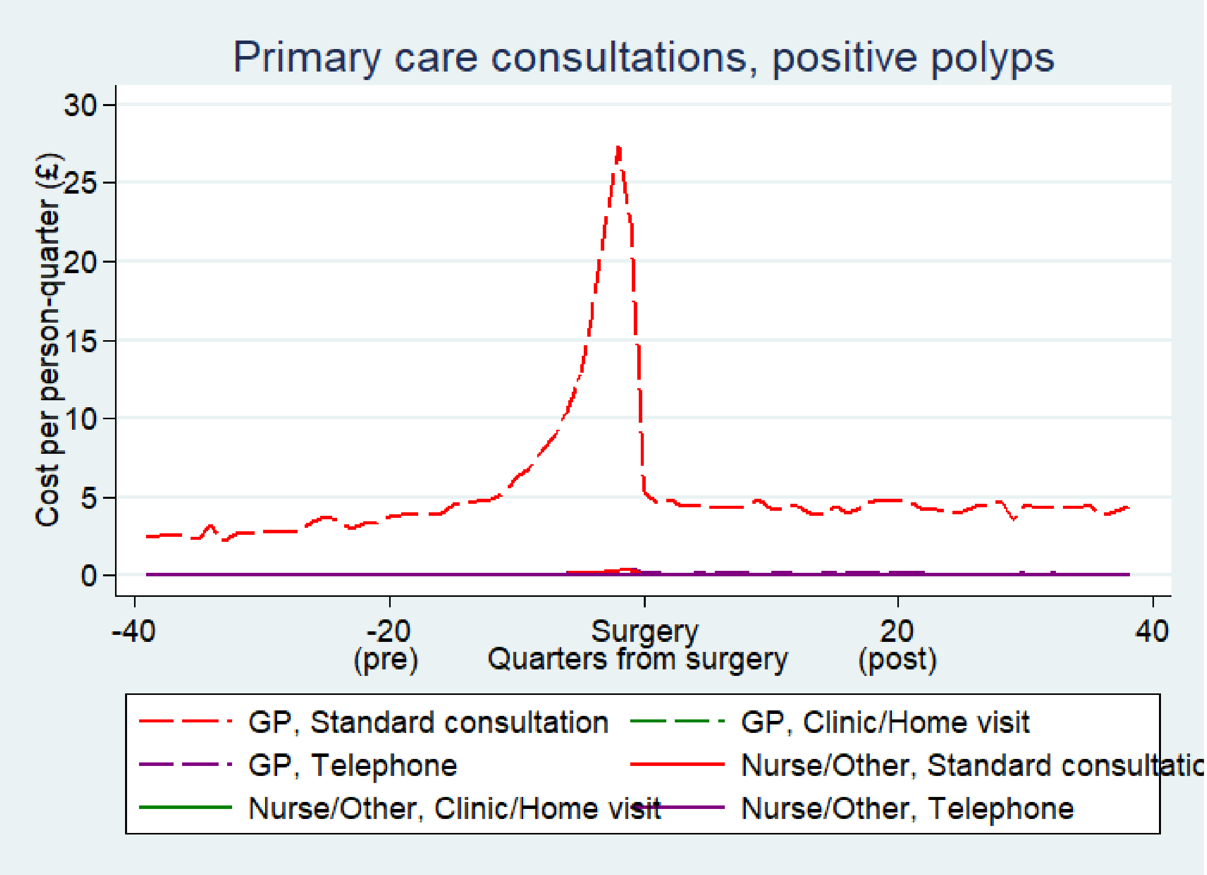

**Quarters after surgery (surgery date is at centre of Q0)**	GP consultation	GP home visit	GP telephone	Nurse consultation	Nurse home visit	Nurse telephone	**TOTAL**
**-8**	8.43	0.02	0.11	0.10	0.01	0.00	**8.68**
**-7**	9.64	0.02	0.14	0.13	0.01	0.00	**9.95**
**-6**	12.32	0.03	0.22	0.13	0.01	0.01	**12.72**
**-5**	14.46	0.03	0.24	0.14	0.01	0.00	**14.89**
**-4**	19.41	0.06	0.31	0.22	0.02	0.01	**20.04**
**-3**	21.60	0.08	0.51	0.28	0.02	0.01	**22.50**
**-2**	21.82	0.07	0.50	0.33	0.02	0.01	**22.76**
**-1**	16.23	0.08	0.52	0.25	0.01	0.01	**17.11**
**0**	**6.69**	**0.01**	**0.22**	**0.11**	**0.01**	**0.00**	**7.04**
**1**	6.77	0.00	0.22	0.12	0.01	0.01	**7.13**
**2**	6.74	0.02	0.09	0.07	0.01	0.01	**6.94**
**3**	5.47	0.02	0.08	0.08	0.00	0.00	**5.66**
**4**	6.57	0.02	0.15	0.07	0.00	0.00	**6.82**
**5**	6.38	0.05	0.12	0.07	0.01	0.00	**6.63**
**6**	5.03	0.03	0.16	0.07	0.01	0.00	**5.31**
**7**	5.85	0.02	0.17	0.05	0.00	0.00	**6.10**
**8**	6.56	0.02	0.17	0.12	0.00	0.00	**6.88**

Table F2. Mean primary care consultation costs (CPRD dataset) per person-quarter (£) - polyps unknown.

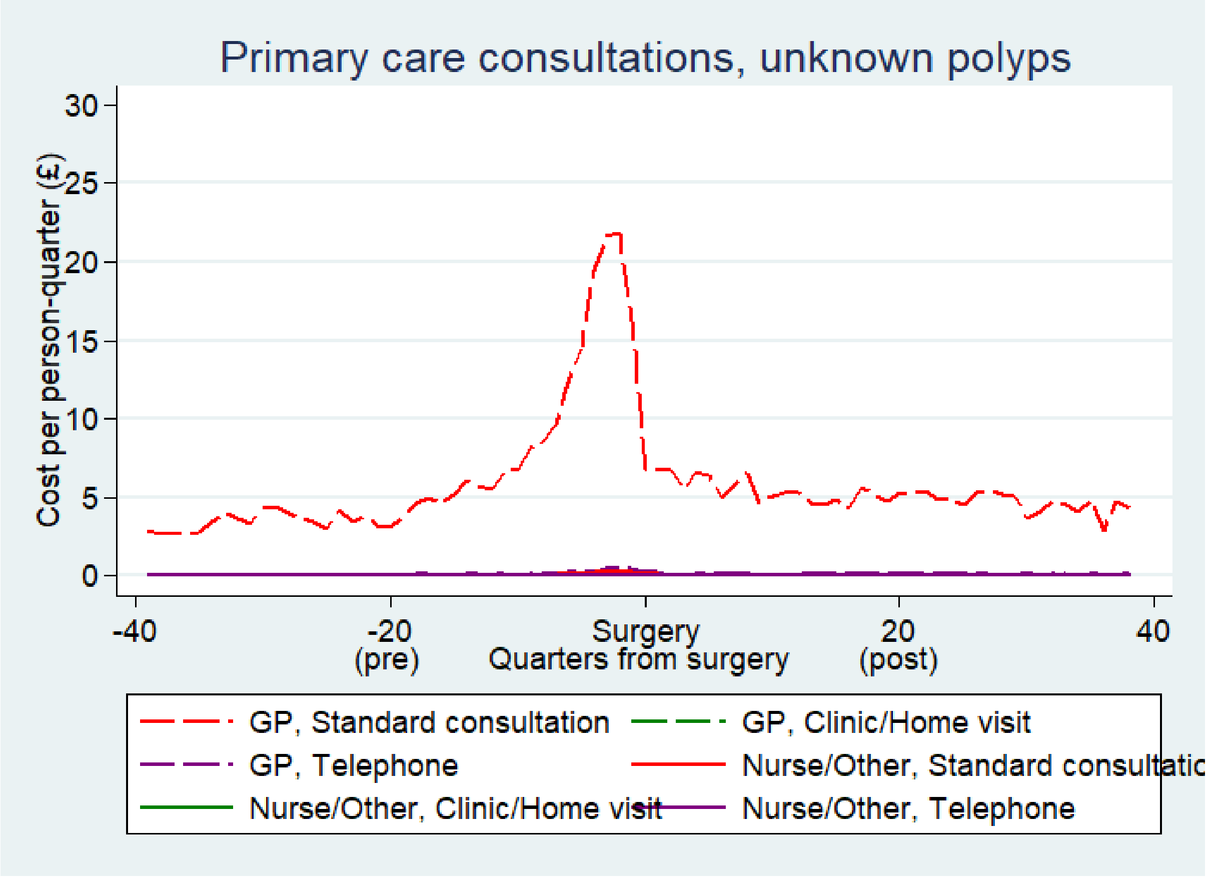

Quarters after surgery (surgery date is at centre of Q0)	GP consultation	GP home visit	GP telephone	Nurse consultation	Nurse home visit	Nurse telephone	TOTAL
**-8**	8.13	0.03	0.10	0.09	0.01	0.00	**8.36**
**-7**	9.13	0.03	0.11	0.10	0.01	0.00	**9.39**
**-6**	11.06	0.05	0.19	0.13	0.01	0.00	**11.45**
**-5**	13.43	0.03	0.20	0.15	0.01	0.01	**13.83**
**-4**	17.75	0.06	0.25	0.19	0.01	0.01	**18.27**
**-3**	22.07	0.10	0.35	0.27	0.02	0.01	**22.81**
**-2**	25.56	0.10	0.42	0.33	0.02	0.01	**26.44**
**-1**	20.47	0.09	0.46	0.31	0.02	0.01	**21.37**
**0**	**5.79**	**0.01**	**0.15**	**0.10**	**0.01**	**0.00**	6.06
**1**	5.32	0.01	0.15	0.09	0.01	0.01	**5.58**
**2**	5.44	0.04	0.12	0.07	0.01	0.00	**5.68**
**3**	4.79	0.02	0.09	0.07	0.01	0.00	**4.97**
**4**	5.11	0.04	0.12	0.07	0.00	0.00	**5.35**
**5**	5.06	0.03	0.13	0.10	0.01	0.00	**5.32**
**6**	4.57	0.03	0.14	0.07	0.01	0.01	**4.84**
**7**	4.80	0.03	0.14	0.06	0.00	0.00	**5.04**
**8**	5.15	0.01	0.15	0.07	0.01	0.00	**5.40**

Table F3. Mean primary care consultation costs (CPRD dataset) per person-quarter (£) – all patients.

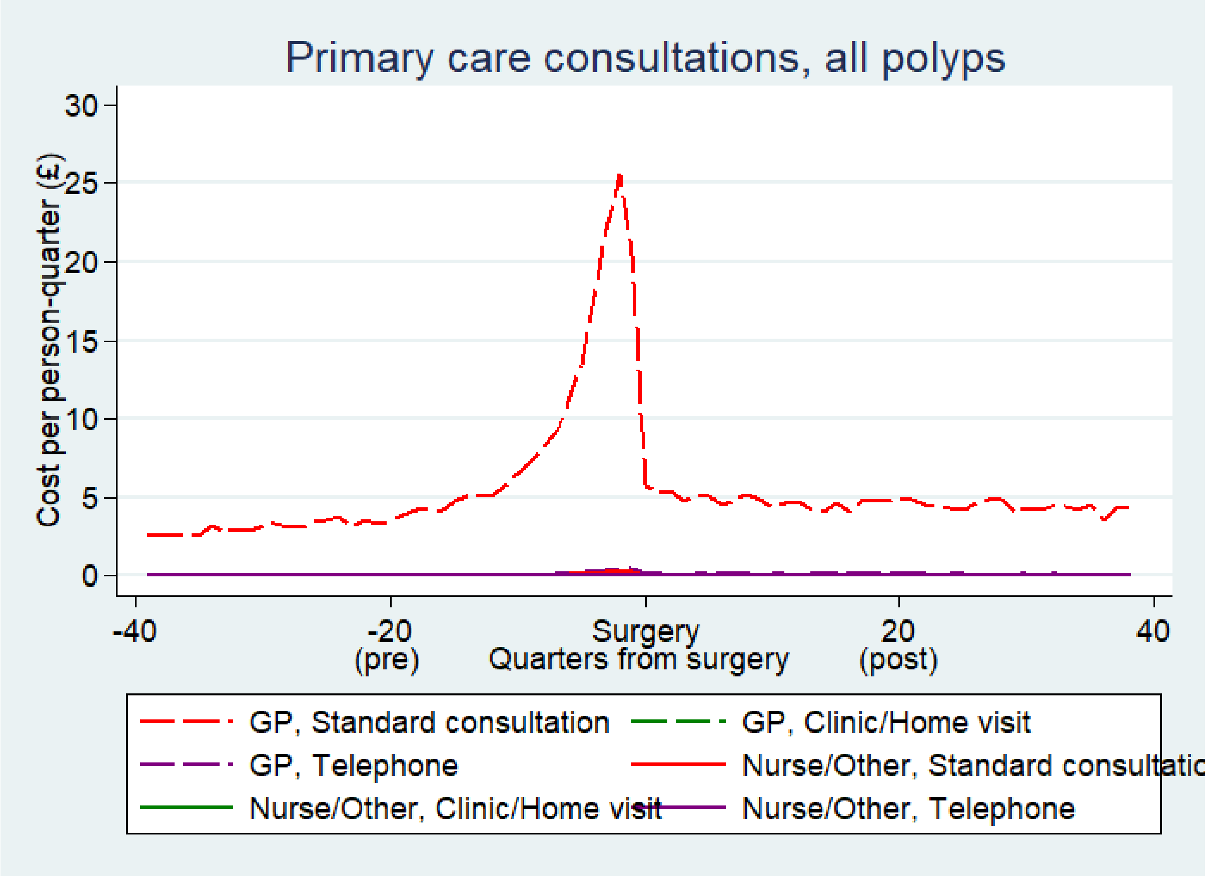

For peer review only - http://bmjopen.bmj.com/site/about/guidelines.xhtml

G. Section G – Mean primary care antibiotic prescription costs per-person-quarter, centred on surgery date at midpoint of Q0

Quarters after surgery (surgery date at centre of Q0)	Cephalo-sporin	Macrolides	Metro-nidazole	Penicillin	Quinolone	Tetra-cycline	Other	**TOTAL**
**-8**	0.07	0.26	0.01	0.18	0.02	0.30	0.03	**0.87**
**-7**	0.08	0.28	0.01	0.18	0.03	0.34	0.04	**0.97**
**-6**	0.08	0.27	0.01	0.20	0.02	0.36	0.03	**0.98**
**-5**	0.09	0.29	0.01	0.21	0.02	0.42	0.04	**1.08**
**-4**	0.09	0.33	0.01	0.22	0.03	0.44	0.04	**1.15**
**-3**	0.09	0.39	0.01	0.24	0.03	0.57	0.05	**1.37**
**-2**	0.10	0.41	0.01	0.25	0.03	0.63	0.04	**1.46**
**-1**	0.07	0.41	0.01	0.24	0.03	0.51	0.04	**1.32**
**0**	**0.09**	**0.31**	**0.01**	**0.21**	**0.03**	**0.31**	**0.04**	**0.99**
**1**	0.07	0.29	0.01	0.19	0.03	0.28	0.04	**0.92**
**2**	0.07	0.29	0.01	0.20	0.03	0.30	0.04	**0.93**
**3**	0.08	0.26	0.01	0.20	0.03	0.32	0.04	**0.95**
**4**	0.08	0.30	0.01	0.19	0.03	0.32	0.05	**0.97**
**5**	0.07	0.25	0.01	0.20	0.03	0.33	0.04	**0.93**
**6**	0.09	0.29	0.01	0.20	0.03	0.31	0.05	**0.97**
**7**	0.08	0.30	0.01	0.19	0.03	0.30	0.04	**0.94**
**8**	0.08	0.28	0.01	0.20	0.03	0.35	0.04	**0.99**

Table G1. Mean primary care antibiotic prescription costs (CPRD dataset) per person-quarter (£) - polyps positive.

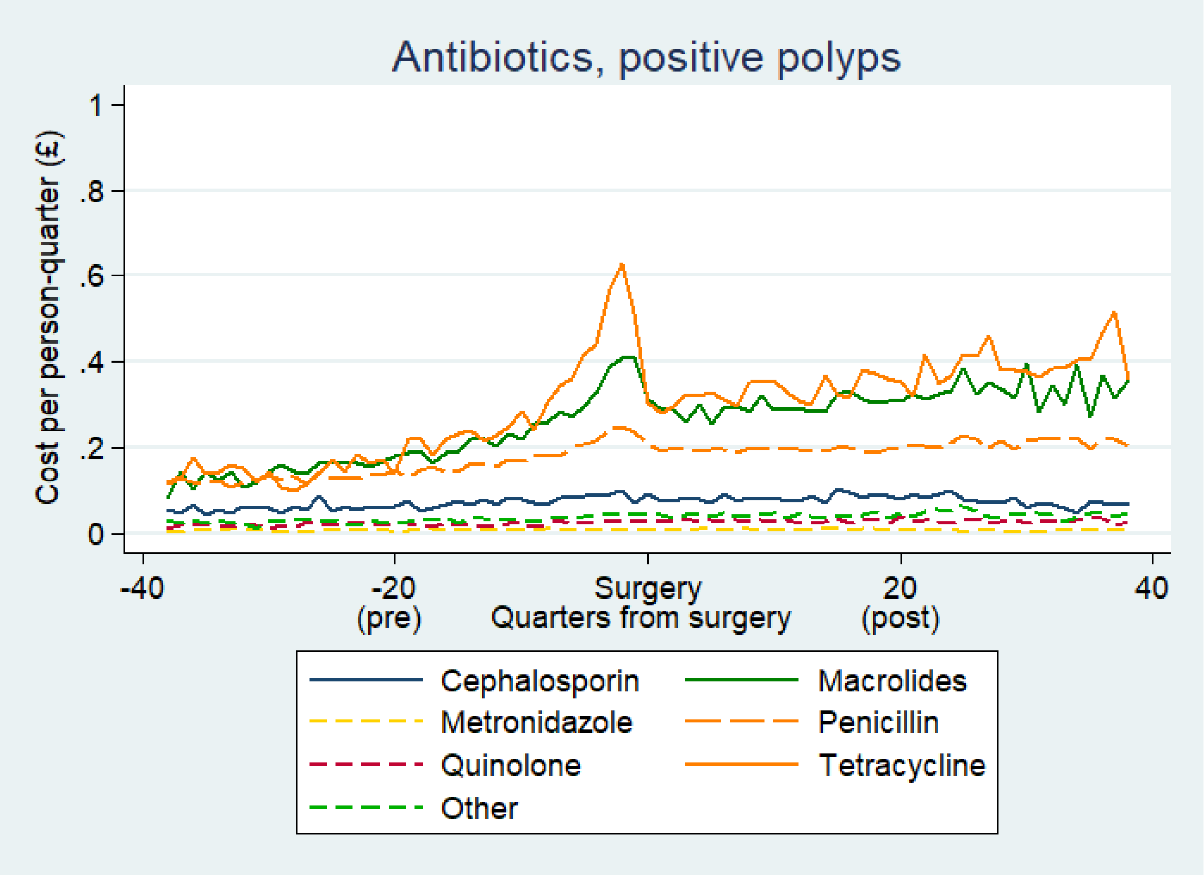

**Quarters after surgery (surgery date at centre of Q0)**	Cephalo-sporin	Macrolides	Metro-nidazole	Penicillin	Quinolone	Tetra-cycline	Other	**TOTAL**
**-8**	0.11	0.29	0.02	0.19	0.02	0.52	0.06	**1.21**
**-7**	0.11	0.32	0.01	0.22	0.02	0.52	0.04	**1.24**
**-6**	0.11	0.43	0.01	0.23	0.03	0.64	0.03	**1.49**
**-5**	0.15	0.42	0.01	0.28	0.03	0.65	0.05	**1.59**
**-4**	0.14	0.52	0.01	0.29	0.04	0.90	0.05	**1.96**
**-3**	0.13	0.62	0.02	0.30	0.04	0.98	0.04	**2.15**
**-2**	0.13	0.60	0.02	0.29	0.04	0.98	0.05	**2.11**
**-1**	0.13	0.54	0.01	0.27	0.03	0.77	0.05	**1.82**
**0**	**0.13**	**0.37**	**0.01**	**0.21**	**0.04**	**0.45**	**0.05**	**1.27**
**1**	0.11	0.35	0.01	0.22	0.04	0.50	0.05	**1.29**
**2**	0.10	0.36	0.02	0.20	0.04	0.50	0.05	**1.26**
**3**	0.10	0.32	0.01	0.19	0.03	0.48	0.04	**1.17**
**4**	0.10	0.35	0.01	0.21	0.03	0.54	0.06	**1.30**
**5**	0.10	0.38	0.01	0.20	0.03	0.47	0.05	**1.24**
**6**	0.08	0.39	0.01	0.18	0.03	0.48	0.05	**1.23**
**7**	0.09	0.36	0.01	0.20	0.03	0.51	0.06	**1.26**
**8**	0.10	0.40	0.02	0.19	0.03	0.52	0.05	**1.30**

Table G2. Mean primary care antibiotic prescription costs (CPRD dataset) per person-quarter (£) - polyps unknown.

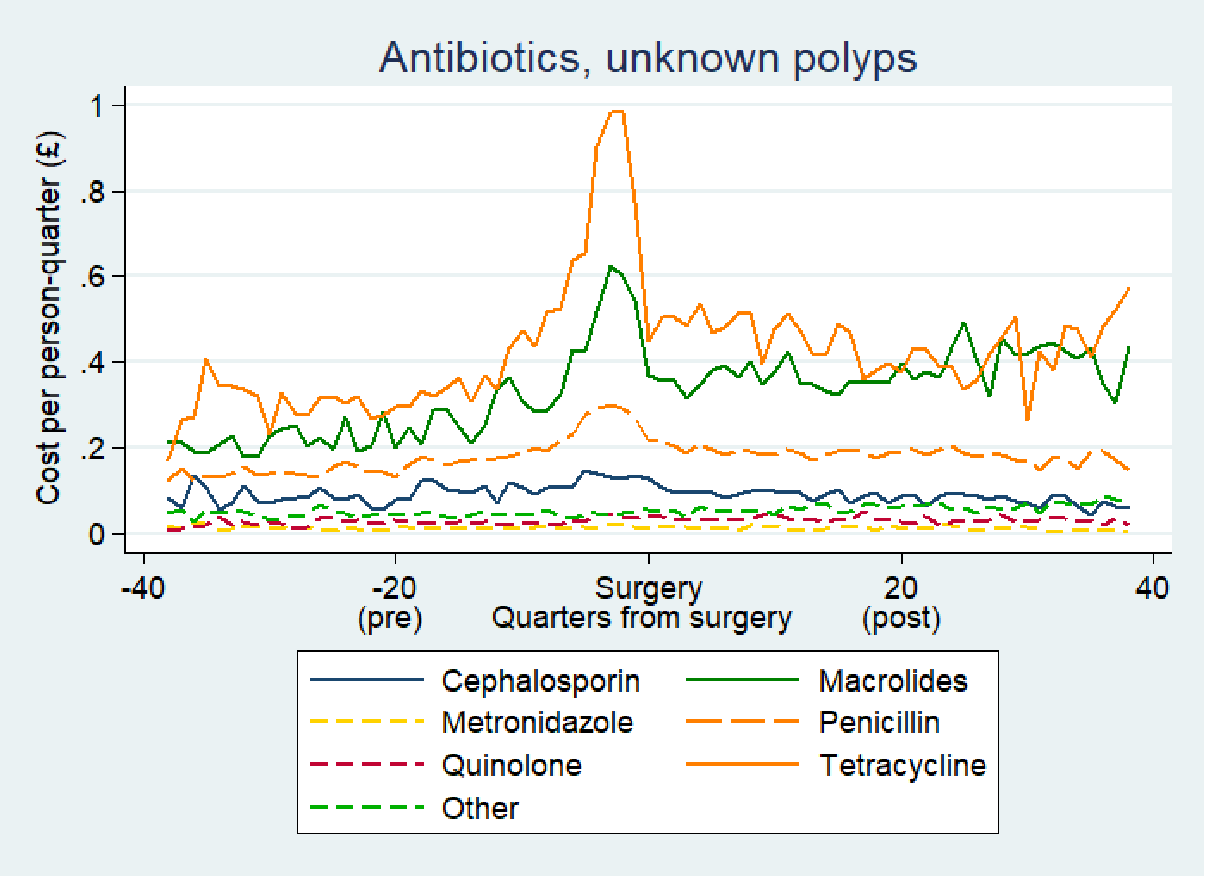

Quarters after surgery (surgery date at centre of Q0)	Cephalo-sporin	Macrolides	Metro-nidazole	Penicillin	Quinolone	Tetra-cycline	Other	TOTAL
**-8**	0.08	0.27	0.01	0.19	0.02	0.37	0.04	**0.98**
**-7**	0.09	0.30	0.01	0.19	0.03	0.40	0.04	**1.06**
**-6**	0.09	0.32	0.01	0.21	0.03	0.45	0.03	**1.15**
**-5**	0.11	0.34	0.01	0.23	0.02	0.50	0.04	**1.25**
**-4**	0.11	0.39	0.01	0.24	0.03	0.59	0.04	**1.41**
**-3**	0.10	0.47	0.01	0.26	0.03	0.71	0.04	**1.62**
**-2**	0.11	0.47	0.01	0.26	0.03	0.74	0.05	**1.68**
**-1**	0.09	0.46	0.01	0.25	0.03	0.60	0.05	**1.48**
**0**	**0.10**	**0.33**	**0.01**	**0.21**	**0.03**	**0.35**	**0.05**	**1.08**
**1**	0.08	0.31	0.01	0.20	0.03	0.35	0.05	**1.04**
**2**	0.08	0.31	0.01	0.20	0.03	0.36	0.04	**1.04**
**3**	0.09	0.28	0.01	0.19	0.03	0.37	0.04	**1.02**
**4**	0.08	0.32	0.01	0.20	0.03	0.39	0.05	**1.08**
**5**	0.08	0.29	0.01	0.20	0.03	0.37	0.04	**1.03**
**6**	0.09	0.32	0.01	0.19	0.03	0.37	0.05	**1.05**
**7**	0.08	0.32	0.01	0.19	0.03	0.37	0.04	**1.04**
**8**	0.09	0.32	0.01	0.20	0.03	0.40	0.04	**1.09**

Table G3. Mean primary care antibiotic prescription costs (CPRD dataset) per person-quarter (£) – all patients.

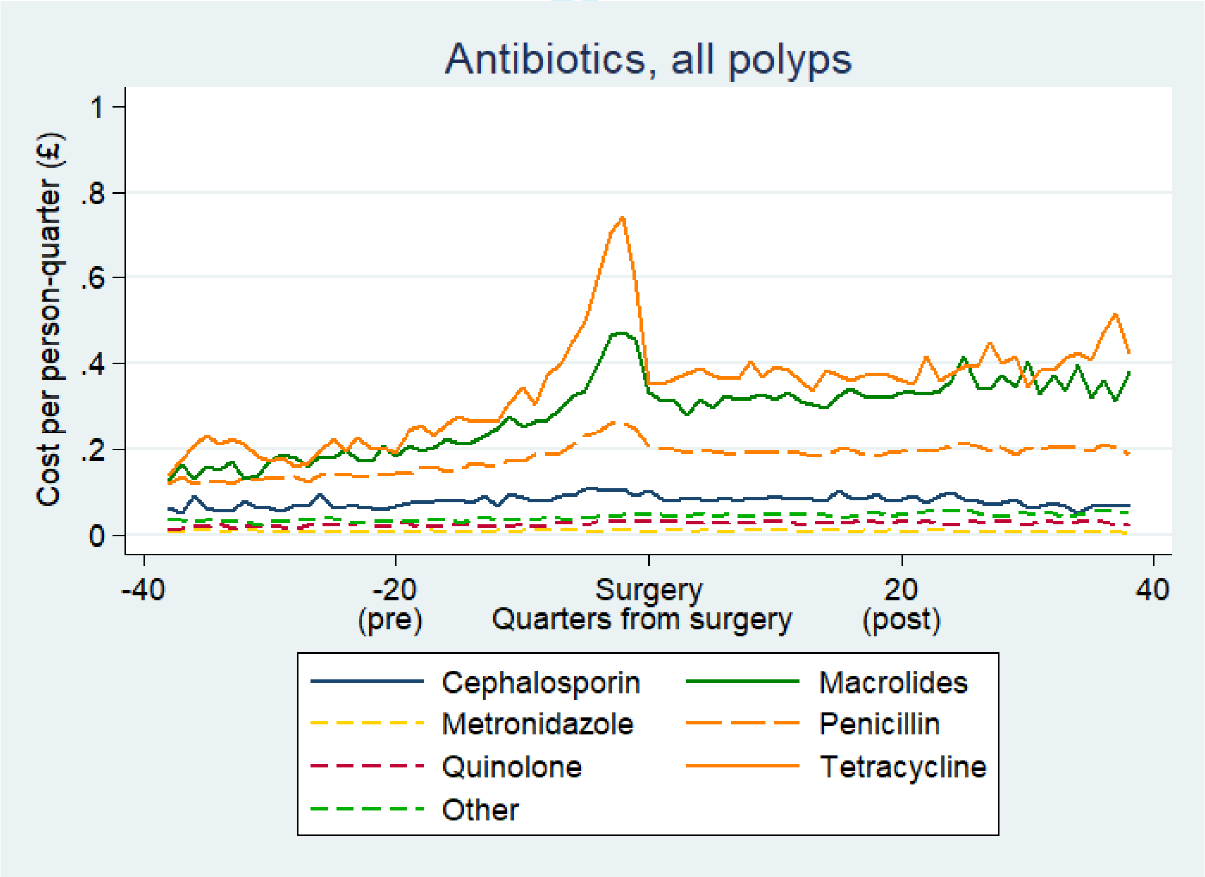

H. Section H – Mean primary care non-antibiotic prescription costs per-person-quarter, centred on surgery date at midpoint of Q0

Quarters after surgery (surgery date at centre of Q0)	Steroids	All other non-abx meds	TOTAL
**-8**	3.80	2.17	**5.97**
**-7**	3.96	2.24	**6.20**
**-6**	4.35	2.25	**6.60**
**-5**	4.89	2.37	**7.26**
**-4**	5.45	2.43	**7.88**
**-3**	6.12	2.56	**8.68**
**-2**	6.84	2.57	**9.41**
**-1**	6.48	2.64	**9.13**
**0**	**5.33**	**2.46**	**7.79**
**1**	5.15	2.51	**7.66**
**2**	5.12	2.60	**7.72**
**3**	4.90	2.69	**7.59**
**4**	4.81	2.67	**7.49**
**5**	4.86	2.76	**7.62**
**6**	4.90	2.82	**7.73**
**7**	4.83	2.82	**7.65**
**8**	4.83	2.79	**7.62**

Table H1. Mean primary care non-antibiotic prescription costs (CPRD dataset) per person-quarter (£) - polyps positive.

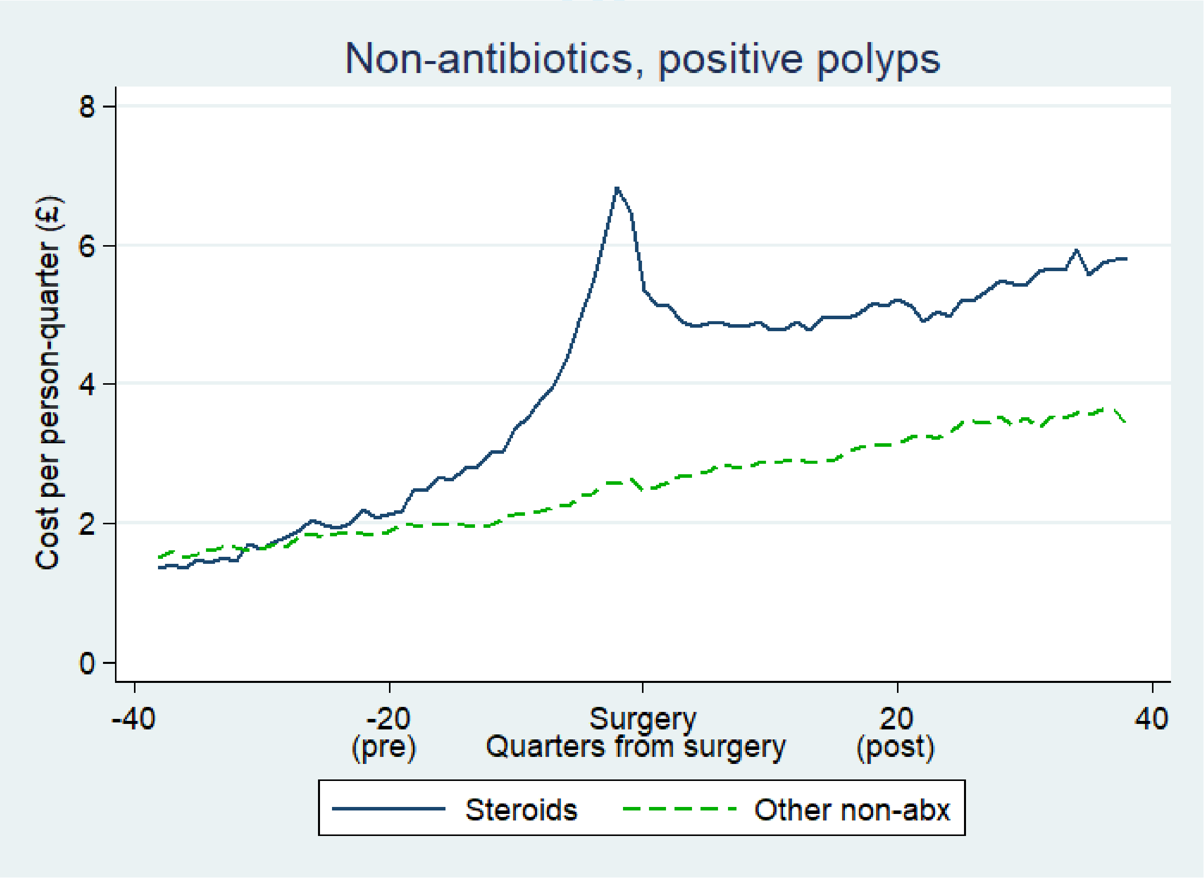

For peer review only - http://bmjopen.bmj.com/site/about/guidelines.xhtml

Quarters after surgery (surgery date at centre of Q0)	Steroids	All other non-abx meds	TOTAL
**-8**	1.96	2.75	**4.70**
**-7**	2.09	2.81	**4.89**
**-6**	2.43	2.82	**5.25**
**-5**	2.76	2.92	**5.68**
**-4**	3.22	3.03	**6.25**
**-3**	3.61	3.03	**6.64**
**-2**	3.76	3.19	**6.96**
**-1**	3.43	3.13	**6.56**
**0**	**2.62**	**2.93**	**5.54**
**1**	2.63	3.00	**5.63**
**2**	2.47	3.02	**5.49**
**3**	2.34	3.04	**5.39**
**4**	2.50	3.04	**5.54**
**5**	2.43	3.12	**5.55**
**6**	2.40	3.05	**5.45**
**7**	2.31	3.05	**5.36**
**8**	2.38	3.18	**5.56**

Table H2. Mean primary care non-antibiotic prescription costs (CPRD dataset) per person-quarter (£) - polyps unknown.

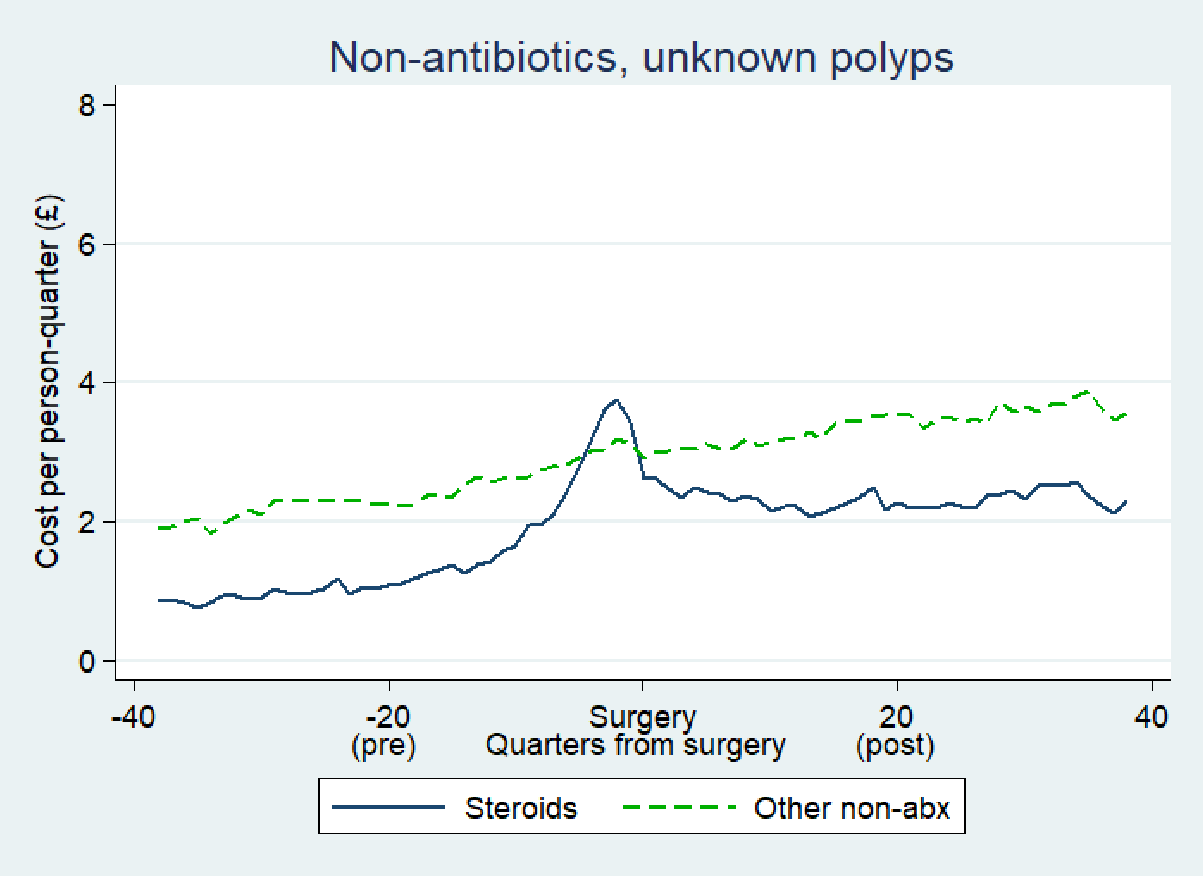

For peer review only - http://bmjopen.bmj.com/site/about/guidelines.xhtml

Quarters after surgery (surgery date at centre of Q0)	Steroids	All other non-abx meds	TOTAL
**-8**	3.19	2.36	**5.55**
**-7**	3.34	2.42	**5.77**
**-6**	3.72	2.44	**6.15**
**-5**	4.19	2.55	**6.74**
**-4**	4.72	2.63	**7.35**
**-3**	5.30	2.71	**8.01**
**-2**	5.84	2.77	**8.61**
**-1**	5.49	2.80	**8.29**
**0**	**4.45**	**2.61**	**7.06**
**1**	4.34	2.67	**7.00**
**2**	4.27	2.73	**7.00**
**3**	4.09	2.80	**6.89**
**4**	4.08	2.79	**6.87**
**5**	4.09	2.87	**6.96**
**6**	4.12	2.89	**7.01**
**7**	4.04	2.89	**6.93**
**8**	4.07	2.91	**6.98**

Table H3. Mean primary care non-antibiotic prescription costs (CPRD dataset) per person-quarter (£) – all patients.

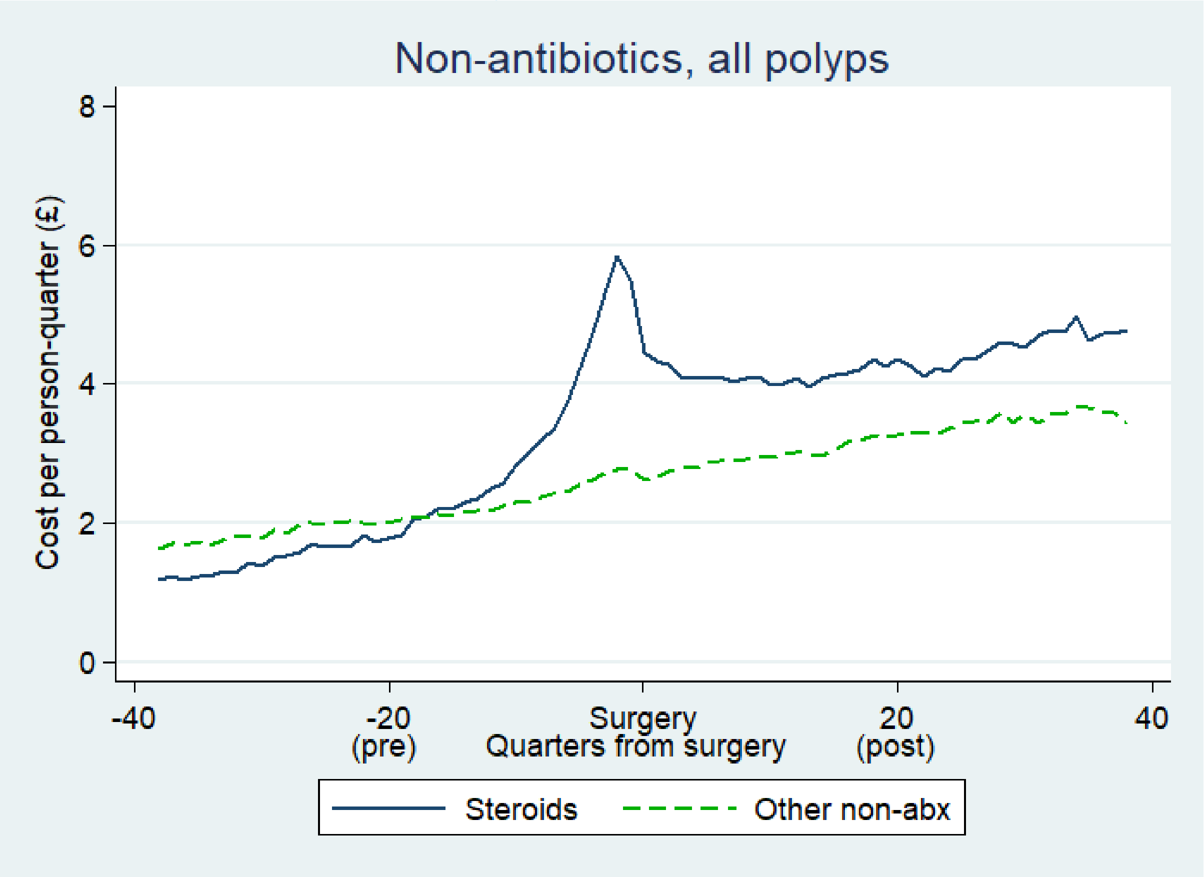

For peer review only - http://bmjopen.bmj.com/site/about/guidelines.xhtml

CHEERS checklist—Items to include when reporting economic evaluations of health interventions

Section/item	Item No	Recommendation	Reported on page No / line No
**Title and abstract**			
Title	1	Identify the study as an economic evaluation or use more specific terms such as "cost-effectiveness analysis", and describe the interventions compared.	Page 1 (note this is not an economic evaluation, it's a cost analysis)
Abstract	2	Provide a structured summary of objectives, perspective, setting, methods (including study design and inputs), results (including base case and uncertainty analyses), and conclusions.	Page 2
**Introduction**			
Background and objectives	3	Provide an explicit statement of the broader context for the study.	Page 4, section 1
		Present the study question and its relevance for health policy or practice decisions.	Page 4, section 1
**Methods**			
Target population and subgroups	4	Describe characteristics of the base case population and subgroups analysed, including why they were chosen.	Section 2.1
Setting and location	5	State relevant aspects of the system(s) in which the decision(s) need(s) to be made.	Section 2.1
Study perspective	6	Describe the perspective of the study and relate this to the costs being evaluated.	Section 2.1
Comparators	7	Describe the interventions or strategies being compared and state why they were chosen.	Section 2.1 (note there is no comparison being made)
Time horizon	8	State the time horizon(s) over which costs and consequences are being evaluated and say why appropriate.	Section 2.2 (note this is only about costs, and consequences are not evaluated)
Discount rate	9	Report the choice of discount rate(s) used for costs and outcomes and say why appropriate.	n/a (section 2.3)

For peer review only

For peer review only - http://bmjopen.bmj.com/site/about/guidelines.xhtml

Section/item	Item No	Recommendation	Reported on page No / line No
Choice of health outcomes	10	Describe what outcomes were used as the measure(s) of benefit in the evaluation and their relevance for the type of analysis performed.	n/a
Measurement of effectiveness	11a	*Single study-based estimates:* Describe fully the design features of the single effectiveness study and why the single study was a sufficient source of clinical effectiveness data.	n/a
	11b	*Synthesis-based estimates*: Describe fully the methods used for identification of included studies and synthesis of clinical effectiveness data.	n/a
Measurement and valuation of preference based outcomes	12	If applicable, describe the population and methods used to elicit preferences for outcomes.	n/a
Estimating resources and costs	13a	*Single study-based economic evaluation:* Describe approaches used to estimate resource use associated with the alternative interventions. Describe primary or secondary research methods for valuing each resource item in terms of its unit cost. Describe any adjustments made to approximate to opportunity costs.	Section 2.2
	13b	*Model-based economic evaluation:* Describe approaches and data sources used to estimate resource use associated with model health states. Describe primary or secondary research methods for valuing each resource item in terms of its unit cost. Describe any adjustments made to approximate to opportunity costs.	n/a
Currency, price date, and conversion	14	Report the dates of the estimated resource quantities and unit costs. Describe methods for adjusting estimated unit costs to the year of reported costs if necessary. Describe methods for converting costs into a common currency base and the exchange rate.	Section 2.2
Choice of model	15	Describe and give reasons for the specific type of decision-analytical model used. Providing a figure to show model structure is strongly recommended.	n/a

For peer review only - http://bmjopen.bmj.com/site/about/guidelines.xhtml

Section/item	Item No	Recommendation	Reported on page No / line No
Assumptions	16	Describe all structural or other assumptions underpinning the decision-analytical model.	n/a
Analytical methods	17	Describe all analytical methods supporting the evaluation. This could include methods for dealing with skewed, missing, or censored data; extrapolation methods; methods for pooling data; approaches to validate or make adjustments (such as half cycle corrections) to a model; and methods for handling population heterogeneity and uncertainty.	n/a
**Results**			
Study parameters	18	Report the values, ranges, references, and, if used, probability distributions for all parameters. Report reasons or sources for distributions used to represent uncertainty where appropriate. Providing a table to show the input values is strongly recommended.	Section 3.1
Incremental costs and outcomes	19	For each intervention, report mean values for the main categories of estimated costs and outcomes of interest, as well as mean differences between the comparator groups. If applicable, report incremental cost-effectiveness ratios.	n/a
Characterising uncertainty	20a	*Single study-based economic evaluation:* Describe the effects of sampling uncertainty for the estimated incremental cost and incremental effectiveness parameters, together with the impact of methodological assumptions (such as discount rate, study perspective).	n/a
	20b	*Model-based economic evaluation:* Describe the effects on the results of uncertainty for all input parameters, and uncertainty related to the structure of the model and assumptions.	n/a
Characterising heterogeneity	21	If applicable, report differences in costs, outcomes, or cost-effectiveness that can be explained by variations between subgroups of patients with different baseline characteristics or other observed variability in effects that are not reducible by more information.	Section 3
**Discussion**			

For peer review only - http://bmjopen.bmj.com/site/about/guidelines.xhtml

Section/item	Item No	Recommendation	Reported on page No / line No
Study findings, limitations, generalisability, and current knowledge	22	Summarise key study findings and describe how they support the conclusions reached. Discuss limitations and the generalisability of the findings and how the findings fit with current knowledge.	Section 4
**Other**			
Source of funding	23	Describe how the study was funded and the role of the funder in the identification, design, conduct, and reporting of the analysis. Describe other non-monetary sources of support.	Page 14
Conflicts of interest	24	Describe any potential for conflict of interest of study contributors in accordance w ith journal policy. In the absence of a journal policy, we recommend authors comply with International Committee of Medical Journal Editors recommendations.	Page 13

For consistency, the CHEERS statement checklist format is based on the format of the CONSORT statement checklist

For peer review only

For peer review only - http://bmjopen.bmj.com/site/about/guidelines.xhtml
